# DABCO-promoted photocatalytic C–H functionalization of aldehydes

**DOI:** 10.3762/bjoc.17.205

**Published:** 2021-12-21

**Authors:** Bruno Maia da Silva Santos, Mariana dos Santos Dupim, Cauê Paula de Souza, Thiago Messias Cardozo, Fernanda Gadini Finelli

**Affiliations:** 1 Instituto de Pesquisas de Produtos Naturais, Universidade Federal do Rio de Janeiro, 373, Carlos Chagas Ave, Rio de Janeiro RJ, 21941-902, Brazil; 2 Instituto de Química, Universidade Federal do Rio de Janeiro 149, Athos da Silveira Ramos Ave, Rio de Janeiro RJ, 21941-909, Brazil

**Keywords:** C–H functionalization, DABCO, HAT, photocatalysis

## Abstract

Herein we present a direct application of DABCO, an inexpensive and broadly accessible organic base, as a hydrogen atom transfer (HAT) abstractor in a photocatalytic strategy for aldehyde C–H activation. The acyl radicals generated in this step were arylated with aryl bromides through a well stablished nickel cross-coupling methodology, leading to a variety of interesting aryl ketones in good yields. We also performed computational calculations to shine light in the HAT step energetics and determined an optimized geometry for the transition state, showing that the hydrogen atom transfer between aldehydes and DABCO is a mildly endergonic, yet sufficiently fast step. The same calculations were performed with quinuclidine, for comparison of both catalysts and the differences are discussed.

## Introduction

The functionalization of inert C–H bonds is a goal pursued by chemists from decades, due to its ubiquity in organic molecules. This strategy also dismisses tiresome protecting groups and functional group interchanging steps, often required in traditional synthetic methodologies [1–2]. The development of photocatalysis enabled inexpensive access to C–H activation methodologies under mild conditions, with hydrogen atom transfer (HAT) reactions standing out as a main strategy [1,3–4]. The hydrogen abstractor is a reactive species, often used in catalytic amounts, capable of promoting a highly selective homolytic cleavage of the C–H bond that results in a carbon-centered radical [5–6].

Nitrogenated structures are easily oxidized under mild conditions into their radical or radical cation forms [7], being very attractive as HAT catalysts as demonstrated by previous works using secondary amides [8–9], sulfonamides [10] and quinuclidine [11–12], the latter being broadly explored in the literature for several functionalizations along with its derivatives [11–20].

**Figure 1:**
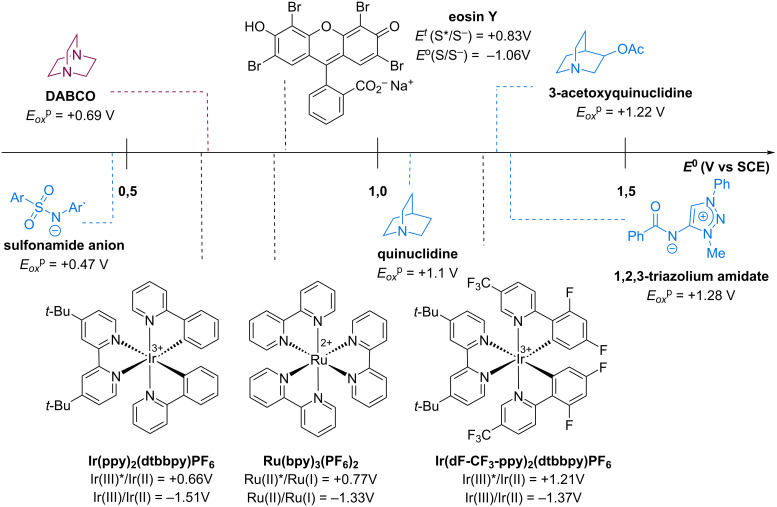
Redox potentials of representative nitrogenated HAT catalysts and photocatalysts [9–12,21–23].

DABCO is a common inexpensive organic base with two nitrogen atoms in a bicyclic cage structure. The interaction between these two nitrogen atoms makes DABCO easier to oxidize and improves the lifetime of the radical cation species when compared to quinuclidine [7]. Investigation of DABCO as a hydrogen abstractor in photocatalytic strategies could expand the catalyst combinations, as illustrated in Figure 1, to create new and exciting methodologies and improve the understanding on theoretical aspects of the HAT process with nitrogen radical cations. However, despite its promising chemical properties and accessibility, it is still underused, and has only recently started to gain attention from the synthetic community.

**Figure 2:**
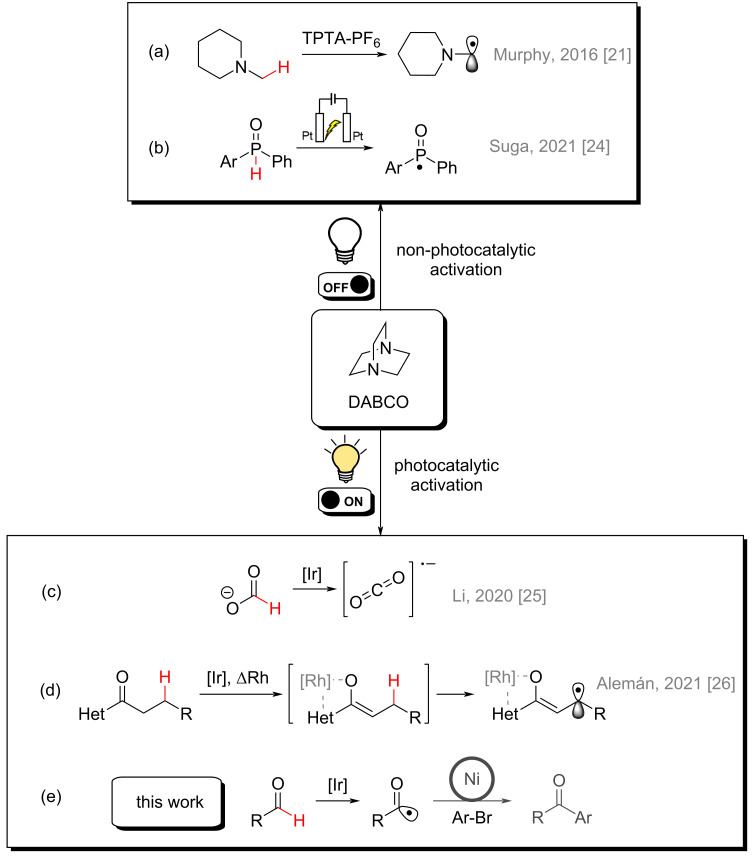
Previous reports of DABCO as hydrogen abstractor in HAT reactions and this work.

Murphy and co-workers reported the use of the DABCO radical cation, generated by a stoichiometric oxidant (TPTA-PF_6_), as a hydrogen abstractor for alpha-nitrogen C–H functionalization [21] (Figure 2a). Suga and co-workers reported an electrochemical approach for P–H bond activation promoted by this reactive species, leading to the synthesis of several phosphacycles [24] (Figure 2b).

Concomitantly with the development of our work, some photocatalytic strategies for DABCO activation emerged. Li and co-workers demonstrated the generation of highly reductant CO_2_ radical anions through potassium formate hydrogen atom abstraction promoted by DABCO [25] (Figure 2c). This species was then used for reduction of (hetero)aryl and alkyl halides, and subsequent carboarylation of several styrenes. Alemán and co-workers also published the use of a photogenerated DABCO radical cation in a distal β-carbonyl enantioselective C–H functionalization for the synthesis of pyrroline derivatives [26] (Figure 2d). The latter is, to the best of our knowledge, the only work reporting a direct substrate C–H bond functionalization using DABCO as a hydrogen atom abstractor in a photocatalytic strategy for synthetic purposes.

In this context, our work aims to broaden the scope of DABCO-promoted photocatalytic C–H functionalization including formyl bonds of aldehydes as substrates (Figure 2e). The acyl radicals generated through this step were used in a well-stablished nickel-catalyzed cross-coupling reaction [19,27–30] with aryl bromides as a proof of concept, leading to the synthesis of aryl ketones. We also present computational calculations of the HAT reaction step with the DABCO radical cation as the hydrogen abstractor and isovaleraldehyde as the substrate. The same calculations were made with quinuclidine, as a well-established bicyclic amine model catalyst, for comparison.

## Results and Discussion

We first investigated the role of inorganic bases through isovaleraldehyde (**1**) coupling with 4-bromobenzonitrile (**2**) under different amounts of DABCO. Two inorganic bases were tested: potassium carbonate (K_2_CO_3_) and sodium hydrogen carbonate (NaHCO_3_). Reactions in the absence of inorganic bases were also performed (Table 1). An excited iridium photocatalyst (Ir[dF(CF_3_)ppy]_2_(dtbbpy)PF_6_) was used for the one-electron oxidation of DABCO into its radical cation, the active species responsible for HAT activation of the aldehyde.

**Table 1:** Optimization of reaction conditions^a^.

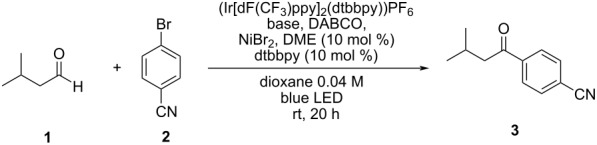			

Entry	Base	DABCO	Yield^b^

1	K_2_CO_3_	0.1 equiv	4%
2		0.5 equiv	66%
3		1.0 equiv	3%

4	NaHCO_3_	0.1 equiv	55%
5		0.5 equiv	81%
6		1.0 equiv	69%
7		0.5 equiv	0%^c^
8		0.5 equiv	0%^d^
9		no DABCO	63%
10		no DABCO	14%^e^
11		quinuclidine	78%^f^

12	no base	0.5 equiv	61%
13		1.0 equiv	69%

^a^(4,4′-Di-*tert*-butyl-2,2′-dipyridyl (dtbbpy, 10 mol %), 4-bromobenzonitrile (1 equiv) and isovaleraldehyde (2 equiv). ^b^Yield determined by ^1^H NMR analysis with 1,3-benzodioxole as internal standard. ^c^Reaction carried out in the absence of light. ^d^Reaction carried out in the absence of iridium photocatalyst. ^e^4-Bromoanisole instead of 4-bromobenzonitrile. ^f^Quinuclidine (0.1 equiv) was used instead of DABCO.

Our results showed that intermediate amounts of DABCO (0.5 equiv) led to the best results (Table 1, entries 2 and 5; see Supporting Information File 1, Table S1 for details). Unexpectedly, the use of either lower (0.1 equiv) or higher (1.0 equiv) concentrations of DABCO led to diminished yields, this effect being very pronounced when using K_2_CO_3_ as the base (Table 1, entries 1 and 3). Although the presence of an inorganic base does not seem to be strictly necessary (Table 1, entries 12 and 13), there was a significant improvement in the yield when using NaHCO_3_ (Table 1, entry 5), even when compared to the results obtained with K_2_CO_3_.

No product was observed when the reaction was performed in the dark (Table 1, entry 7) or in the absence of the iridium photocatalyst (Table 1, entry 8). Surprisingly, decent amounts of product were observed when the reaction was performed without the use of DABCO (Table 1, entry 9). This result indicates an alternative mechanism can be possible for aryl ketone formation when there are no bicyclic amines in the reaction media. Recent works indicate a bromine radical hydrogen abstraction may be possible under similar conditions [31–32]. However, even small amounts of DABCO (Table 1, entry 1) seem to completely shut down this alternative path, probably due to a fast quenching of the excited state photocatalyst, leading to no mechanism competition under the optimized reaction conditions. Also, when other aldehydes or aryl bromides were tested in the absence of DABCO, a very strong dependence on the aryl bromide electronics emerged, leading to diminished yields, remarkably for electron-rich aryl bromides (Table 1, entry 10 and Supporting Information File 1, Scheme S2). These results highlight the relevance of using bicyclic amines as HAT catalyst for the generality of the scope. Quinuclidine was used instead of DABCO, and similar yields were obtained (Table 1, entry 11), as we expected from a previous publication from the MacMillan group [19].

**Scheme 1:**
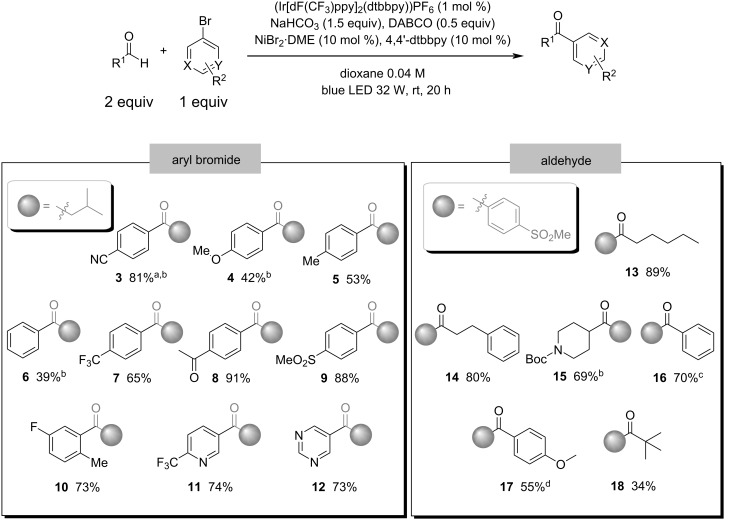
Aryl bromide and aldehyde scope. Isolated yields. ^a^Yield determined by ^1^H NMR analysis with 1,3-benzodioxole as internal standard. ^b^Also performed in the absence of DABCO (see Supporting Information File 1, Scheme S2 for details). ^c^Aldehyde (10 equiv), performed at 40 °C without cooling fan. ^d^Aldehyde (10 equiv).

Once conditions were established, we investigated the generality of the aryl bromide scope using isovaleraldehyde (**1**) as source of acyl radicals. Scheme 1 shows that electron-rich and non-substituted aryl bromides seemed to give only moderate isolated yields (**4–6**, 39–53%), but electron-withdrawing substituted aryl bromides proved to be the best coupling partners for this reaction (**3**, **7–9**, 65–91%). Pyridyl bromides also proved to be very efficient (**11**, **12**, 73–74%). The results also demonstrate that *ortho*-substitution in the aromatic ring poses no impeditive challenge to this reaction (**10**, 73%).

The next step was the study of the aldehyde scope, using 4-bromophenyl methyl sulfone as a coupling partner. We were delighted to see that both aliphatic and aromatic aldehydes could be arylated using this protocol with good to excellent yields (**13–16**, 69–89%), although 4-anisaldehyde led to a diminished yield when compared to benzaldehyde (**17**, 55%). Higher amounts of aromatic aldehydes were required, probably due to the diminished hydricity of their C–H bond and side reactions under photochemical conditions (see Supporting Information File 1, Table S3 for details). Surprisingly, the very bulky trimethylacetaldehyde could also be arylated using the same protocol (**18**, 34%).

**Scheme 2:**
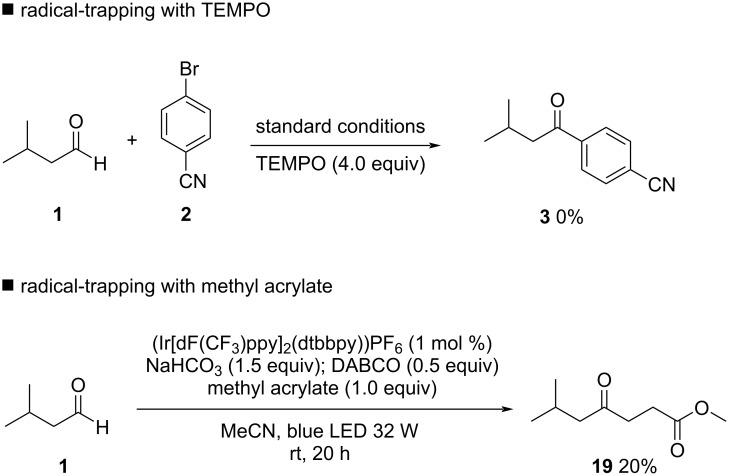
Mechanistic investigations of the HAT reaction using DABCO.

Mechanistic investigations were conducted to support the proposed HAT mechanism by DABCO. Radical trapping with TEMPO completely shuts down product formation, proving a radical mechanism is operating. Moreover, an acyl-TEMPO adduct was formed and could be isolated and characterized, proving the generation of acyl radicals in reaction media. Another evidence of DABCO as HAT catalyst for aldehyde activation could be obtained by the radical trapping with methyl acrylate. In this case, Giese-type product **19** was isolated in 20% yield (Scheme 2, see Supporting Information File 1, Tables S4 and S5 for details).

**Scheme 3:**
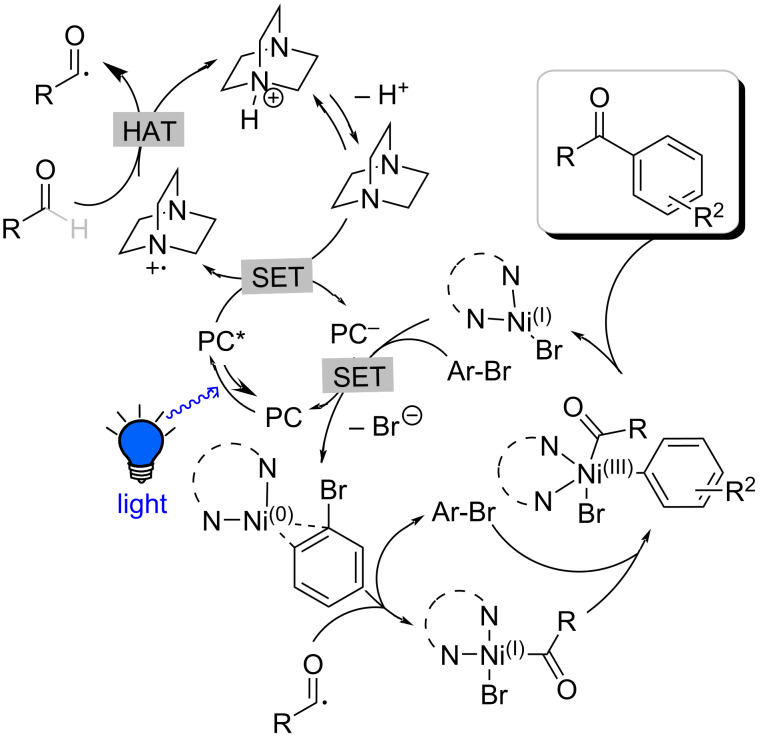
Proposed mechanism for aldehyde arylation. PC = photocatalyst Ir[dF(CF_3_)ppy]_2_(dtbbpy)PF_6._ SET = single-electron transfer event.

A plausible mechanism for the aldehyde arylation is presented in Scheme 3 based on previous literature reports [19,26,33–34] and our mechanistic investigation experiments. Upon blue light irradiation, the photocatalyst is excited generating the strongly oxidizing complex *Ir[dF(CF_3_)ppy]_2_(dtbbpy) (PC*) (*E*_1/2_^red^ [*Ir^III^/Ir^II^] = +1.21 V vs SCE in CH_3_CN). The *Ir(III) excited state is quenched by DABCO (*E*_1/2_^ox^ = +0.69 V vs SCE) producing DABCO radical cation and the reduced Ir(II) complex. Subsequently, DABCO radical cation engage in a HAT event with aldehydes to generate acyl radicals. The coupling of this radical to the Ni(0) complex furnishes the acyl−Ni(I) complex, which then proceeds oxidative addition to aryl bromide to generate the pentavalent Ni(III) complex. Lastly, reductive elimination affords the desired ketone and the Ni(I) complex, which is reduced by the photocatalyst (PC^−^) after coordination to aryl bromide, promoting the turnover of organometallic and photoredox cycles. DABCO is regenerated via deprotonation by an inorganic base.

We also investigated the mechanism operating when the reaction is performed in the absence of bicyclic amines (Table 1, entries 9 and 10 and Supporting Information File 1, Scheme S2). Radical trapping experiments with TEMPO also showed a radical path mechanism, and the acyl radical was detected as an intermediate (Supporting Information File 1, Table S4). The absence of product formation in the Giese addition experiment with methyl acrylate (Supporting Information File 1, Table S5), along with the strong dependence of aryl bromide electronics on the yield indicate the protagonism of the nickel-aryl bromide system. We hypothesized a HAT step by the bromine radical, generated by nickel complex photolysis, in a similar fashion of previous reports [31–32]. However, addition of bromide from external sources led to diminished yields (Supporting Information File 1, Table S6). Also, DFT calculations demonstrated that a HAT reaction between bromine radical and isovaleraldehyde is a barrierless reaction, endergonic by 18.8 kcal·mol^−1^ (gas phase) and 20.1 kcal·mol^−1^ (1,4-dioxane with PCM); almost thrice the Gibbs free energy for the same reaction with DABCO as a HAT abstractor. The barrierless character is supported by NEB-TS calculations (vide infra) (Supporting Information File 1, Figure S16). Both these results do not support this is the operating mechanism and further investigations are underway to determine a plausible path for the reaction in the absence of bicyclic amine catalysts (see Supporting Information File 1, chapter 8 for details).

**Figure 3:**
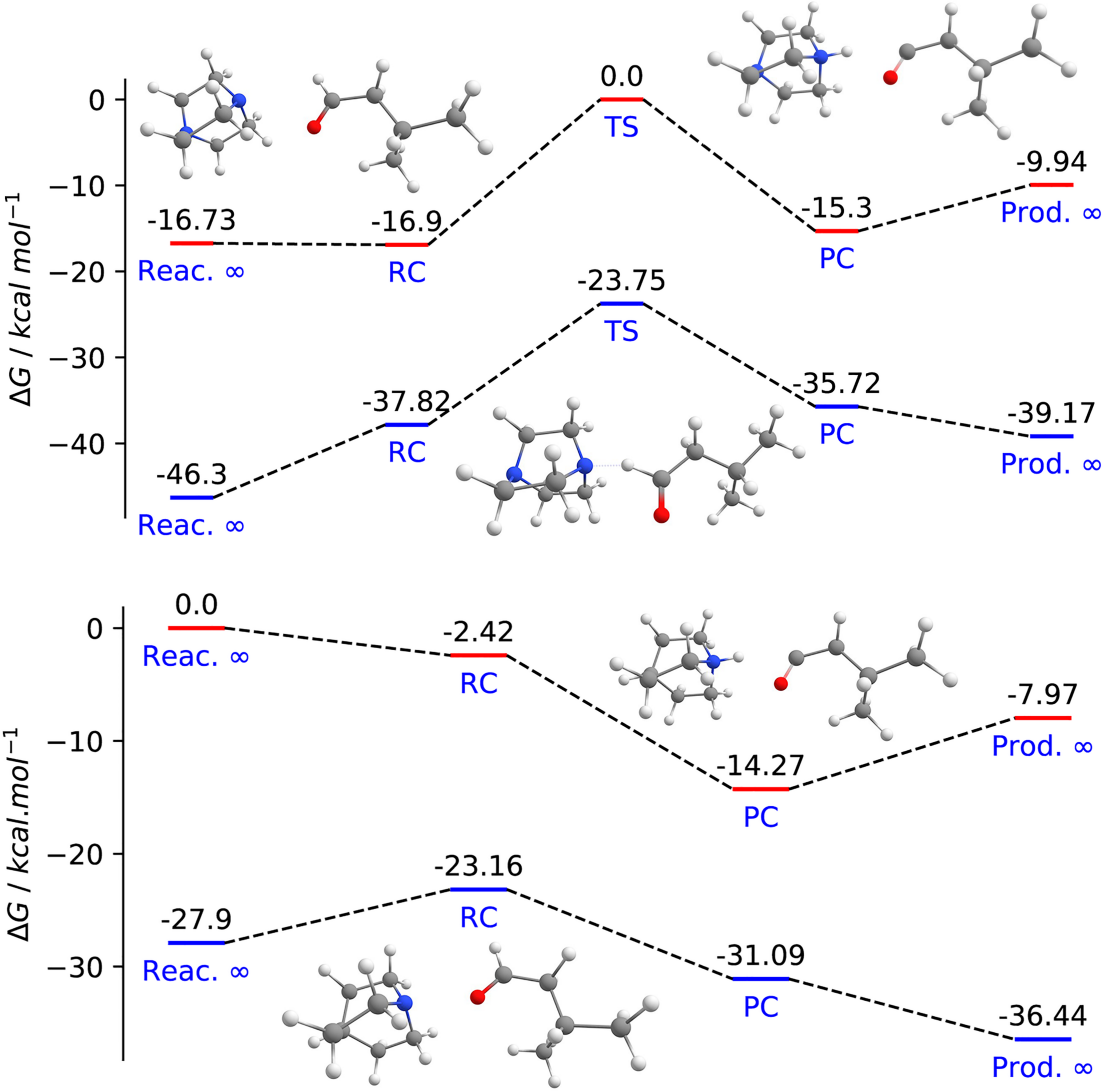
Free energy profile for the HAT step reactions between isovaleraldehyde with (top) DABCO and (bottom) quinuclidine radical cations. The red lines are for the gas phase results while the blue lines are for the PCM single points from the gas phase optimized geometry. RC – reactants complex; PC – product complexes. On both graphs the structure shown on the left represents the RC, on the right, PC and the top centre structure is for the TS between DABCO and the aldehyde.

The HAT step with **1** was investigated by DFT calculations at the DFT/M06-2X/cc-pVTZ level with DABCO and quinuclidine. The energy profiles include the separated reagent molecules (Reac. ∞), the reactants complex (RC), the transition state (TS), the products complex (PC) and the separated products (Prod. ∞). Figure 3 show the calculated Gibbs free energies for the reaction step with **1** in gas phase and in 1,4-dioxane. The HAT reaction step catalyzed with quinuclidine is exergonic (−8.0 kcal·mol^−1^), while the reaction step involving DABCO is endergonic (+6.8 kcal·mol^−1^), in agreement with what would be expected from BDE and BDFE analyses. The solvent effect leads to only minor differences in the thermodynamics of the reaction and do not change the qualitative picture.

**Figure 4:**
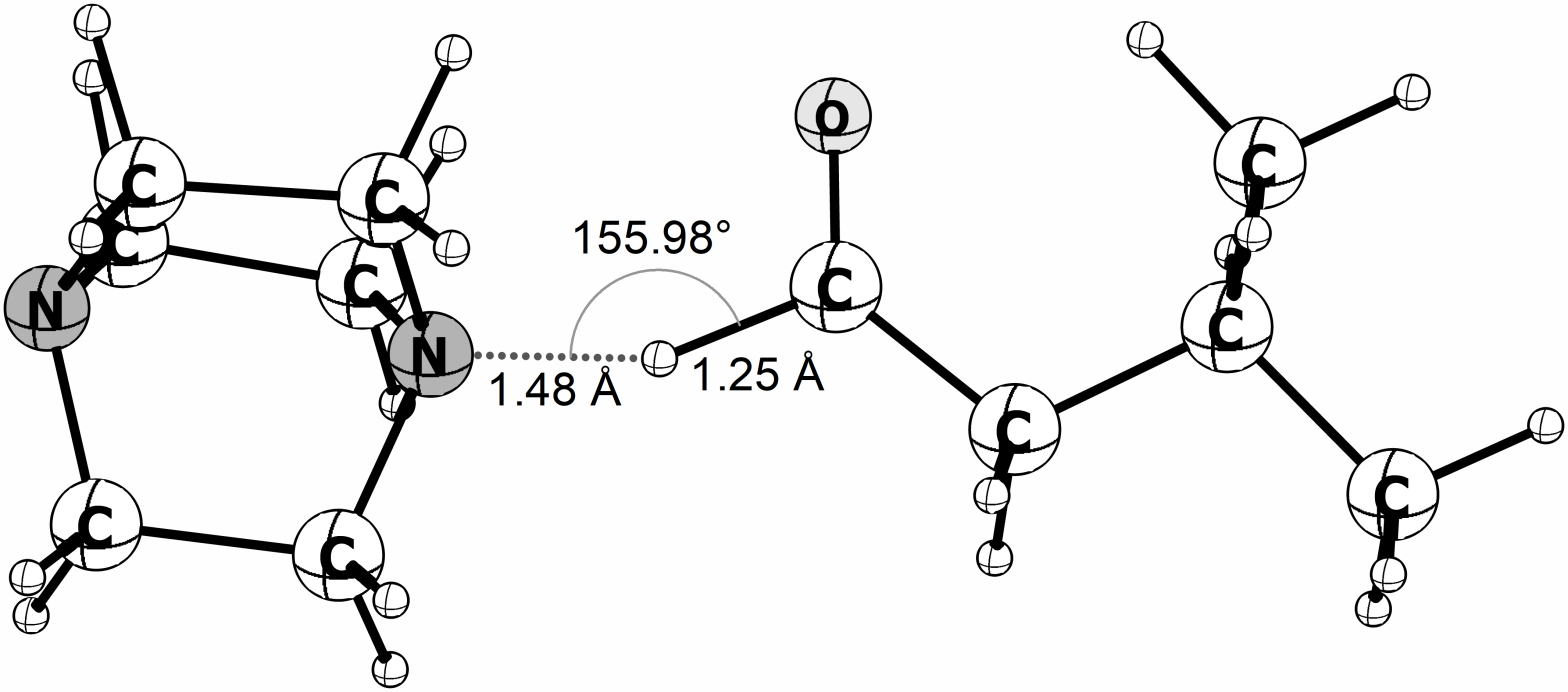
TS structure for the HAT reaction between the DABCO radical cation and isovaleraldehyde obtained at the DFT/M06-2X/cc-pVTZ level. Bond lengths and angle are presented.

The HAT step carried out with DABCO presents a kinetic barrier of 16.9 kcal·mol^−1^ in the gas phase while the quinuclidine-catalyzed reaction is predicted to be barrierless as shown in Figure 3. The energy profiles also show that when the solvent effect is included (blue lines), the RC and RP structures become less stable relative to the isolated reactants and products. As a result, for the DABCO cation radical, the reaction barrier relative to the RC structure is 14.07 kcal·mol^−1^, lower by about 2.8 kcal·mol^−1^ in comparison to gas phase predictions. Figure 4 shows the DABCO TS structure, and the corresponding geometric parameters. The C(=O)–H distance in the optimized aldehyde and the N–H distance in the optimized DABCO–H^+^ cation are 1.11 Å and 1.02 Å, respectively. Comparing these values to the corresponding distances obtained in the TS structure suggests that it is an early TS, as it is structurally closer to the reactants than to the products.

The barrierless character of the HAT reaction step with the quinuclidine radical cation was confirmed with different assessments of the reaction potential energy surface (PES). All procedures resulted in reaction paths with no evidence of any significant barrier, indicating that HAT steps with quinuclidine should proceed at faster rates than with DABCO, possibly at the diffusion limit. (We refer the reader to chapter 8 of Supporting Information File 1 for more details.)

The experimental results with DABCO show that the prediction of a mildly endergonic HAT reaction step and the presence of a barrier are not an obstacle to its ability to act as a catalyst. A naïve use of BDE analysis to select HAT catalysts might suggest that DABCO should not be appropriate for this reaction. However, our results clearly show that DABCO is a perfectly fit for the job. This apparent contradiction can be more easily understood by recalling that a BDE analysis is essentially thermodynamic, but is connected to the kinetics of the reaction step by means of the Evans–Polanyi principle, that relates the rate coefficients of a reaction step to their free energy (for which the BDE difference is a proxy) [35–37]. When considered together with Hammond´s postulate [38], the Evans–Polanyi principle (E-PP) generates the often used rule-of-thumb that good catalyst/substrate pairs are those that present exergonic HAT reaction steps. A more quantitative approach, using the Evans–Polanyi linear relation between Log *k* and the BDE difference allows ranking different catalysts in terms of rate coefficients, but there is no reason to immediately discard endoergic substrate/catalyst combinations. In fact, mildly endoergic combinations should still present sufficiently fast rates for the HAT step, which is precisely the case in our work. Calculations with DABCO and aldehydes **13**, **14** and **17** showed that the reactions follow the qualitative E-PP trend and are all mildly endergonic with barriers lower than the one presented for the reaction with **1** (see Supporting Information File 1, Table S7).

## Conclusion

In conclusion, we report the use of DABCO as a HAT abstractor for aldehyde activation in a photoredox strategy. Several aryl ketones were synthesized with moderate to excellent yields from a range of different aldehydes and aryl bromides, showing the reaction possesses a good tolerance for substrate stereoelectronics.

DABCO is a promising new alternative to expand the toolbox of bicyclic amine abstractors for photoredox HAT reactions, with an appealing low cost, increased accessibility and with structural features that may lead to different reactivities and/or selectivities that are still to be explored. The different electrochemical behaviour of DABCO may also lead to alternative photocatalysts for its activation, such as organic dyes that may not be suitable for other amine catalysts. We expect that the low cost and the ease of handling will drive other groups to explore these possibilities further.

Our work also showed that very different energy profiles for the HAT step can lead to successful reactions, and we propose the use of more flexible selection criterias, expanding the range of catalyst choices. This, in turn, can lead to exciting alternatives in terms of functionalizations, selectivities, and can help to illuminate some of the hazy mechanistic aspects of HAT catalysis with bicyclic amines.

## Supporting Information

File 1 General information, synthetic procedures, additional optimization and mechanistic results, NMR spectra and characterization of compounds and computational details.
