# Supplementary material for: DABCO-promoted photocatalytic C–H functionalization of aldehydes
Source: Beilstein J Org Chem. 2021 Dec 21;17:2959–67. doi: 10.3762/bjoc.17.205 (PMC8712972; doi:10.3762/bjoc.17.205)
Supplement: File 1 — General information, synthetic procedures, additional optimization and mechanistic results, NMR spectra and characterization of compounds and computational details. [file Beilstein_J_Org_Chem-17-2959-s001.pdf]

BEILSTEIN JOURNAL OF ORGANIC CHEMISTRY

Supporting Information

for

DABCO-promoted photocatalytic C–H functionalization of aldehydes

Bruno Maia da Silva Santos, Mariana dos Santos Dupim, Cauê Paula de Souza, Thiago Messias Cardozo and Fernanda Gadini Finelli

Beilstein J. Org. Chem. **2021,** *17,* 2959–2967. doi:10.3762/bjoc.17.205

General information, synthetic procedures, additional optimization and mechanistic results, NMR spectra and characterization of compounds and computational details

License and Terms: This is a supporting information file under the terms of the Creative Commons Attribution License (https://creativecommons.org/licenses/by/4.0). Please note that the reuse, redistribution and reproduction in particular requires that the author(s) and source are credited and that individual graphics may be subject to special legal provisions.
The license is subject to the *Beilstein Journal of Organic Chemistry* terms and conditions: (https://www.beilstein-journals.org/bjoc/terms)

Table of contents

1) General information: S3

2) Procedure for optimization studies S3

3) General procedure for aldehyde arylation S7

4) Aryl bromide scope S8

5) Aldehyde scope S13

6) NMR Spectra S17

7) Mechanistic experiments S33

8) Computational details S43

9) References S62

1) General information:

Solvents and commercially available reagents were purchased from standard chemical suppliers and used without further purification, unless otherwise stated. Anhydrous 1,4-dioxane was obtained by treatment with sodium and distillation before being kept under argon atmosphere. All liquid aldehydes were distilled prior to use. Reactions were sparged and performed under argon atmosphere. All reactions conducted with light irradiation were performed with a 32W blue LED Joyshine model Par38 with 12 spots. Chromatographic purification was accomplished through flash chromatography on silica gel (200–400 Mesh) with mobile phases described in each experimental procedure. Thin layer chromatography (TLC) was performed on Silicycle F-254 aluminium sheets coated with silica gel. Visualization of the chromatogram was performed with UV light quenching and/or chemical staining with $KMnO_4$ or phosphomolybdic acid. The melting points were determined using a Marte PFD III instrument with a 0.1 °C precision from Marte Científica. ^{1}H and ^{13}C NMR spectra were recorded either on a Varian MR-400 (for 400 MHz/100 MHz spectra) or on a Varian VNMRSYS-500 (for 500MHz / 126MHz spectra). Chemical shifts are expressed as parts per million (ppm) with TMS or residual protic deuterated solvent as internal standard. Coupling constants are expressed in Hertz, with multiplicity expressed using standard abbreviations. In ^{13}C NMR data, reported multiplicities are related to carbon-fluorine coupling.

2) Procedure for optimization studies

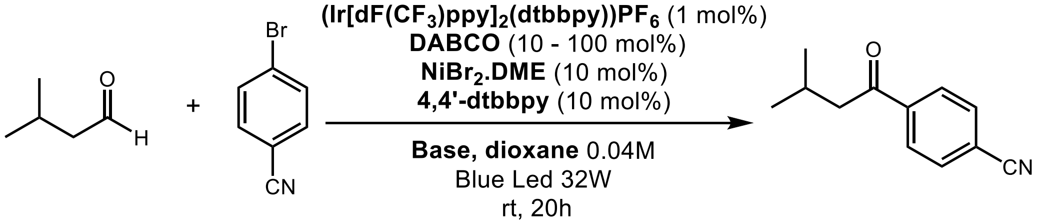

To a 5 mL glass vial were added $NiBr_2$·DME (3.7 mg, 0.012 mmol, 0.11 equiv), 4,4'-di-*tert*-butyl-2,2'-bipyridine (dtbbpy, 3.2 mg, 0.012 mmol, 0.11 equiv) as ligand and 3 mL of dry 1,4-dioxane. This precatalyst vial was sealed and sonicated for around 15 minutes, until its content became homogeneous.

To a separated 5 mL vial equipped with a magnetic stir bar were added the photocatalyst Ir[dF(CF_3)ppy]$_2$(dtbbpy)PF_6 (1.2 mg, 0.0011 mmol, 1 mol %), 1,4-diazabicyclo[2.2.2]

octane (DABCO) (10–100 mol %), an inorganic base and 4-bromobenzonitrile (20 mg, 0.11 mmol, 1 equiv). The precatalyst was syringed into the reaction vial before sparging with argon for 15 minutes. Isovaleraldehyde (23 μL, 0.22 mmol, 2 equiv) was added and the vial was sealed with parafilm. The reaction was magnetically stirred and irradiated for 20 h with a blue LED with a cooling fan to keep the reaction at room temperature as shown in Figure S1 (vials were placed 6 cm away from the light source). After, the reaction was quenched by exposure to air, before 1,3-benzodioxole (11 μL, 0.11 mmol, 1 equiv) was added as internal standard and the yield was determined by ^{1}H NMR analysis.

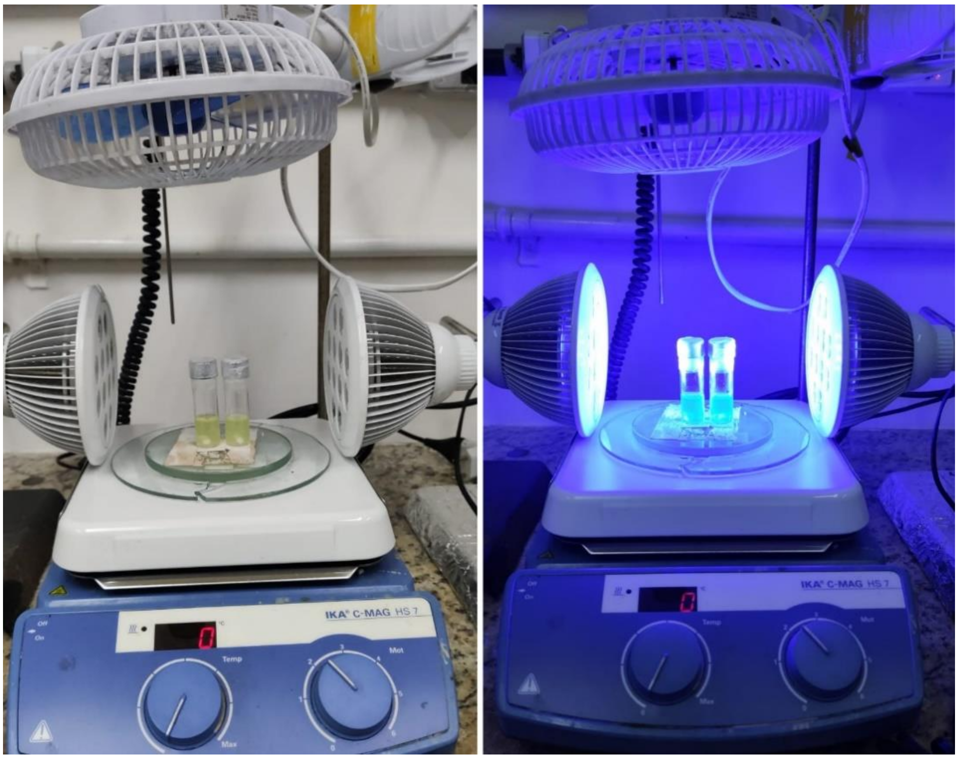

Figure S1: Experimental setup for photoredox reactions. **Optimization of reaction conditions**

Table S1: Reaction conditions optimization studies. Reactions were conducted according to the general procedure, evaluating the effect of different bases and amounts of DABCO (10–100 mol %) through ^{1}H NMR analysis of reaction media regarding the yields of residual aldehyde, residual aryl bromide, product and byproducts. Reaction side products include the respective ester and some dehalogenated substances such as benzene and benzonitrile.

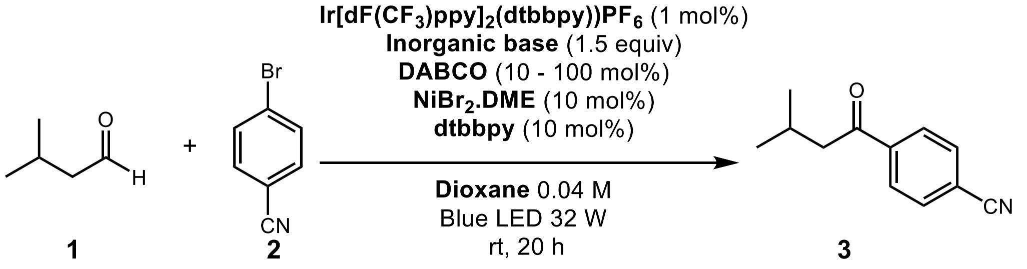

Entry	Inorganic base	DABCO (% mol)	Yield (3)[a]	Aldehyde (1)[b]	Aryl Bromide (2)[c]	Byproducts[d]
1	K_2CO_3	10%	4%	80%	88%	8%
2		50%	66%	3%	22%	12%
3		100%	3%	76%	93%	4%
4	No Base	50%	61%	27%	21%	18%
5		70%	69%	22%	3%	28%
6		100%	69%	15%	Traces	31%
7	$NaHCO_3$	10%	55%	24%	7%	38%
8		50%	81%	6%	Traces	19%
9		100%	69%	31%	Traces	31%

Reactions performed according to the general procedure. Yields determined by ^1H NMR analysis using 1,3-benzodioxole as internal standard and the following signals for reference. [a] Yield of product determined from 8.05 ppm and 7.83 ppm signals. [b] Residual aldehyde amounts were determined using the 9.70 ppm signal. [c]Residual aryl bromide amounts were determined using the 7.55 ppm signals. [d]Byproducts of this reaction include the respective ester and dehalogenation side products. Their amounts were estimated from the difference of initial aryl bromide and the residual aryl bromide and product amounts.

The results clearly indicate that intermediate amounts of DABCO are preferred, (Entries 2, 4 and 8) rather than increased or decreased concentrations. Also, results indicate sodium hydrogen carbonate as the best base for the reaction, although good results could be achieved both using potassium carbonate or no base at all. It is remarkable that increasing DABCO concentration led to diminished yields of product when inorganic bases are present, especially when using potassium carbonate (Entry 3). No clear explanation for this phenomenon could be rationalized by our group, but we can notice that when no inorganic base is present, there is no deterioration in yield with the increase in DABCO concentration (Entry 6). When potassium carbonate is present (and in a minor degree when sodium hydrogen carbonate is used), we can see that increasing DABCO loading leads to an increase in aldehyde residual amounts (Entries 3 and 9), indicating that high concentrations of this amine can undermine not only our desired mechanism but also parallel photocatalytic mechanisms that can be leading to aldehyde degradation. A more detailed study of this phenomenon is underway.

Table S2: Time Study. The reactions were carried out according to the general procedure outlined in section 3 with with variation of reaction time.

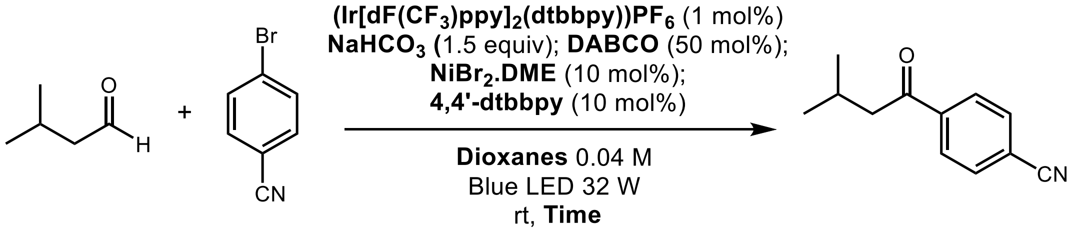

Entry	Time (h)	Yield (%)[a]
1	5	40%
2	10	55%
3	20	81%

[a]Yield determined after product isolation

Investigation of aryl aldehydes reaction conditions

Table S3: Aryl aldehyde reaction investigation. Reactions were conducted according to the general procedure with modifications as stated in the table below.

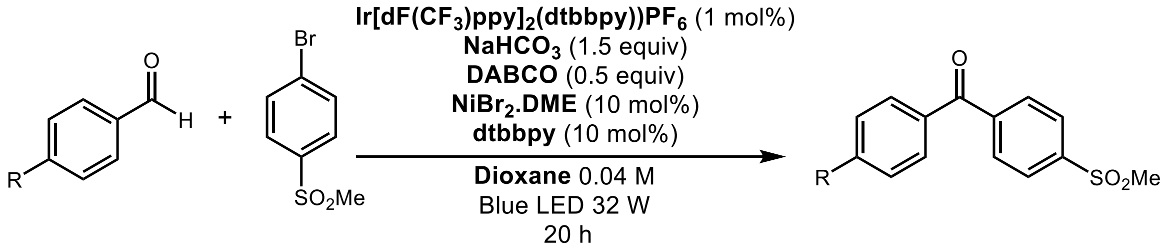

Entry	Aldehyde	Aldehyde (equiv)	Temperature	Yield
1	R = H	2 equiv	40°C	10%[a]
2	R = H	10 equiv	rt	51%[a]
3	R = H	10 equiv	40°C	70%
4	R = OMe	10 equiv	rt	55%
5	R = OMe	10 equiv	40°C	23%[a]

[a]Yield determined by ^1H NMR using 1,3-benzodioxole as internal standard.
Reactions performed at room temperature used cooling fan, and reactions performed at 40°C were heated only by turning the cooling fan off.

The use of the standard 2 equivalents of aldehyde with reaction heating led to a very poor yield when using benzaldehyde. (Entry 1) We had an improvement in yield when using 10 equivalents, but it was still necessary to heat the reaction to 40 °C to obtain good

yields. (Entries 2 and 3). Surprisingly, heating the reaction caused the reverse effect when 4-anisaldehyde was used, so the best results were achieved using a cooling fan.

3) General procedure for aldehyde arylation

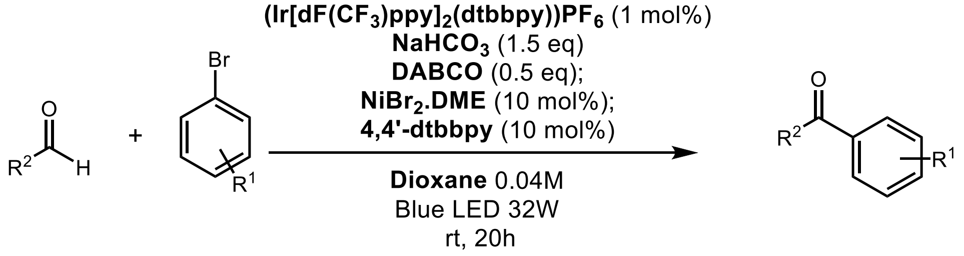

To a 5mL glass vial were added $NiBr_2$.DME (3.7 mg, 0.012 mmol, 0.11 equiv), 4,4'-di-*tert*-butyl-2,2'-bipyridine (dtbbpy, 3.2 mg, 0.012 mmol, 0.11 equiv) as ligand and 3 mL of dry 1,4-dioxane. This precatalyst vial was sealed and sonicated for around 15 minutes, until its content became homogeneous.

A separated 5 mL vial equipped with a magnetic stir bar was charged with the photocatalyst $Ir[dF(CF_3)ppy]_2(dtbbpy)PF_6$ (1.2 mg, 0.0011 mmol, 1 mol %), 1,4-diazabicyclo[2.2.2]octane (DABCO, 6.2 mg, 0.055 mmol, 0.5 equiv), $NaHCO_3$ (13.9 mg, 0.16 mmol, 1.5 equiv), the aryl bromide (0.11 mmol, 1 equiv) and the aldehyde (0.22 mmol, 2 equiv). The volatile aldehydes were added after the sparging. The precatalyst was syringed into the reaction vial before sparging with argon for 15 minutes. The vial was sealed with parafilm and the reaction was magnetically stirred and irradiated for 20 h with a blue LED lamp 6 cm away from the vial with a cooling fan to keep the reaction at room temperature. After that time, the reaction was quenched by exposure to air and filtered through a pad of silica with ethyl acetate and dichloromethane. After concentration, the residue was purified by flash chromatography on silica gel to afford the desired product.

The isolated yields reported are the average yield of four simultaneous and identical reactions.

4) Aryl bromide scope

4-(3-Methylbutanoyl)benzonitrile (3)

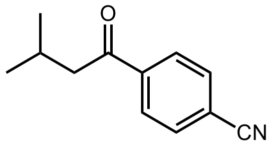

Prepared following the general procedure outlined in section 3 using $NiBr_2$.DME (3.7 mg, 0.012 mmol, 0.11 equiv), 4,4'-di-*tert*-butyl-2,2'-bipyridine (dtbbpy, 3.2 mg, 0.012 mmol, 0.11 equiv), $Ir[dF(CF_3)ppy]_2(dtbbpy)PF_6$ (1.2 mg, 0.0011 mmol, 1 mol %), 1,4-diazabicyclo[2.2.2]octane (DABCO, 6.2 mg, 0.055 mmol, 0.5 equiv), $NaHCO_3$ (13.9 mg, 0.16 mmol, 1.5 equiv), 4-bromobenzonitrile (20 mg, 0.11 mmol, 1 equiv), isovaleraldehyde (23 µL, 0.22 mmol, 2 equiv) and 1,4-dioxane (3 mL). Purification by flash chromatography (10% ethyl acetate in hexanes) provided the compound (66.7 mg, 81% – mean yield for four runs) as a white solid. Spectral data matches previous literature.[1] **^{1}H NMR (400 MHz, $CDCl_3$)** δ 8.02 (d, *J* = 8.5 Hz, 2H), 7.76 (d, *J* = 8.5 Hz, 2H), 2.84 (d, *J* = 6.8 Hz, 2H), 2.35 – 2.22 (m, 1H), 1.00 (d, *J* = 6.7 Hz, 6H).

^{13}C NMR (101 MHz, CDCl3) δ 198.8, 140.3, 132.6, 128.6, 118.1, 116.3, 47.8, 25.0, 22,8.

1-(4-Methoxyphenyl)-3-methylbutan-1-one (4)

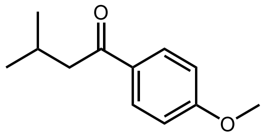

Prepared following the general procedure outlined in section 3 using $NiBr_2$.DME (3.7 mg, 0.012 mmol, 0.11 equiv), 4,4'-di-*tert*-butyl-2,2'-bipyridine (dtbbpy, 3.2 mg, 0.012 mmol, 0.11 equiv), $Ir[dF(CF_3)ppy]_2(dtbbpy)PF_6$ (1.2 mg, 0.0011 mmol, 1 mol %), 1,4-diazabicyclo[2.2.2]octane (DABCO, 6.2 mg, 0.055 mmol, 0.5 equiv), $NaHCO_3$ (13.9 mg, 0.16 mmol, 1.5 equiv), 4-bromoanisole (14 µL, 0.11 mmol, 1 equiv), isovaleraldehyde (23 µL, 0.22 mmol, 2 equiv) and 1,4-dioxane (3 mL). Purification by flash chromatography (10% ethyl acetate in hexanes) provided the compound (35.5 mg, 42% yield – mean yield for four runs) as a white solid. Spectral data matches previous literature.[1][2]

^{1}H NMR (400 MHz, $CDCl_3$) δ 7.87 (d, *J* = 9.0 Hz, 2H), 6.86 (d, *J* = 9.0 Hz, 2H), 3.80 (s, 3H), 2.71 (d, *J* = 6.9 Hz, 2H), 2.27 – 2.15 (m, 1H), 0.92 (d, *J* = 6.7 Hz, 6H).

^{13}C NMR (101 MHz, CDCl3) δ 199.1, 163.4, 130.7, 130.5, 113.8, 55.6, 47.3, 25.5, 23.0.

3-Methyl-1-(p-tolyl)butan-1-one (5)

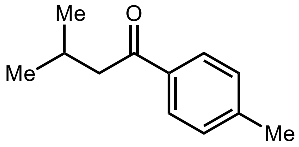

Prepared following the general procedure outlined in section 3 using $NiBr_2$.DME (3.7 mg, 0.012 mmol, 0.11 equiv), 4,4'-di-*tert*-butyl-2,2'-bipyridine (dtbbpy, 3.2 mg, 0.012 mmol, 0.11 equiv), $Ir[dF(CF_3)ppy]_2(dtbbpy)PF_6$ (1.2 mg, 0.0011 mmol, 1 mol %), 1,4-diazabicyclo[2.2.2]octane (DABCO, 6.2 mg, 0.055 mmol, 0.5 equiv), $NaHCO_3$ (13.9 mg, 0.16 mmol, 1.5 equiv), 4-bromotoluene (13.5 μL, 0.11 mmol, 1 equiv), isovaleraldehyde (23 μL, 0.22 mmol, 2 equiv) and 1,4-dioxane (3 mL). Purification by flash chromatography (10% ethyl acetate in hexanes) provided the compound (41.1 mg, 53% yield – mean yield for four runs) as a white solid.Spectral data matches previous literature.[1][3]

^{1}H NMR (400 MHz, $CDCl_3$) δ 7.78 (d, *J* = 8.2 Hz, 2H), 7.18 (d, *J* = 8.3 Hz, 2H), 2.73 (d, *J* = 6.9 Hz, 2H), 2.33 (s, 3H), 2.26 – 2.18 (m, 1H), 0.91 (d, *J* = 6.7 Hz, 6H).

^{13}C NMR (126 MHz, $CDCl_3$) δ 200.1, 143.7, 135.1, 129.3, 128.4, 47.6, 25.4, 23.0, 21.7.

3-Methyl-1-phenylbutan-1-one (6)

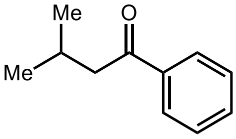

Prepared following the general procedure outlined in section 3 using $NiBr_2$.DME (3.7 mg, 0.012 mmol, 0.11 equiv), 4,4'-di-*tert*-butyl-2,2'-bipyridine (dtbbpy, 3.2 mg, 0.012 mmol, 0.11 equiv), $Ir[dF(CF_3)ppy]_2(dtbbpy)PF_6$ (1.2 mg, 0.0011 mmol, 1 mol %), 1,4-diazabicyclo[2.2.2]octane (DABCO, 6.2 mg, 0.055 mmol, 0.5 equiv), $NaHCO_3$ (13.9 mg, 0.16 mmol, 1.5 equiv), bromobenzene (12 μL, 0.11 mmol, 1 equiv), isovaleraldehyde (23 μL, 0.22 mmol, 2 equiv) and 1,4-dioxane (3 mL). Purification by flash chromatography (10% ethyl acetate in hexanes) provided the compound (27.8 mg, 39% yield – mean yield for four runs) as a white solid. Spectral data matches previous literature.[1][4]

^{1}H NMR (500 MHz, $CDCl_3$) δ 7.95 (d, *J* = 7.6 Hz, 2H), 7.55 (t, *J* = 7.3 Hz, 1H), 7.46 (t, *J* = 7.6 Hz, 2H), 2.84 (d, *J* = 6.9 Hz, 2H), 2.30 (dt, *J* = 13.4, 6.7 Hz, 1H), 1.00 (d, J = 6.7 Hz, 6H).

^{13}C NMR (126 MHz, $CDCl_3$) δ 200.5, 137.5, 133.0, 128.7, 128.2, 47.7, 25.3, 22.9.

3-Methyl-1-(4-(trifluoromethyl)phenyl)butan-1-one (7)

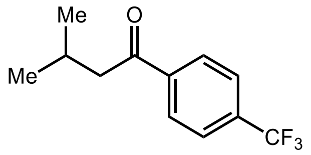

Prepared following the general procedure outlined in section 3 using $NiBr_2$.DME (3.7 mg, 0.012 mmol, 0.11 equiv), 4,4'-di-*tert*-butyl-2,2'-bipyridine (dtbbpy, 3.2 mg, 0.012 mmol, 0.11 equiv), Ir[dF(CF_3)ppy]$_2$(dtbbpy)PF_6 (1.2 mg, 0.0011 mmol, 1 mol %), 1,4-diazabicyclo[2.2.2]octane (DABCO, 6.2 mg, 0.055 mmol, 0.5 equiv), $NaHCO_3$ (13.9 mg, 0.16 mmol, 1.5 equiv), 4-bromobenzotrifluoride (15.4 μL, 0.11 mmol, 1 equiv), isovaleraldehyde (23 μL, 0.22 mmol, 2 equiv) and 1,4-dioxane (3 mL). Purification by flash chromatography (30% ethyl acetate in hexanes) provided the compound (65.8 mg, 65% yield - mean yield for four runs) as a white solid. Spectral data matches previous literature.[5]

^{1}H NMR (500 MHz, $CDCl_3$) δ 7.96 (d, *J* = 8.3 Hz, 2H), 7.64 (d, *J* = 8.3 Hz, 2H), 2.78 (d, *J* = 6.9 Hz, 2H), 2.26 – 2.18 (m, 1H), 0.92 (d, *J* = 6.7 Hz, 6H).

^{13}C NMR (126 MHz, $CDCl_3$) δ 199.3, 140.2, 134.3 (q, *J* = 32.7 Hz), 128.6, 125.8 (q, *J* = 3.8 Hz), 123.8 (q, *J* = 272.7 Hz), 47.9, 25.2, 22.8.

1-(4-Acetylphenyl)-3-methylbutan-1-one (8)

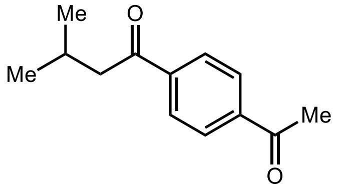

Prepared following the general procedure outlined in section 3 using $NiBr_2$.DME (3.7 mg, 0.012 mmol, 0.11 equiv), 4,4'-di-*tert*-butyl-2,2'-bipyridine (dtbbpy, 3.2 mg, 0.012 mmol, 0.11 equiv), Ir[dF(CF3)ppy]2(dtbbpy)PF6 (1.2 mg, 0.0011 mmol, 1 mol %), 1,4-diazabicyclo[2.2.2]octane (DABCO, 6.2 mg, 0.055 mmol, 0.5 equiv), $NaHCO_3$ (13.9 mg, 0.16 mmol, 1.5 equiv), 4'-bromoacetophenone (21.9 mg, 0.11 mmol, 1 equiv), isovaleraldehyde (23 μL, 0.22 mmol, 2 equiv) and 1,4-dioxane (3 mL). Purification by flash chromatography (30% ethyl acetate in hexanes) provided the compound (81.8 mg, 91% yield - mean yield for four runs) as a white solid. Spectral data matches previous literature.[6]

^{1}H NMR (500 MHz, $CDCl_3$) δ 8.04 – 8.00 (m, 4H), 2.86 (d, *J* = 6.9 Hz, 2H), 2.64 (s, 3H), 2.34 – 2.25 (m, 1H), 1.00 (d, *J* = 6.7 Hz, 6H).

^{13}C NMR (126 MHz, CDCl$_3$) δ 199.8, 197.6, 140.6, 140.1, 128.6, 128.4, 47.9, 27.0, 25.2, 22.8.

3-Methyl-1-(4-(methylsulfonyl)phenyl)butan-1-one (9)

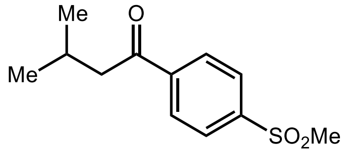

Prepared following the general procedure outlined in section 3 using $NiBr_2$.DME (3.7 mg, 0.012 mmol, 0.11 equiv), 4,4'-di-*tert*-butyl-2,2'-bipyridine (dtbbpy, 3.2 mg, 0.012 mmol, 0.11 equiv), Ir[dF(CF3)ppy]2(dtbbpy)PF6 (1.2 mg, 0.0011 mmol, 1 mol %), 1,4-diazabicyclo[2.2.2]octane (DABCO, 6.2 mg, 0.055 mmol, 0.5 equiv), $NaHCO_3$ (13.9 mg, 0.16 mmol, 1.5 equiv), 4-bromophenyl methyl sulfone (25.9 mg, 0.11 mmol, 1 equiv), isovaleraldehyde (23 μL, 0.22 mmol, 2 equiv) and 1,4-dioxane (3 mL). Purification by flash chromatography (30% ethyl acetate in hexanes) provided the compound (93.0 mg, 88% yield - mean yield for four runs) as a white solid. Spectral data matches previous literature.[7]

^{1}H NMR (500 MHz, CDCl$_3$) δ 8.12 (d, *J* = 8.5 Hz, 2H), 8.05 (d, *J* = 8.5 Hz, 2H), 3.09 (s, 3H), 2.88 (d, *J* = 6.9 Hz, 2H), 2.35 – 2.25 (m, 1H), 1.01 (d, *J* = 6.7 Hz, 6H).

^{13}C NMR (126 MHz, CDCl$_3$) δ 199.0, 144.0, 141.4, 129.0, 127.8, 48.0, 44.4, 25.1, 22.7

1-(5-Fluoro-2-methylphenyl)-3-methylbutan-1-one (10)

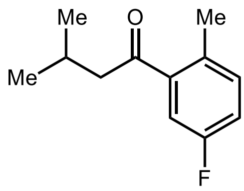

Prepared following the general procedure outlined in section 3 using $NiBr_2$.DME (3.7 mg, 0.012 mmol, 0.11equiv), 4,4'-di-*tert*-butyl-2,2'-bipyridine (dtbbpy, 3.2 mg, 0.012 mmol, 0.11 equiv), Ir[dF(CF3)ppy]2(dtbbpy)PF6 (1.2 mg, 0.0011 mmol, 1 mol %), 1,4-diazabicyclo[2.2.2]octane (DABCO, 6.2 mg, 0.055 mmol, 0.5 equiv), $NaHCO_3$ (13.9 mg, 0.16 mmol, 1.5 equiv), 2-bromo-4-fluorotoluene (20.8 mg, 0.11 mmol, 1 equiv), isovaleraldehyde (23 μL, 0.22 mmol, 2 equiv) and 1,4-dioxane (3 mL). Purification by flash chromatography (30% ethyl acetate in hexanes) provided the compound (62.4 mg, 73% yield - mean yield for four runs) as a yellow oil.

^{1}H NMR (500 MHz, CDCl$_3$) δ 7.25 (dd, *J* = 9.3, 2.7 Hz, 1H), 7.19 (dd, *J* = 8.4, 5.6 Hz, 1H), 7.05 (td, *J* = 8.3, 2.7 Hz, 1H), 2.72 (d, J = 6.9 Hz, 2H), 2.43 (s, 3H), 2.28 – 2.20 (m, 1H), 0.98 (d, *J* = 6.7 Hz, 6H).

^{13}C NMR (126 MHz, CDCl$_3$) δ 203.6 (d, *J* = 2.0 Hz), 160.7 (d, *J* = 245.2 Hz), 140.0 (d, *J* = 5.3 Hz), 133.4 (d, *J* = 7.2 Hz), 133.2 (d, *J* = 3.4 Hz), 117.8 (d, *J* = 20.6 Hz), 115.0 (d, *J* = 22.1 Hz), 50.7, 25.2, 22.7, 20.3.

3-Methyl-1-(6-(trifluoromethyl)pyridin-3-yl)butan-1-one (11)

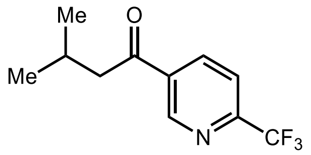

Prepared following the general procedure outlined in section 3 using $NiBr_2$.DME (3.7 mg, 0.012 mmol, 0.11 equiv), 4,4'-di-*tert*-butyl-2,2'-bipyridine (dtbbpy, 3.2 mg, 0.012 mmol, 0.11 equiv), Ir[dF(CF3)ppy]2(dtbbpy)PF6 (1.2 mg, 0.0011 mmol, 1 mol %), 1,4-diazabicyclo[2.2.2]octane (DABCO, 6.2 mg, 0.055 mmol, 0.5 equiv), $NaHCO_3$ (13.9 mg, 0.16 mmol, 1.5 equiv), 5-bromo-2-(trifluoromethyl)pyridine (24.9 mg, 0.11 mmol, 1 equiv), isovaleraldehyde (23 µL, 0.22 mmol, 2 equiv) and 1,4-dioxane (3 mL). Purification by flash chromatography (30% ethyl acetate in hexanes) provided the compound (75.3 mg, 74% yield - mean yield for four runs) as a white solid.

Mp 38-40 °C

^{1}H NMR (400 MHz, CDCl$_3$) δ 9.22 (s, 1H), 8.39 (d, *J* = 8.2 Hz, 1H), 7.80 (d, *J* = 8.1 Hz, 1H), 2.88 (d, *J* = 6.8 Hz, 2H), 2.37 – 2.26 (m, 1H), 1.02 (d, *J* = 6.7 Hz, 6H).

^{13}C NMR (126 MHz, CDCl$_3$) δ 197.8, 151.1 (q, *J* = 35.1 Hz), 149.7, 137.1, 134.5, 121.2 (q, *J* = 274.5 Hz), 120.6 (q, *J* = 2.7 Hz), 48.1, 25.0, 22.7.

3-Methyl-1-(pyrimidin-5-yl)butan-1-one (12)

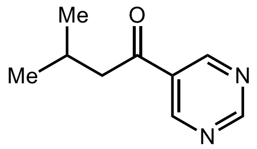

Prepared following the general procedure outlined in section 3 using $NiBr_2$.DME (3.7 mg, 0.012 mmol, 0.11 equiv), 4,4'-di-*tert*-butyl-2,2'-bipyridine (dtbbpy, 3.2 mg, 0.012 mmol, 0.11 equiv), Ir[dF(CF3)ppy]2(dtbbpy)PF6 (1.2 mg, 0.0011 mmol, 1 mol %), 1,4-diazabicyclo[2.2.2]octane (DABCO, 6.2 mg, 0.055 mmol, 0.5 equiv), $NaHCO_3$ (13.9 mg, 0.16 mmol, 1.5 equiv), 5-bromopyrimidine (17.5 mg, 0.11 mmol, 1 equiv), isovaleraldehyde (23 µL, 0.22 mmol, 2 equiv) and 1,4-dioxane (3 mL). Purification by flash chromatography (30% ethyl acetate in hexanes) provided the compound (52.7 mg, 73% yield - mean yield for four runs) as a yellow oil.

^{1}H NMR (500 MHz, CDCl$_3$) δ 9.33 (s, 1H), 9.20 (s, 2H), 2.83 (d, *J* = 6.8 Hz, 2H), 2.33 – 2.25 (m, 1H), 1.00 (d, *J* = 6.7 Hz, 6H).

^{13}C NMR (126 MHz, CDCl$_3$) δ 197.5, 161.3, 156.7, 129.9, 48.0, 24.9, 22.7.

5) Aldehyde scope

1-(4-(Methylsulfonyl)phenyl)hexan-1-one (13)

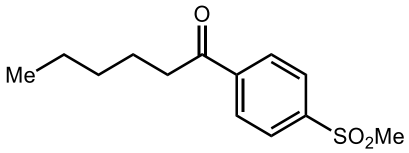

Prepared following the general procedure outlined in section 3 using NiBr$_2$.DME (3.7 mg, 0.012 mmol, 0.11 equiv), 4,4'-di-*tert*-butyl-2,2'-bipyridine (dtbbpy, 3.2 mg, 0.012 mmol, 0.11 equiv), Ir[dF(CF3)ppy]2(dtbbpy)PF6 (1.2 mg, 0.0011 mmol, 1 mol %), 1,4-diazabicyclo[2.2.2]octane (DABCO, 6.2 mg, 0.055 mmol, 0.5 equiv), NaHCO$_3$ (13.9 mg, 0.16 mmol, 1.5 equiv), 4-bromophenyl methyl sulfone (25.9 mg, 0.11 mmol, 1 equiv), hexanal (27 μL, 0.22 mmol, 2 equiv) and 1,4-dioxane (3 mL). Purification by flash chromatography (30% ethyl acetate in hexanes) provided the compound (100.0 mg, 89% yield - mean yield for four runs) as a white solid.

Mp 101-103 °C

^{1}H NMR (500 MHz, CDCl$_3$) δ 8.14 – 8.10 (m, 2H), 8.06 – 8.02 (m, 2H), 3.08 (s, 3H), 3.00 (t, *J* = 7.4 Hz, 2H), 1.80 – 1.70 (m, 2H), 1.39 – 1.35 (m, 4H), 0.96 – 0.86 (m, 3H).

^{13}C NMR (126 MHz, CDCl$_3$) δ 199.3, 144.0, 141.0, 128.9, 127.8, 44.3, 39.1, 31.4, 23.7, 22.5, 14.0.

1-(4-(Methylsulfonyl)phenyl)-3-phenylpropan-1-one (14)

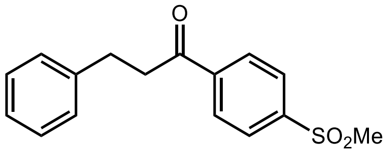

Prepared following the general procedure outlined in section 3 using NiBr$_2$.DME (3.7 mg, 0.012 mmol, 0.11 equiv), 4,4'-di-*tert*-butyl-2,2'-bipyridine (dtbbpy, 3.2 mg, 0.012 mmol, 0.11 equiv), Ir[dF(CF3)ppy]2(dtbbpy)PF6 (1.2 mg, 0.0011 mmol, 1 mol %), 1,4-diazabicyclo[2.2.2]octane (DABCO, 6.2 mg, 0.055 mmol, 0.5 equiv), NaHCO$_3$ (13.9 mg, 0.16 mmol, 1.5 equiv), 4-bromophenyl methyl sulfone (25.9 mg, 0.11 mmol, 1 equiv), hydrocinnamaldehyde (29 μL, 0.22 mmol, 2 equiv) and 1,4-dioxane (3 mL).

Purification by flash chromatography (30% ethyl acetate in hexanes) provided the compound (101.5 mg, 80% yield - mean yield for four runs) as a white solid. Spectral data matches previous literature.[7]

^{1}H NMR (500 MHz, CDCl$_3$) δ 8.13 – 8.09 (m, 2H), 8.05 – 8.00 (m, 2H), 7.34 – 7.27 (m, 2H), 7.27 – 7.18 (m, 3H), 3.34 (t, *J* = 7.6 Hz, 2H), 3.11 – 3.05 (m, 5H).

^{13}C NMR (126 MHz, CDCl$_3$) δ 198.0, 144.2, 140.8, 140.8, 129.0, 128.7, 128.5, 127.9, 126.4, 44.4, 41.0, 30.0

***tert*-Butyl 4-(4-(methylsulfonyl)benzoyl)piperidine-1-carboxylate (15)**

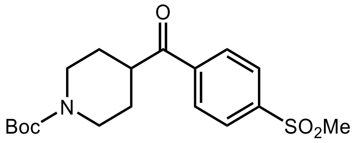

Prepared following the general procedure outlined in section 3 using NiBr$_2$.DME (3.7 mg, 0.012 mmol, 0.11 equiv), 4,4'-di-*tert*-butyl-2,2'-bipyridine (dtbbpy, 3.2 mg, 0.012 mmol, 0.11 equiv), Ir[dF(CF3)ppy]2(dtbbpy)PF6 (1.2 mg, 0.0011 mmol, 1 mol %), 1,4-diazabicyclo[2.2.2]octane (DABCO, 6.2 mg, 0.055 mmol, 0.5 equiv), NaHCO$_3$ (13.9 mg, 0.16 mmol, 1.5 equiv), 4-Bromophenyl methyl sulfone (25.9 mg, 0.11 mmol, 1 equiv), *N*-Boc-4-piperidinecarboxaldehyde (47 mg, 0.22 mmol, 2 equiv) and 1,4-dioxane (3 mL). Purification by flash chromatography (30% ethyl acetate in hexanes) provided the compound (40.4 mg, 69% yield - mean yield for four runs) as a white solid. Spectral data matches previous literature.[8]

^{1}H NMR (500 MHz, CDCl$_3$) δ 8.08 (d, *J* = 8.6 Hz, 2H), 8.04 (d, *J* = 8.5 Hz, 2H), 4.14 (brs, 2H), 3.39 (tt, *J* = 11.1, 3.7 Hz, 1H), 3.07 (s, 3H), 2.93 – 2.88 (m, 2H), 1.84 (d, *J* = 12.6 Hz, 2H), 1.72 – 1.64 (m, 2H), 1.45 (s, 9H).

^{13}C NMR (126 MHz, CDCl$_3$) δ 201.0, 154.7, 144.2, 140.1, 129.2, 128.1, 79.9, 44.4, 44.1, 28.5, 28.2.

(4-(Methylsulfonyl)phenyl)(phenyl)methanone (16)

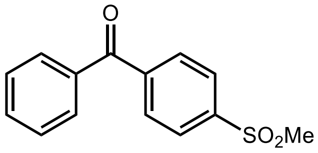

Prepared following the general procedure outlined in section 3 using NiBr$_2$.DME (3.7 mg, 0.012 mmol, 0.11 equiv), 4,4'-di-*tert*-butyl-2,2'-bipyridine (dtbbpy, 3.2 mg, 0.012 mmol, 0.11 equiv), Ir[dF(CF3)ppy]2(dtbbpy)PF6 (1.2 mg, 0.0011 mmol, 1 mol %), 1,4-diazabicyclo[2.2.2]octane (DABCO, 6.2 mg, 0.055 mmol, 0.5 equiv), NaHCO$_3$ (13.9 mg,

0.16 mmol, 1.5 equiv), 4-bromophenyl methyl sulfone (25.9 mg, 0.11 mmol, 1 equiv), benzaldehyde (112 μL, 1.1 mmol, 10 equiv) and 1,4-dioxane (3 mL). The cooling fan was turned off so the reaction proceeded at 40 °C. Purification by flash chromatography (30% ethyl acetate in hexanes) provided the compound (80.2 mg, 70% yield - mean yield for four runs) as a white solid. Spectral data matches previous literature.[9]

^{1}H NMR (500 MHz, $CDCl_3$) δ 8.09 – 8.05 (m, 2H), 7.97 – 7.92 (m, 2H), 7.79 (dt, *J* = 8.4, 1.6 Hz, 2H), 7.64 (ddd, *J* = 8.7, 2.4, 1.2 Hz, 1H), 7.54 – 7.48 (m, 2H), 3.11 (s, 3H).

^{13}C NMR (126 MHz, $CDCl_3$) δ 195.2, 143.5, 142.4, 136.4, 133.5, 130.6, 130.2, 128.7, 127.6, 44.5.

(4-Methoxyphenyl)(4-(methylsulfonyl)phenyl)methanone (17)

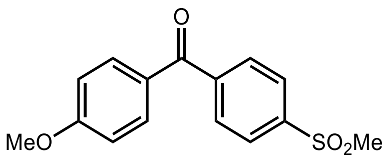

Prepared following the general procedure outlined in section 3 using $NiBr_2$.DME (3.7 mg, 0.012 mmol, 0.11 equiv), 4,4'-di-*tert*-butyl-2,2'-bipyridine (dtbbpy, 3.2 mg, 0.012 mmol, 0.11 equiv), Ir[dF(CF3)ppy]2(dtbbpy)PF6 (1.2 mg, 0.0011 mmol, 1 mol %), 1,4-diazabicyclo[2.2.2]octane (DABCO, 6.2 mg, 0.055 mmol, 0.5 equiv), $NaHCO_3$ (13.9 mg, 0.16 mmol, 1.5 equiv), 4-Bromophenyl methyl sulfone (25.9 mg, 0.11 mmol, 1 equiv), *p*-anisaldehyde (134 μL, 1.1 mmol, 10 equiv) and 1,4-dioxane (3 mL). Purification by flash chromatography (30% ethyl acetate in hexanes) provided the compound (70.2 mg, 55% yield - mean yield for four runs) as a white solid. Spectral data matches previous literature.[10]

^{1}H NMR (500 MHz, $CDCl_3$) δ 8.06 (d, *J* = 8.2 Hz, 2H), 7.89 (d, *J* = 8.2 Hz, 2H), 7.81 (d, *J* = 8.8 Hz, 2H), 6.98 (d, *J* = 8.8 Hz, 2H), 3.90 (s, 3H), 3.11 (s, 3H).

^{13}C NMR (126 MHz, $CDCl_3$) δ 194.0, 164.1, 143.4, 143.1, 132.8, 130.3, 129.1, 127.5, 114.1, 55.7, 44.5.

2,2-Dimethyl-1-(4-(methylsulfonyl)phenyl)propan-1-one (18)

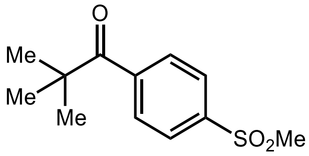

Prepared following the general procedure outlined in section 3 using $NiBr_2$.DME (3.7 mg, 0.012 mmol, 0.11 equiv), 4,4'-di-*tert*-butyl-2,2'-bipyridine (dtbbpy, 3.2 mg, 0.012 mmol, 0.11 equiv), Ir[dF(CF3)ppy]2(dtbbpy)PF6 (1.2 mg, 0.0011 mmol, 1 mol %), 1,4-diazabicyclo[2.2.2]octane (DABCO, 6.2 mg, 0.055 mmol, 0.5 equiv), $NaHCO_3$ (13.9 mg, 0.16 mmol, 1.5 equiv), 4-bromophenyl methyl sulfone (25.9 mg, 0.11 mmol, 1 equiv), pivalaldehyde (24 μL, 0.22 mmol, 2 equiv) and 1,4-dioxane (3 mL). Purification by flash chromatography (30% ethyl acetate in hexanes, then 50% dichloromethane in hexanes) provided the compound (36.0 mg, 34% yield - mean yield for four runs) as a white solid.

Mp 90-92 ºC

^{1}H NMR (500 MHz, $CDCl_3$) δ 7.99 (d, J = 8.4 Hz, 2H), 7.75 (d, J = 8.4 Hz, 2H), 3.07 (s, 3H), 1.33 (s, 9H).

^{13}C NMR (126 MHz, $CDCl_3$) δ 209.1, 144.2, 142.1, 128.3, 127.4, 44.6, 44.5, 27.7.

6) NMR Spectra

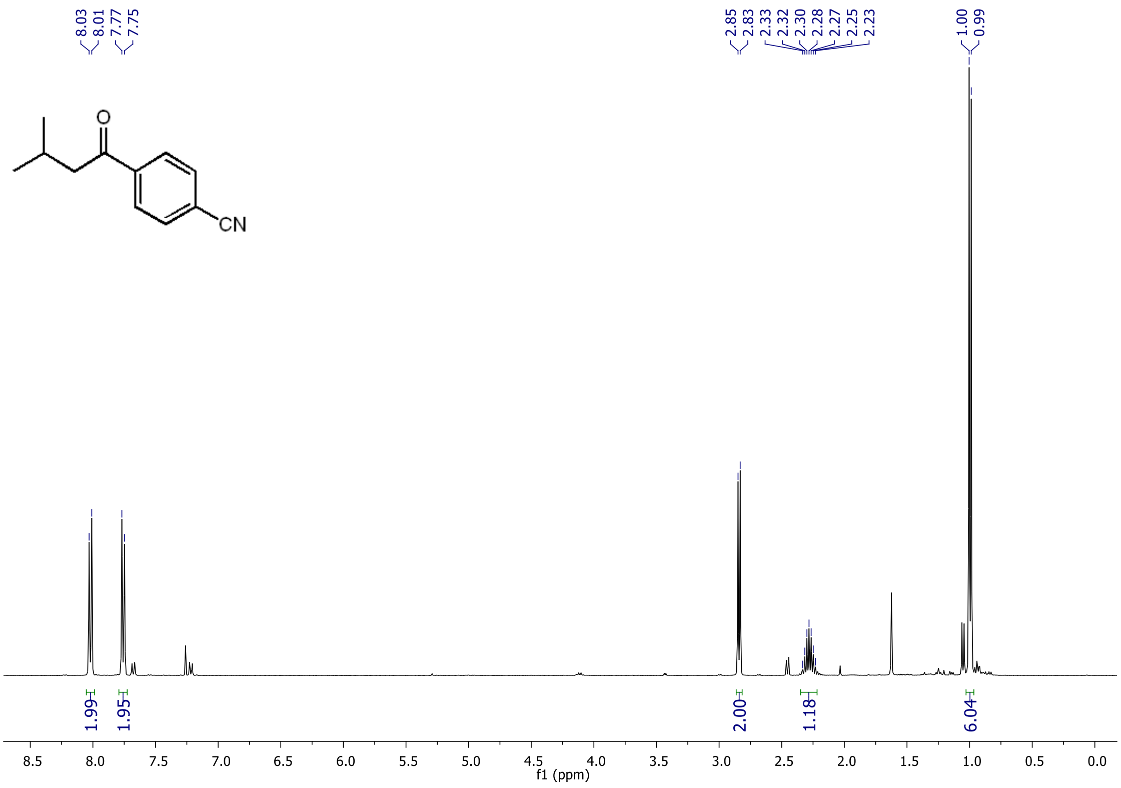

^{1}H NMR of 3 ($CDCl_3$, 400MHz)

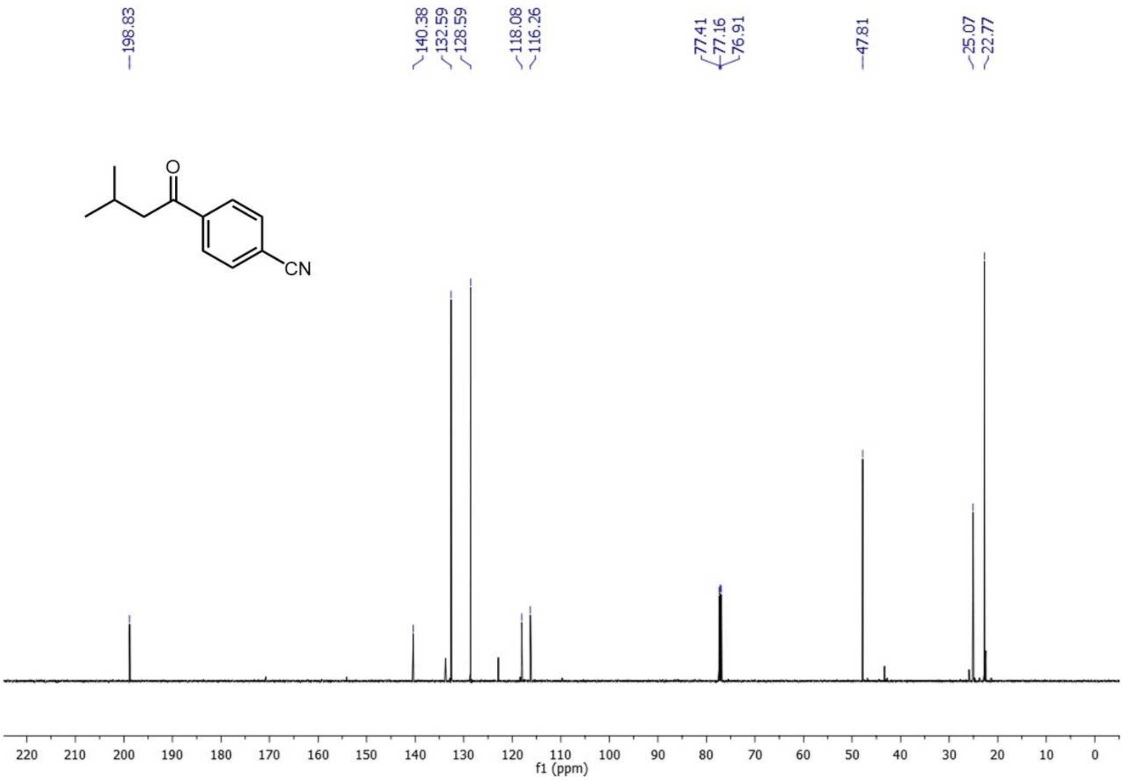

^{13}C NMR of 3 ($CDCl_3$, 126MHz)

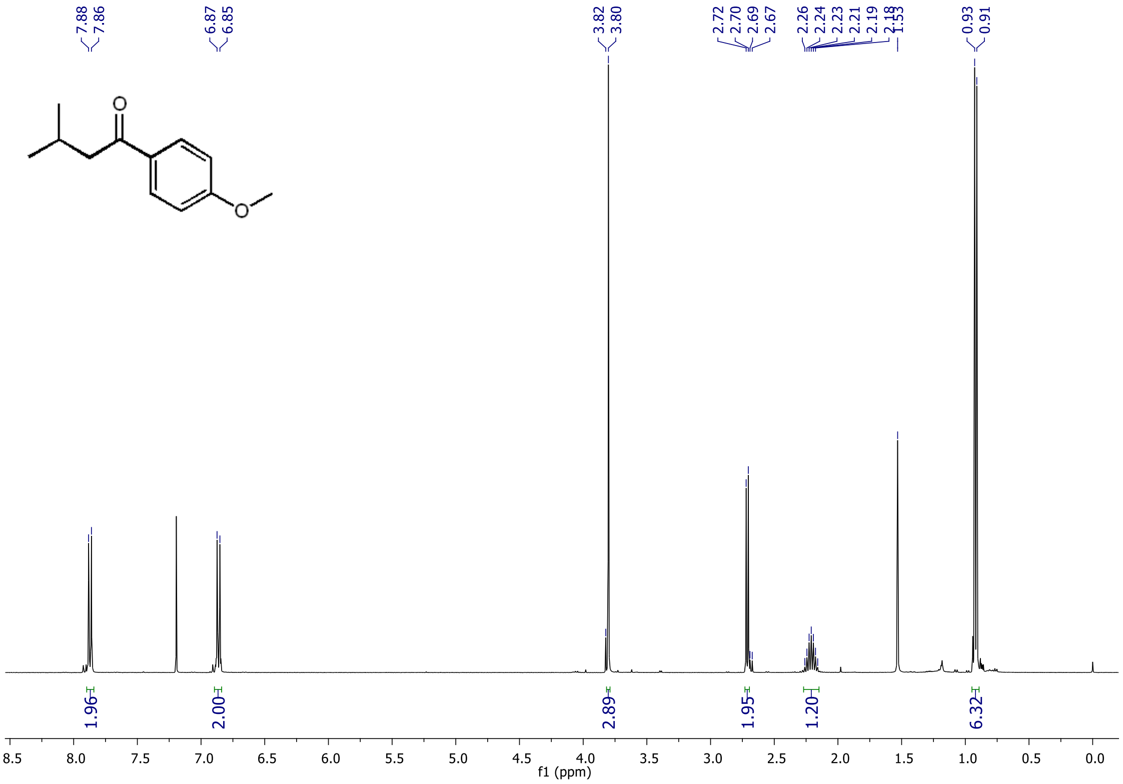

^{1}H NMR of 4 ($CDCl_3$, 400MHz)

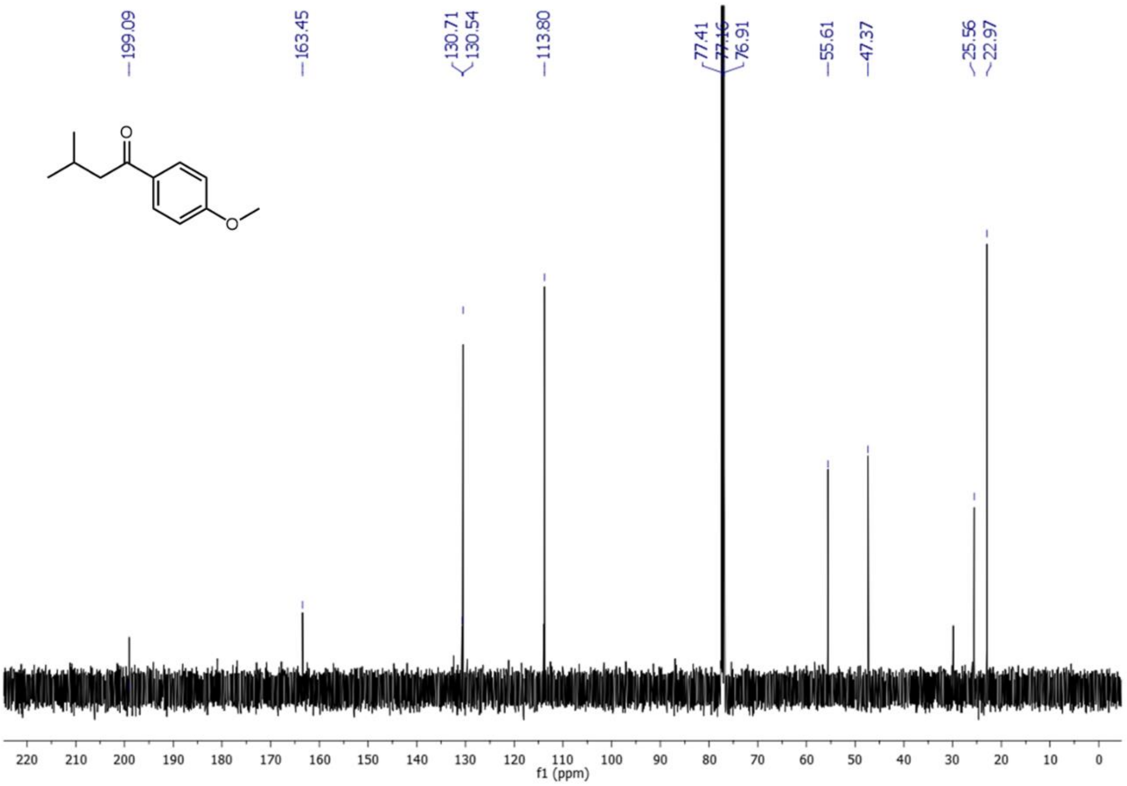

^{13}C NMR of 4 ($CDCl_3$, 101MHz)

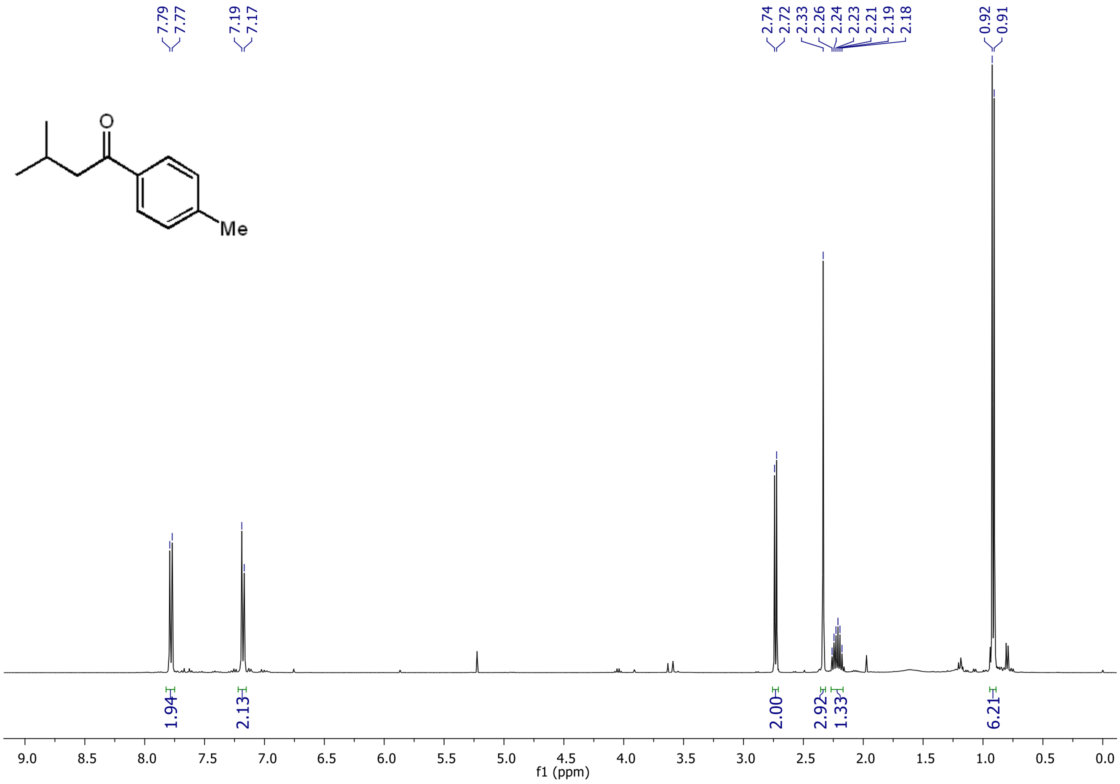

^{1}H NMR of 5 ($CDCl_3$, 400MHz)

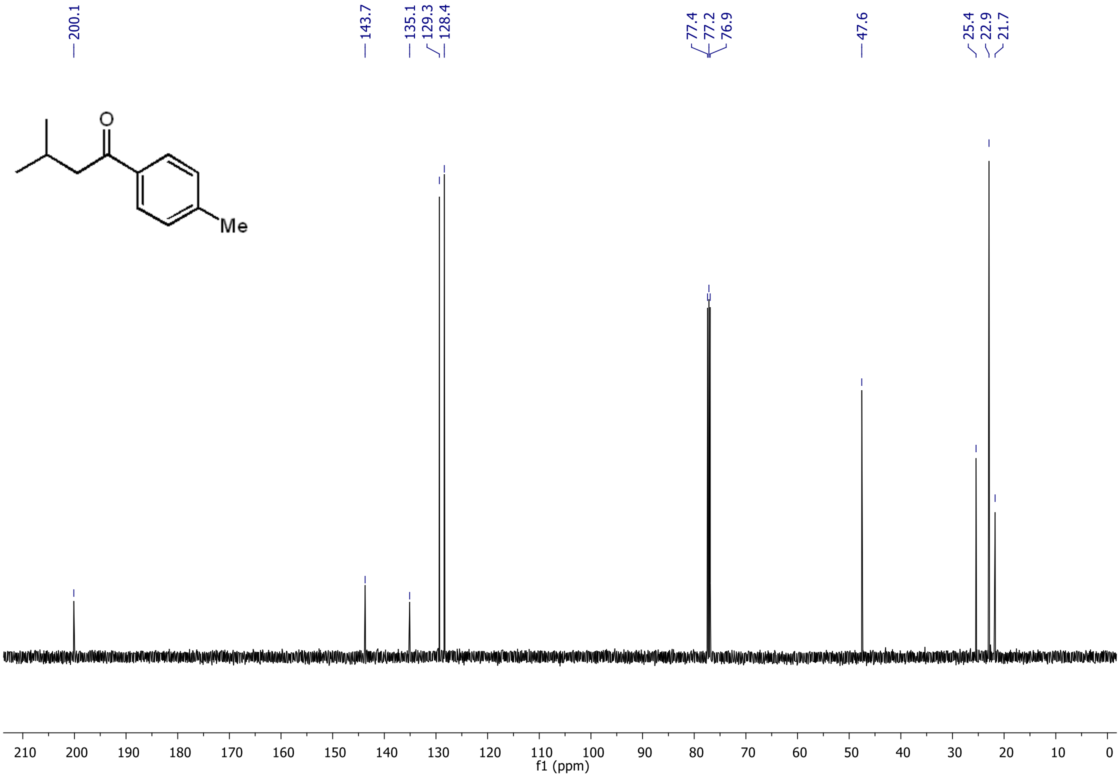

^{13}C NMR of 5 ($CDCl_3$, 126MHz)

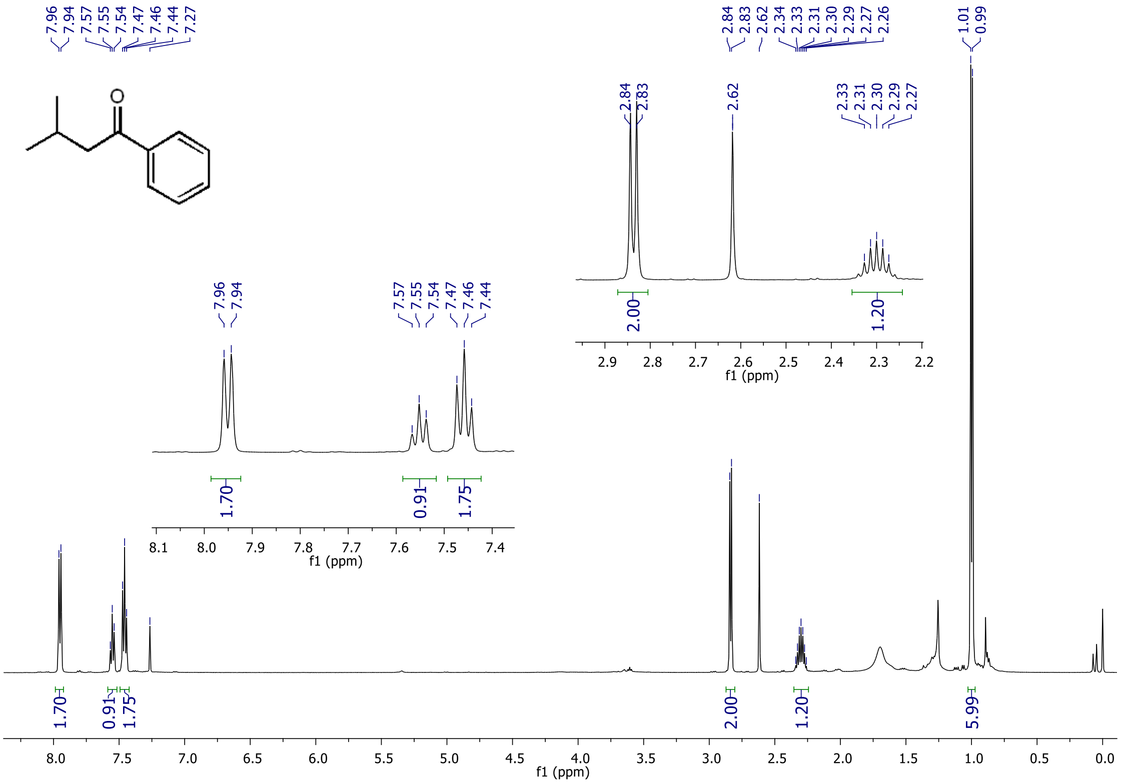

^{1}H NMR of 6 ($CDCl_3$, 500MHz)

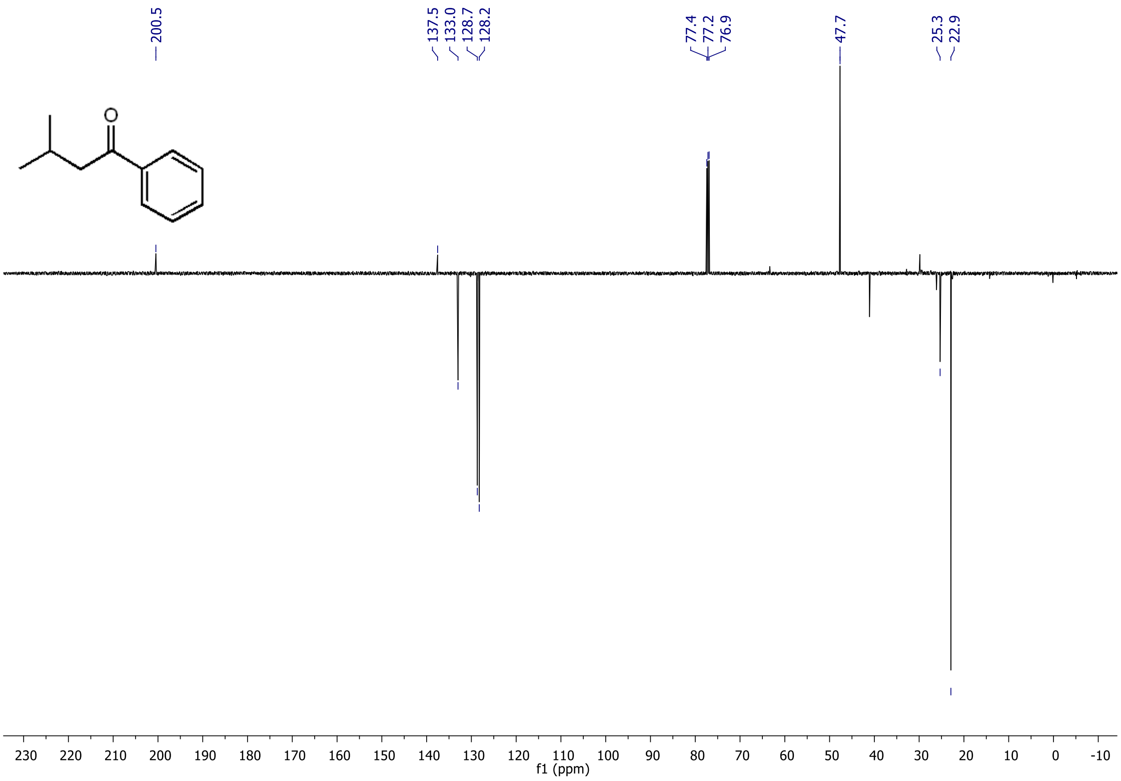

^{13}C NMR (DEPTQ) of 6 ($CDCl_3$, 126MHz)

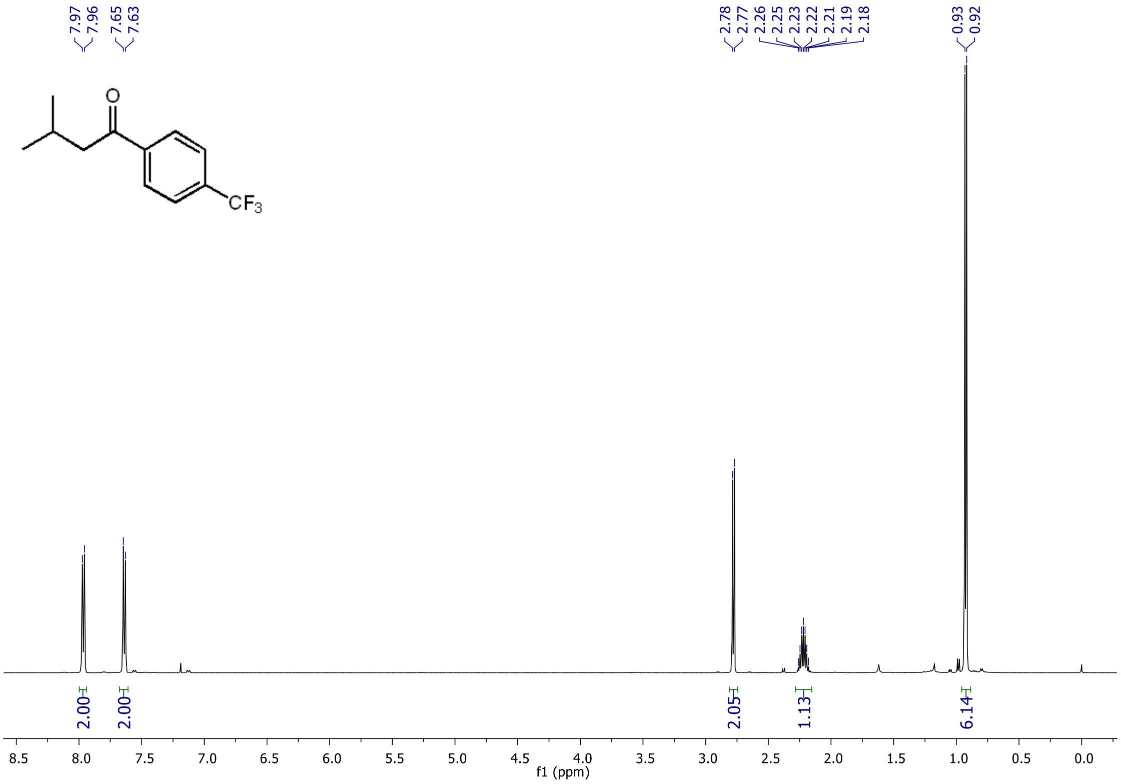

^{1}H NMR of 7 ($CDCl_3$, 500MHz)

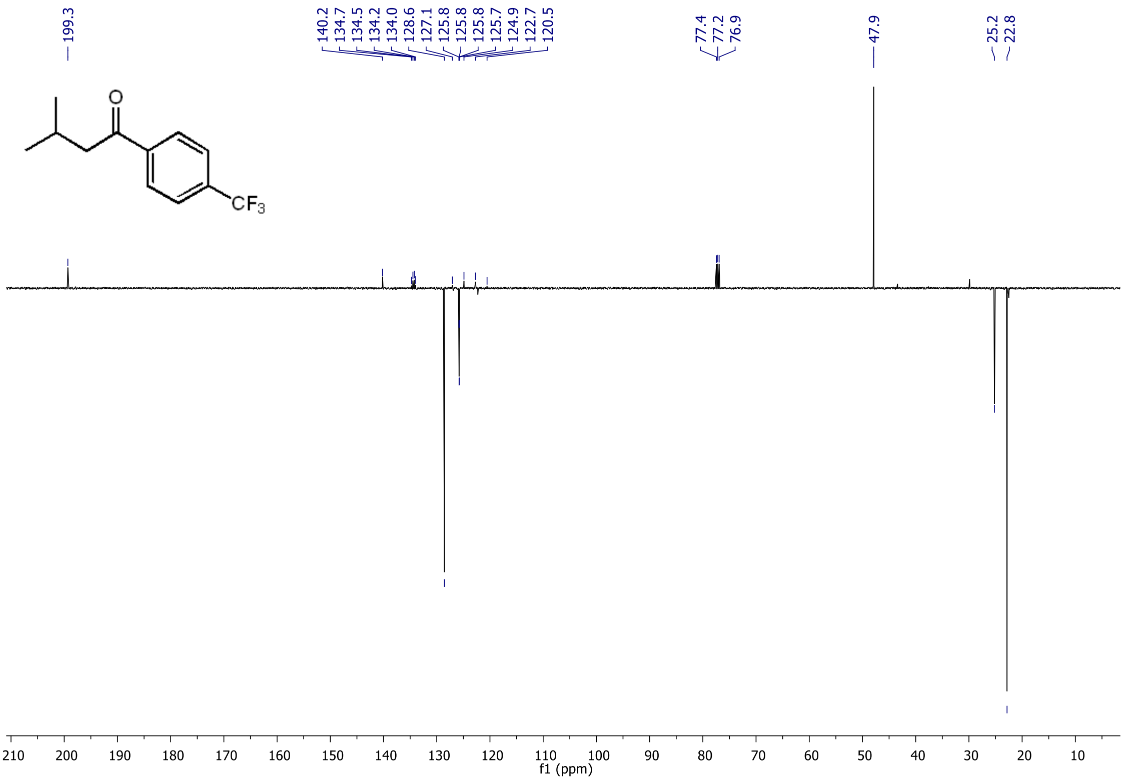

^{13}C NMR (DEPTQ) of 7 ($CDCl_3$, 126MHz)

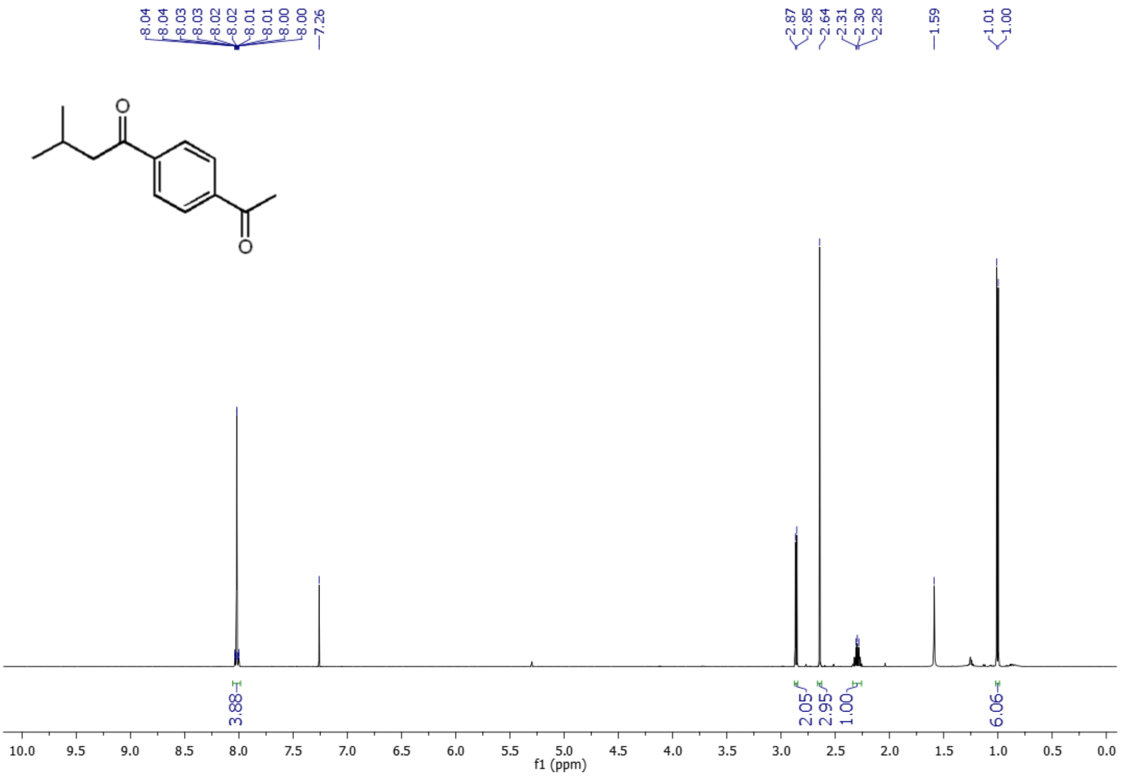

^{1}H NMR of 8 ($CDCl_3$, 500MHz)

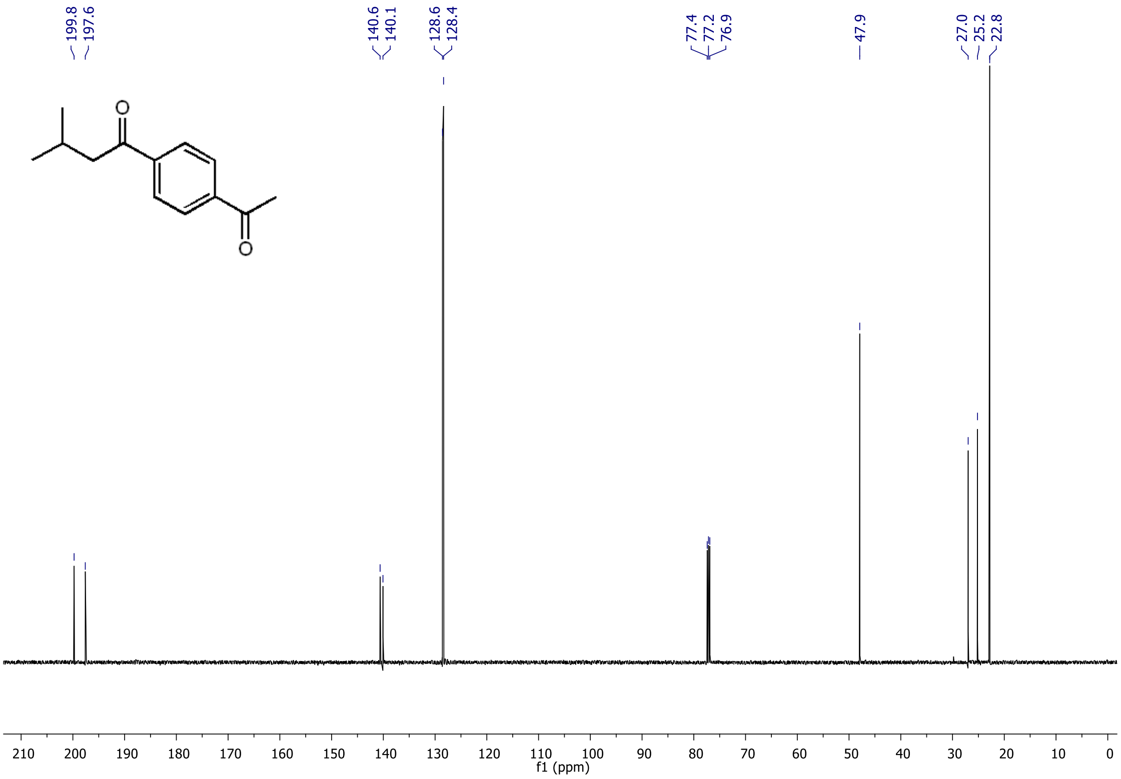

^{13}C NMR of 8 ($CDCl_3$, 126MHz)

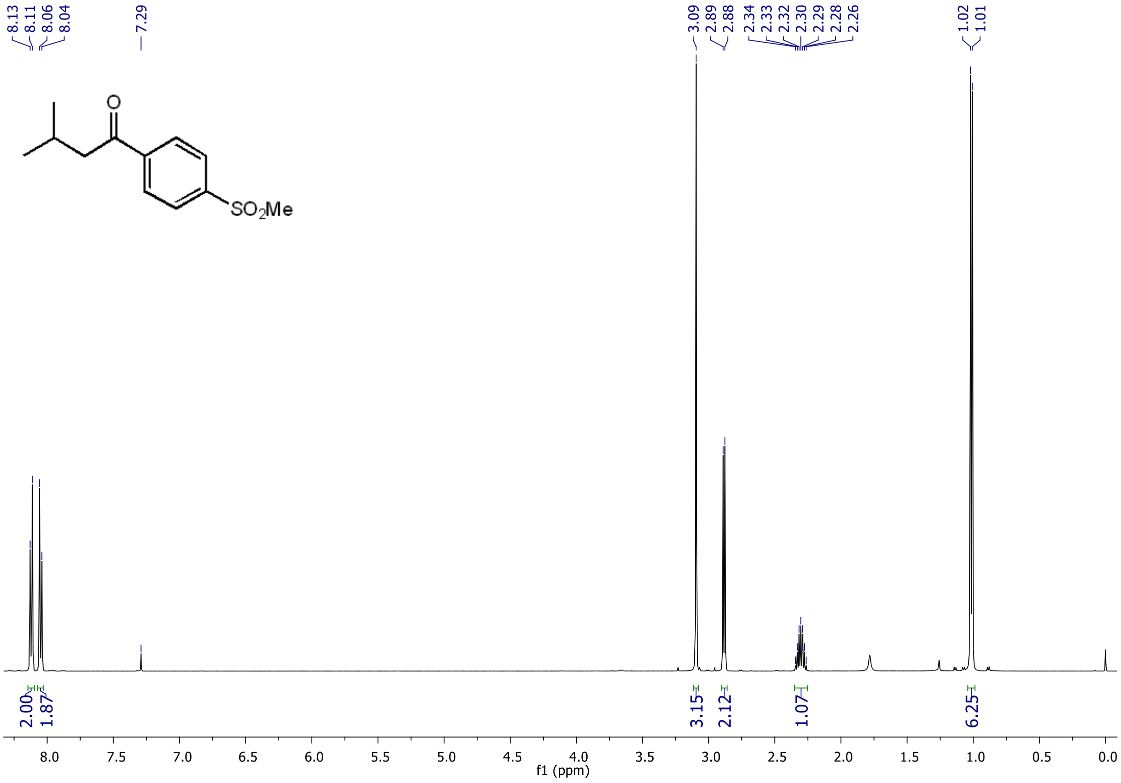

^{1}H NMR of 9 ($CDCl_3$, 500MHz)

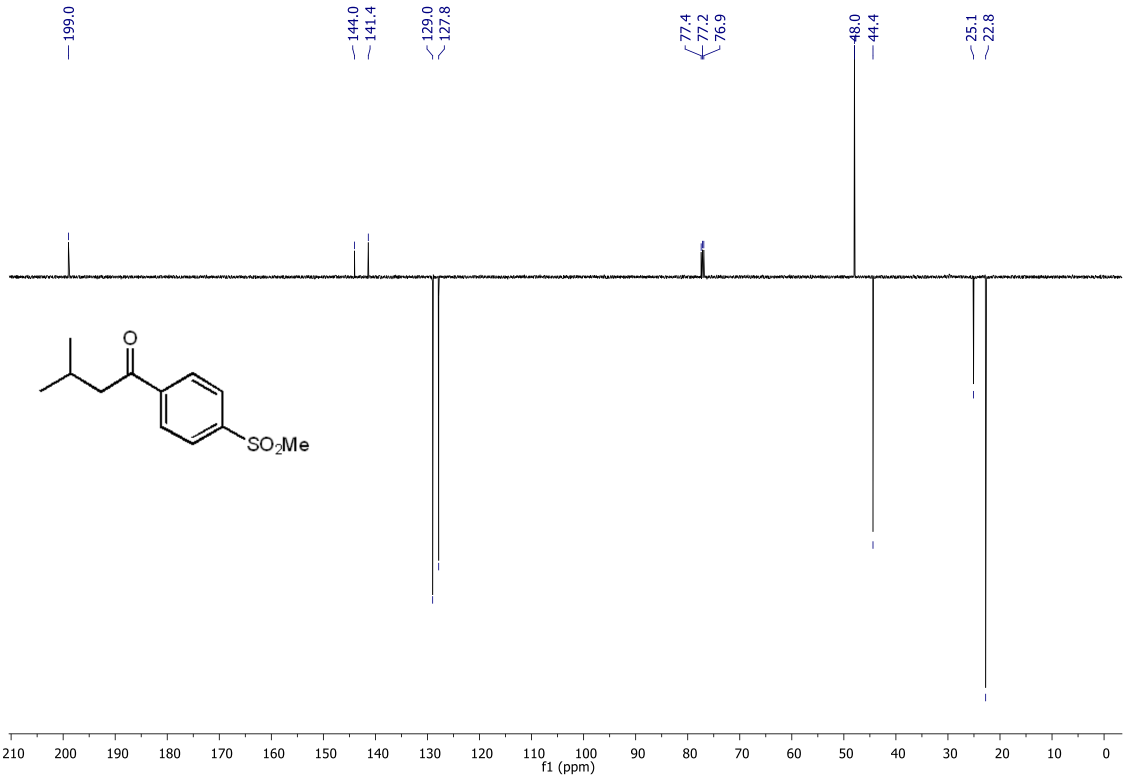

^{13}C NMR (DEPTQ) of 9 ($CDCl_3$, 126MHz)

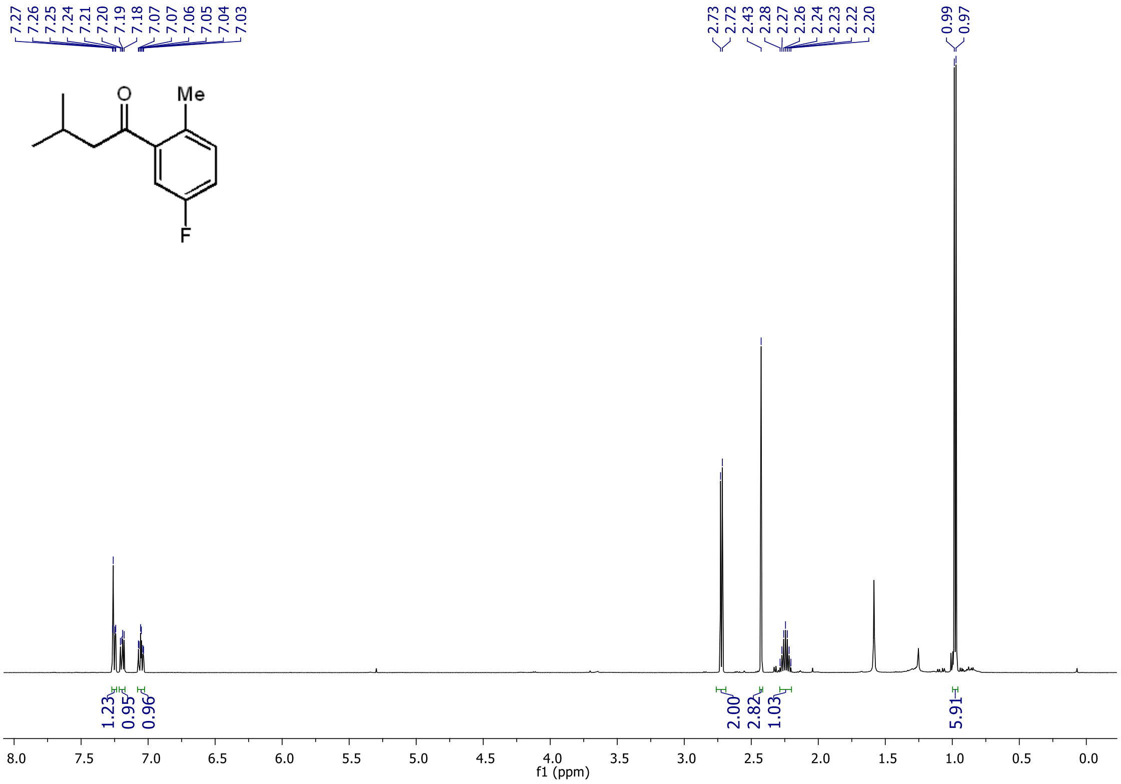

^{1}H NMR of 10 ($CDCl_3$, 500MHz)

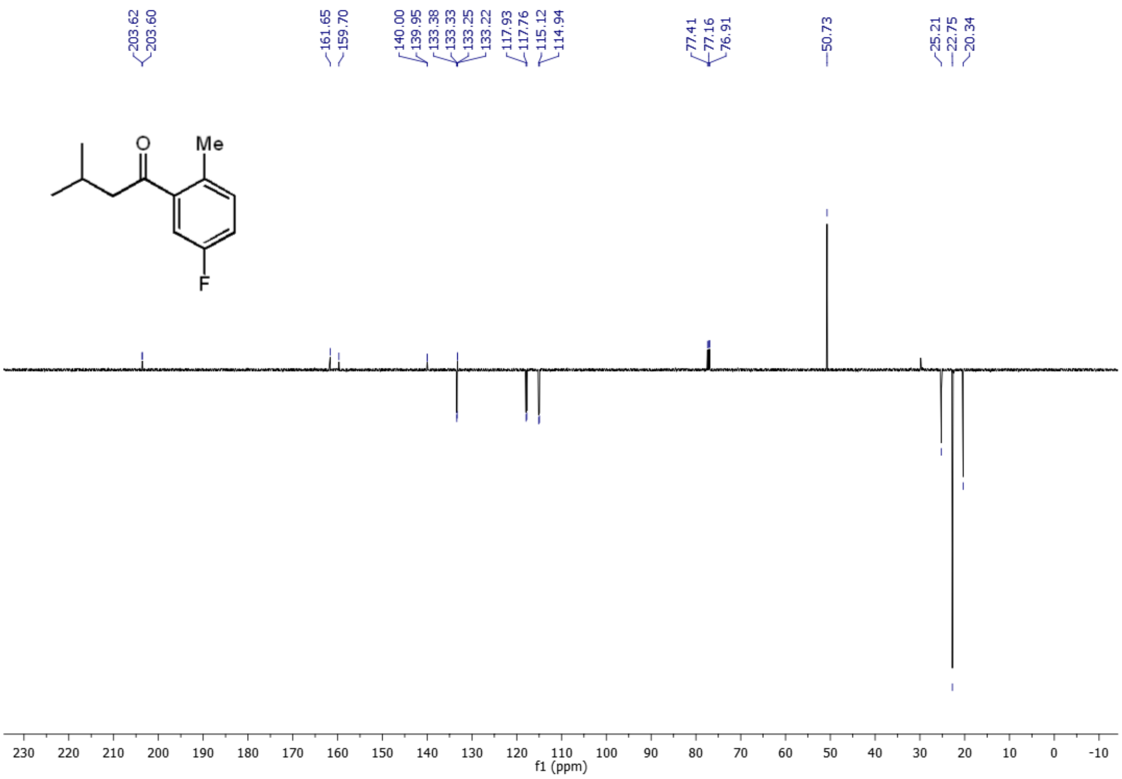

^{13}C NMR (DEPTQ) of 10 ($CDCl_3$, 126MHz)

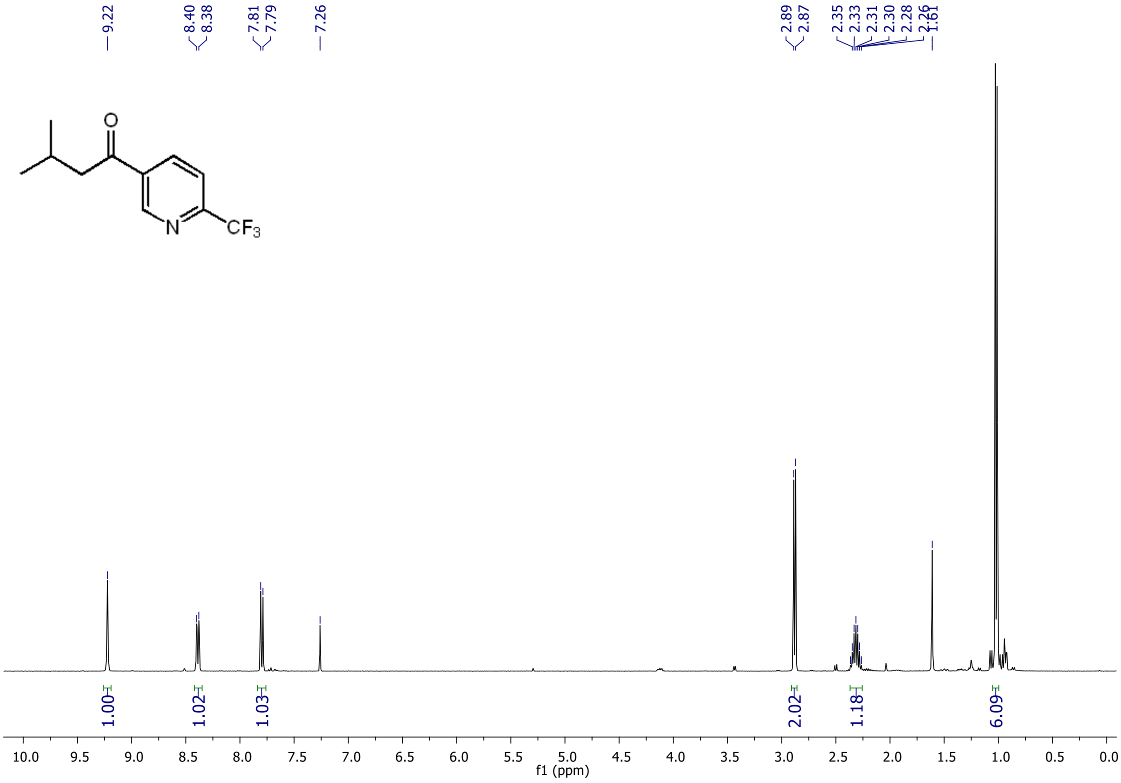

^{1}H NMR of 11 ($CDCl_3$, 400MHz)

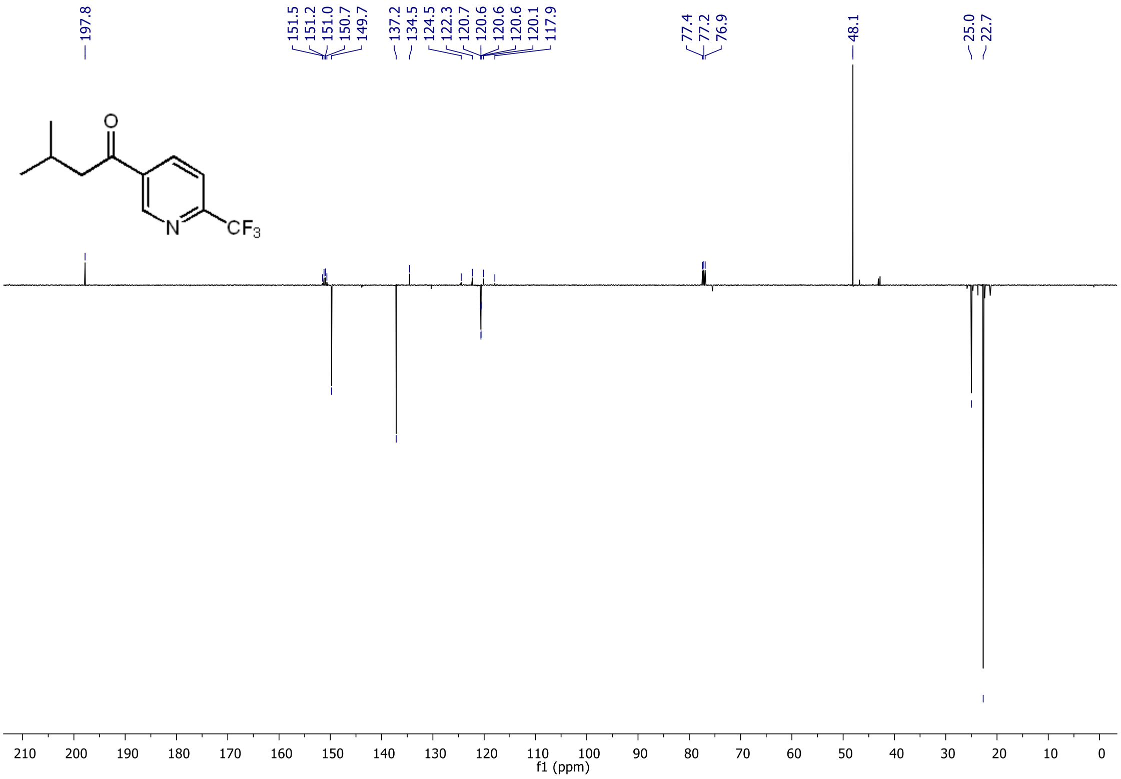

^{13}C NMR (DEPTQ) of 11 ($CDCl_3$, 126MHz)

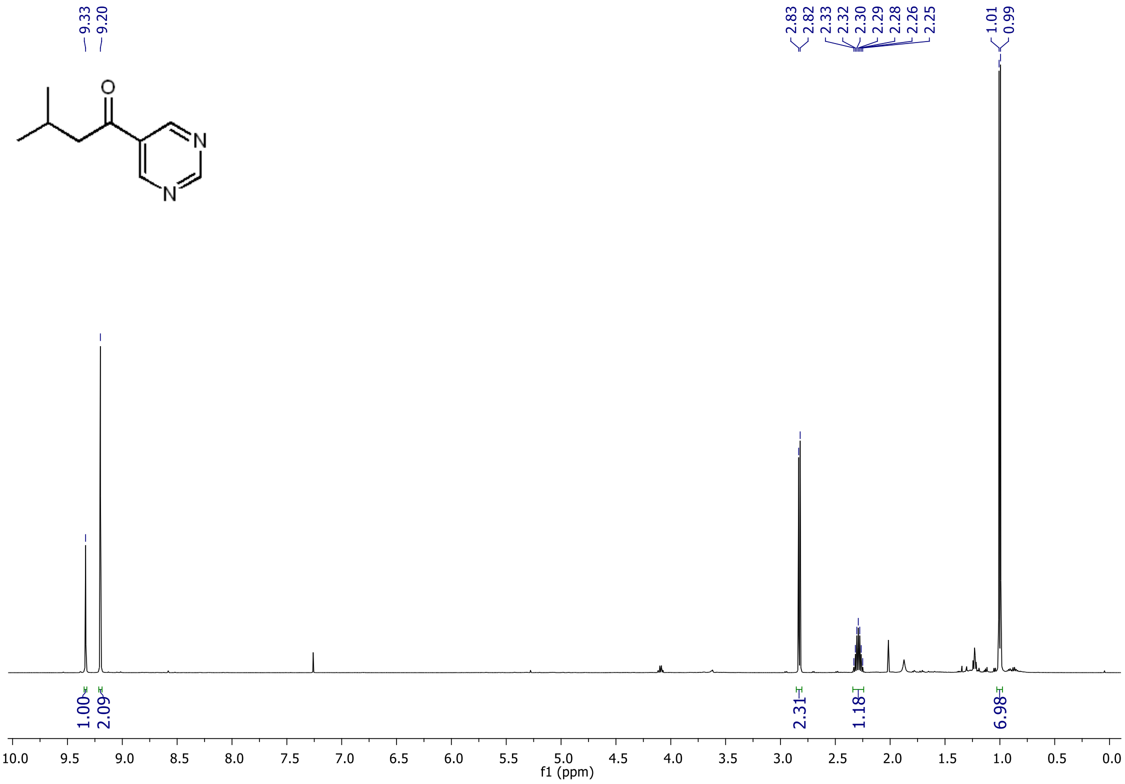

^{1}H NMR of 12 ($CDCl_3$, 500MHz)

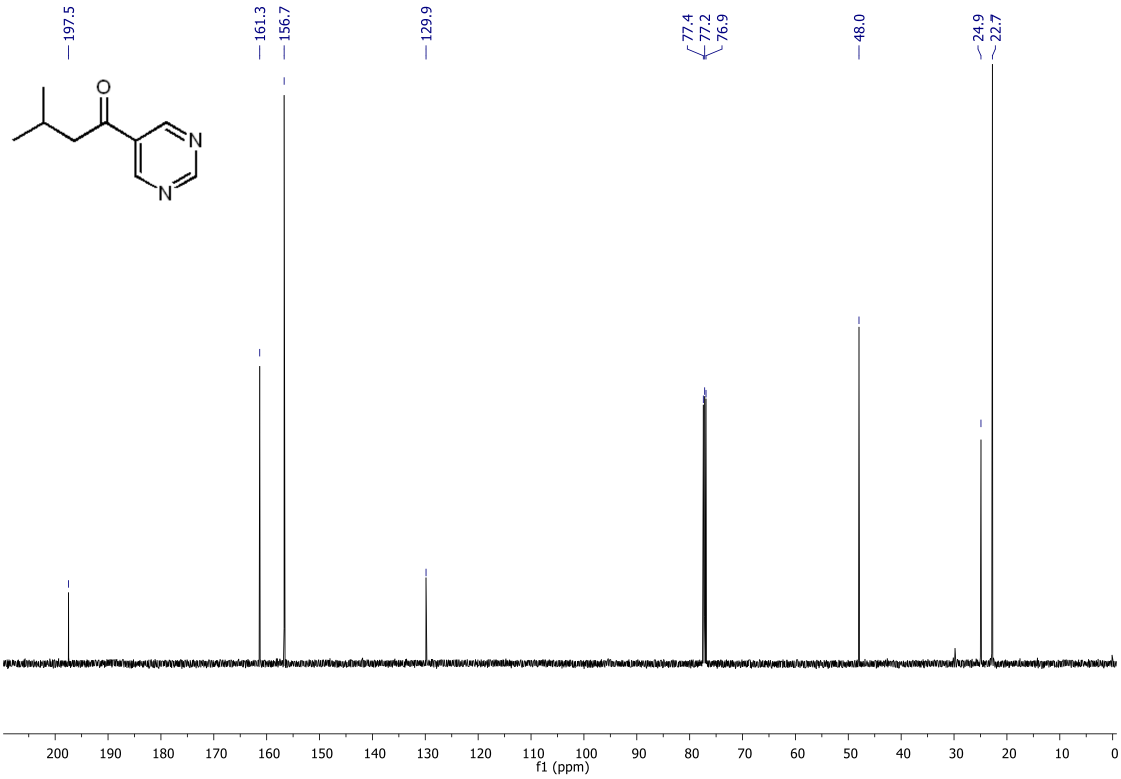

^{13}C NMR of 12 ($CDCl_3$, 126MHz)

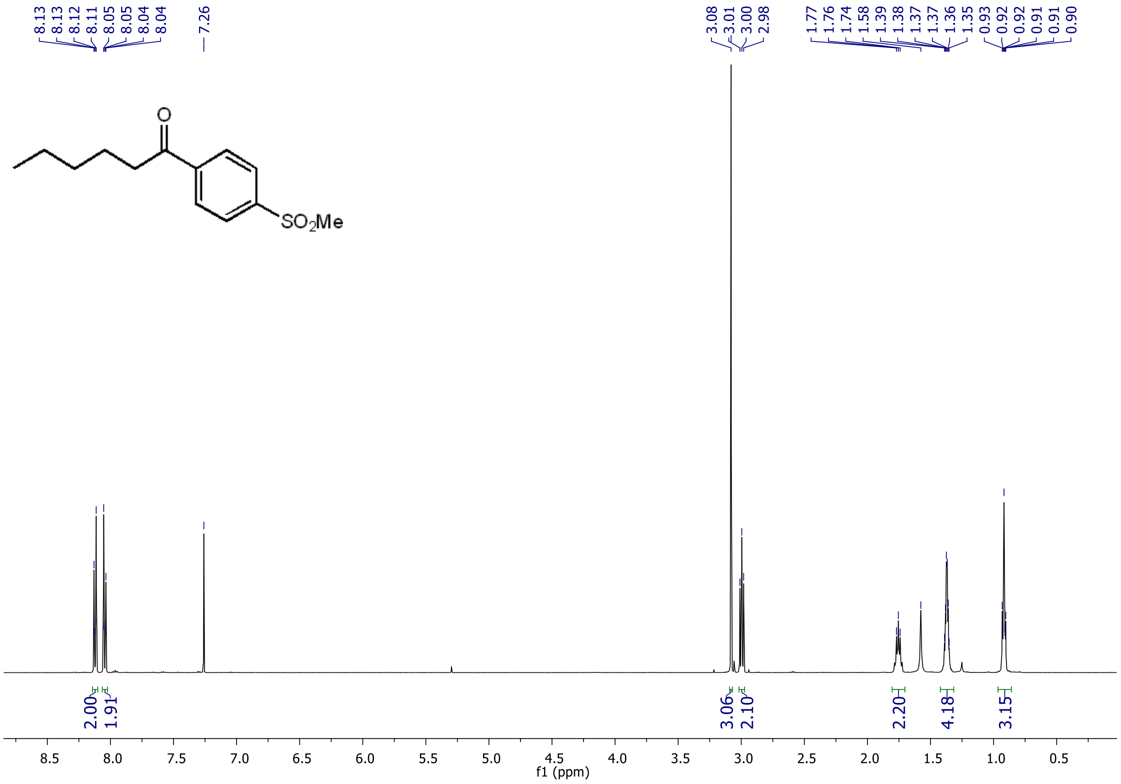

^{1}H NMR of 13 ($CDCl_3$, 500MHz)

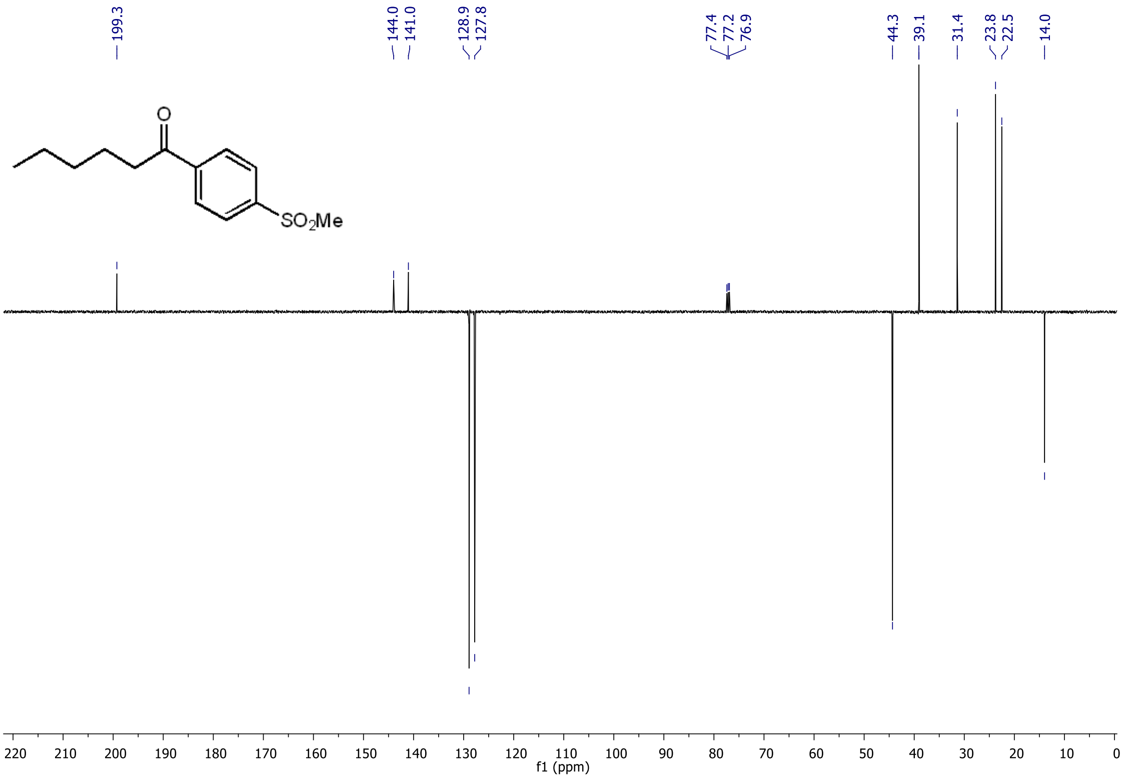

^{13}C NMR (DEPTQ) of 13 ($CDCl_3$, 126MHz)

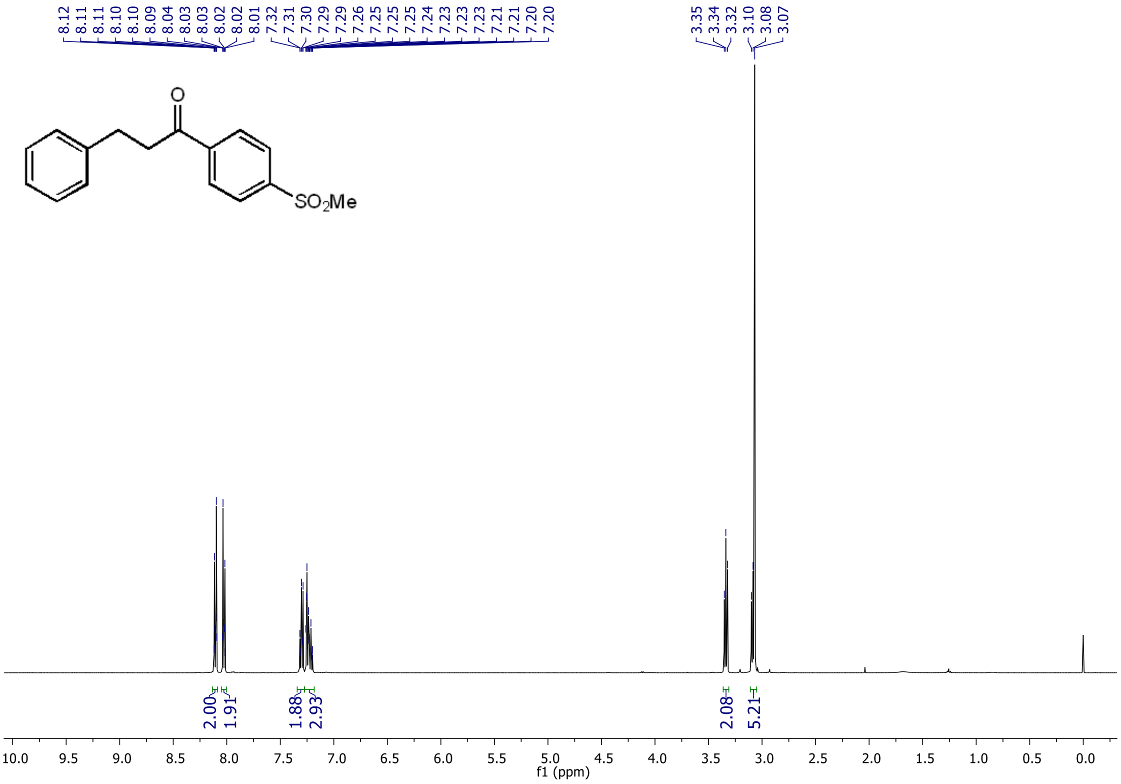

^{1}H NMR of 14 ($CDCl_3$, 500MHz)

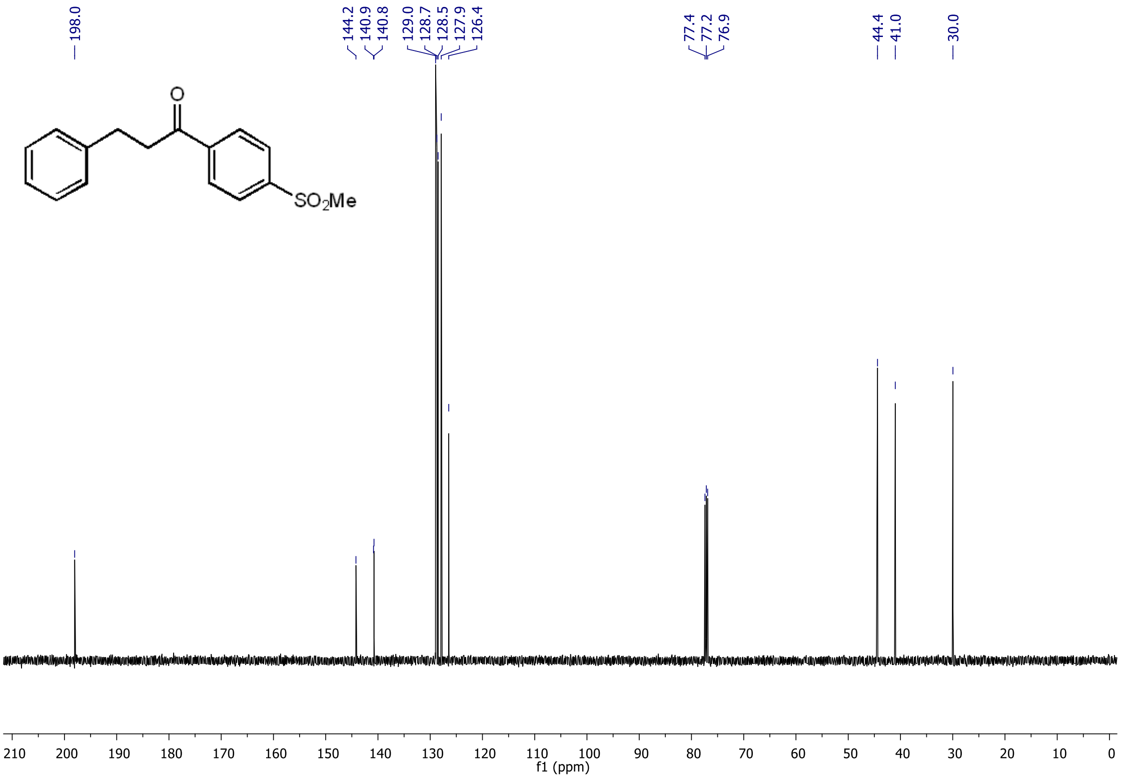

^{13}C NMR of 14 ($CDCl_3$, 126MHz)

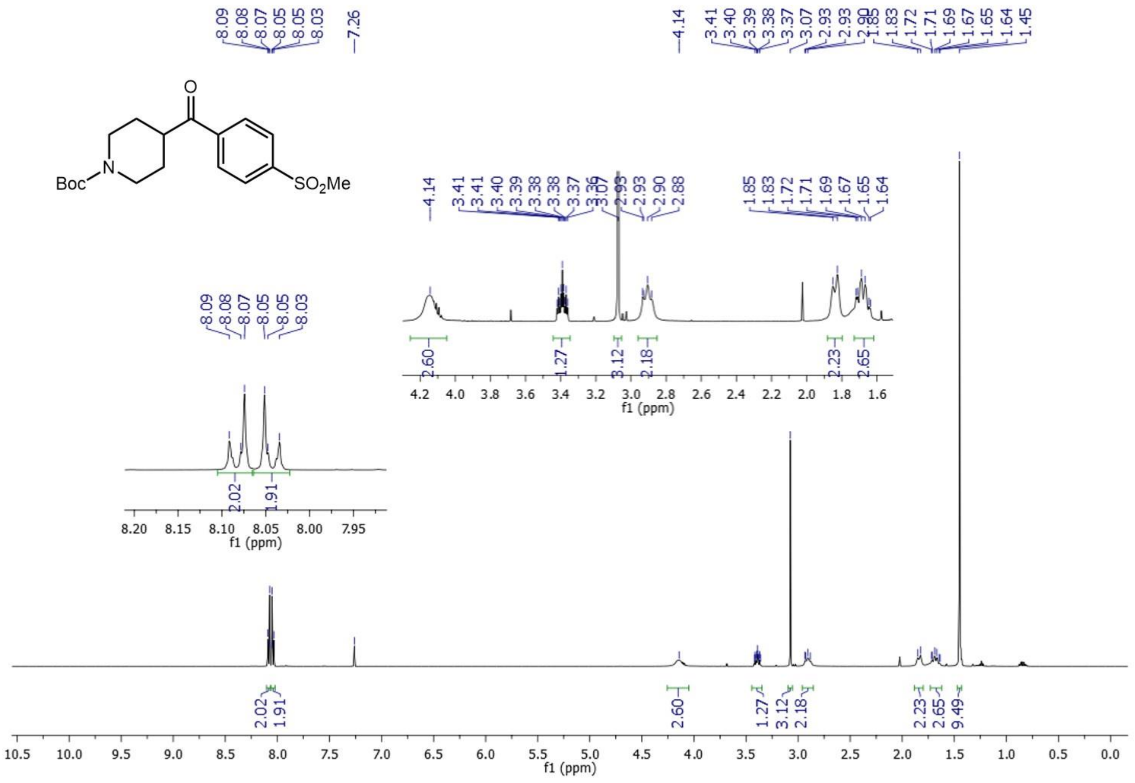

^{1}H NMR of 15 ($CDCl_3$, 500MHz)

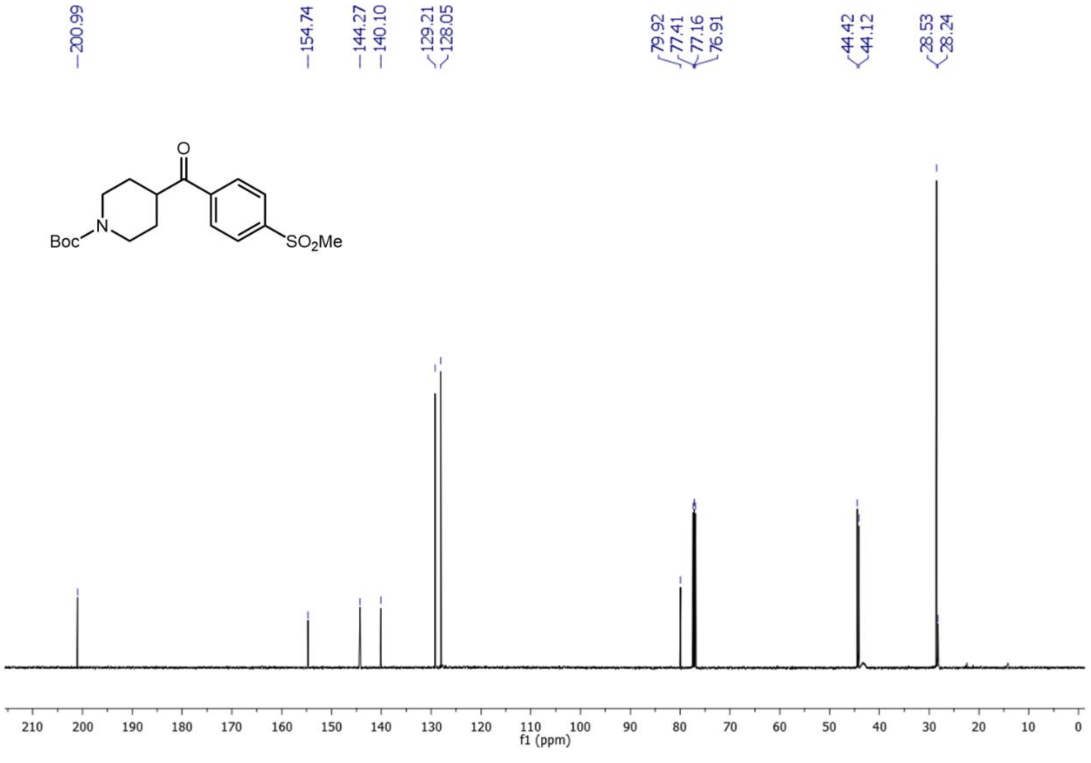

^{13}C NMR of 15 ($CDCl_3$, 126MHz)

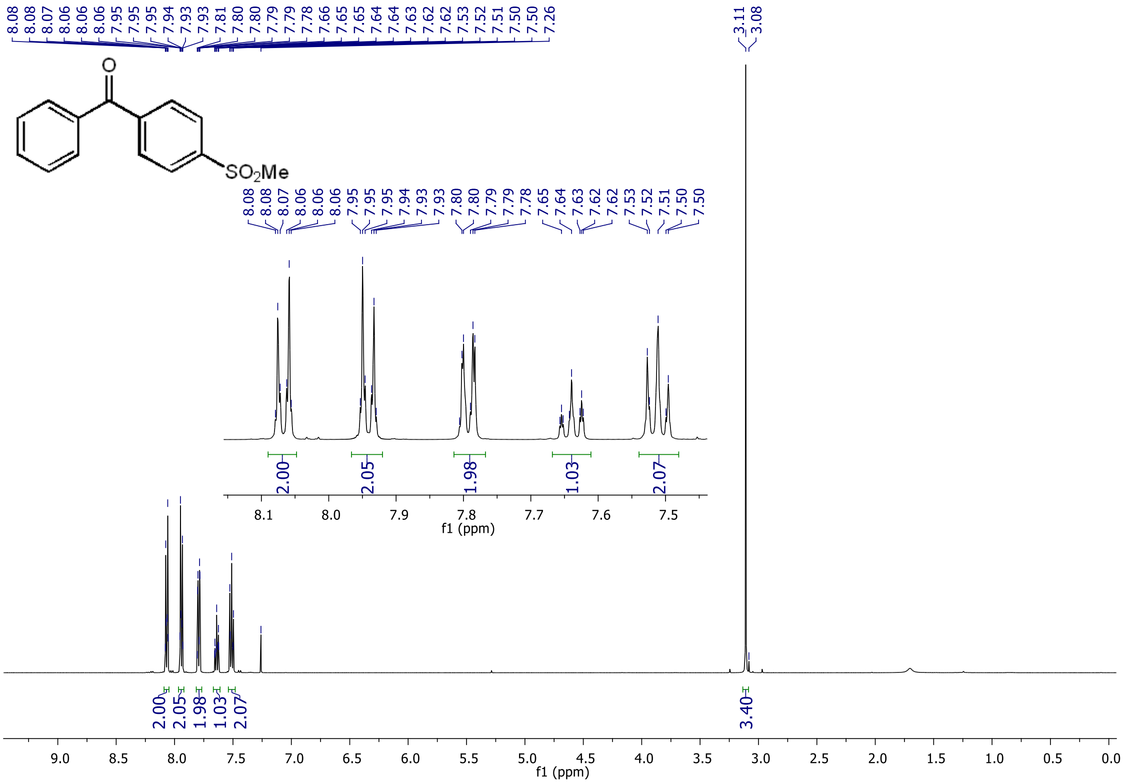

^{1}H NMR of 16 (CDCl$_3$, 500MHz)

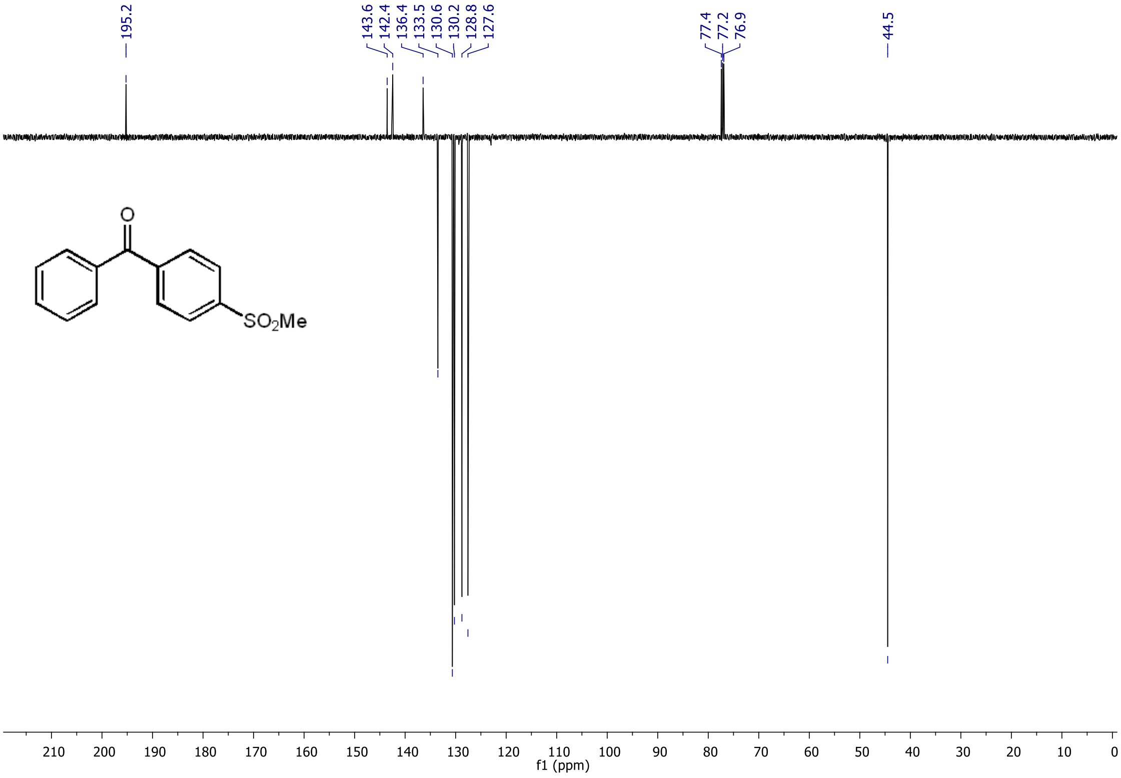

^{13}C NMR (DEPTQ) of 16 (CDCl$_3$, 126MHz)

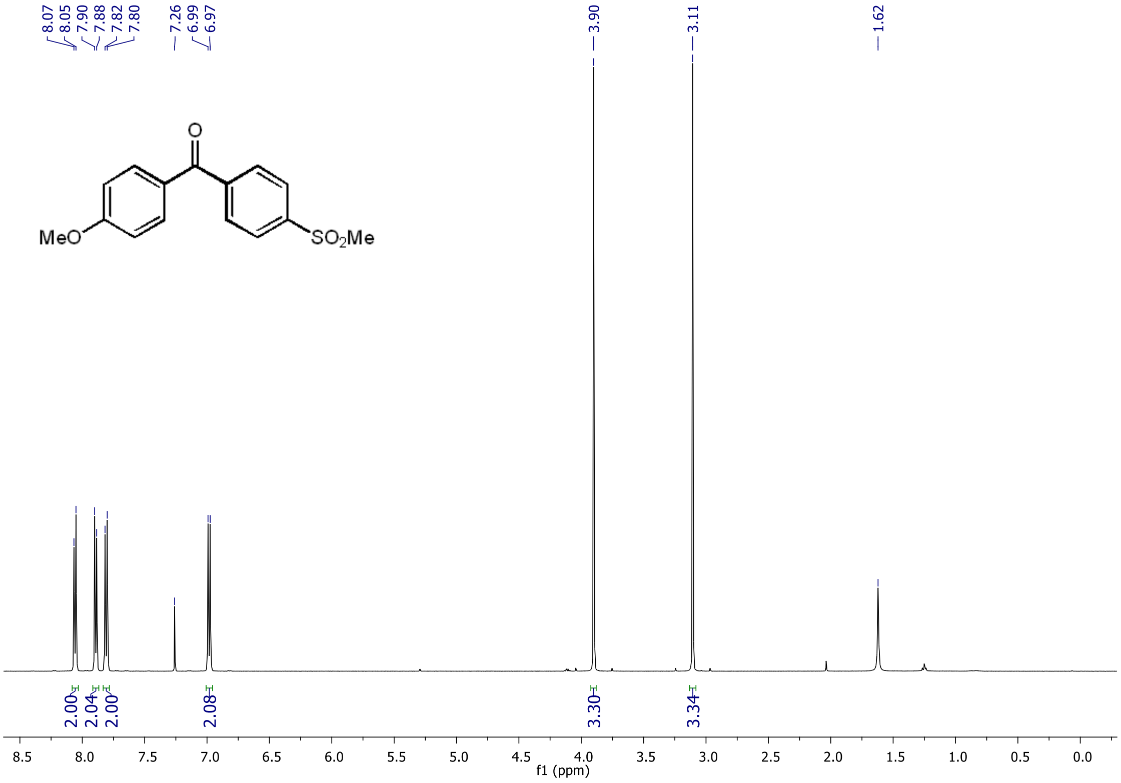

^{1}H NMR of 17 (CDCl3, 500MHz)

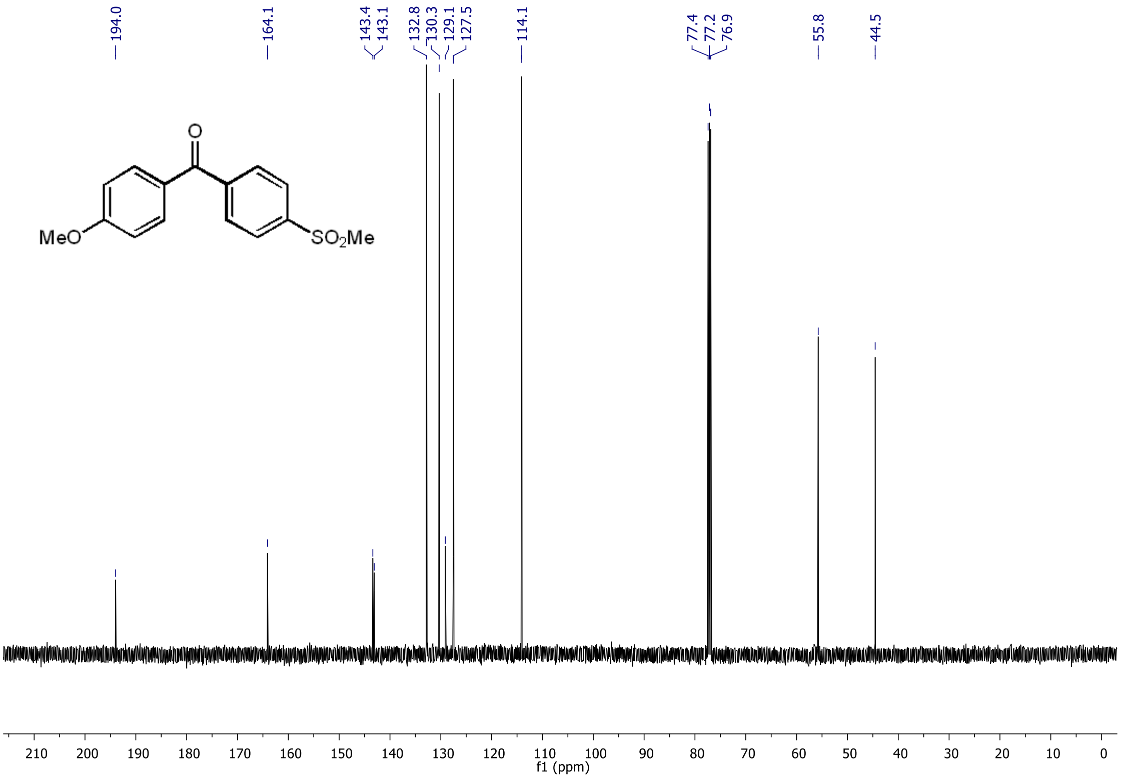

^{13}C NMR of 17 (CDCl3, 126MHz)

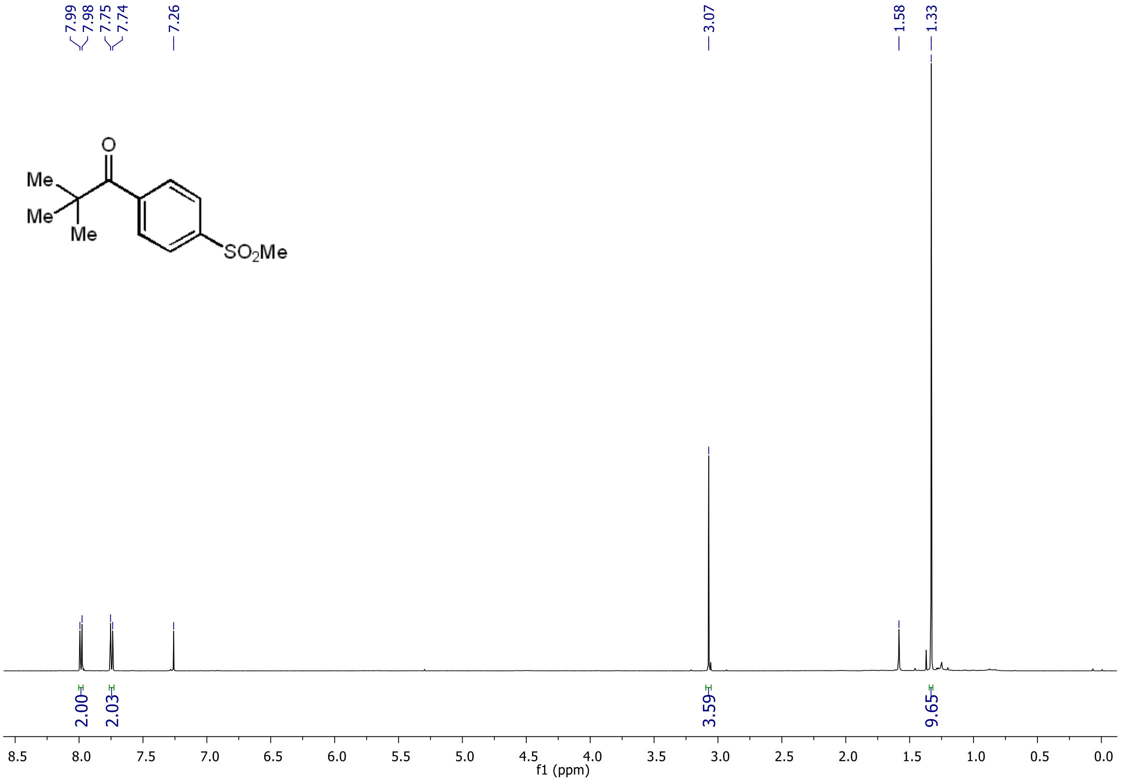

^{1}H NMR of 18 ($CDCl_3$, 500MHz)

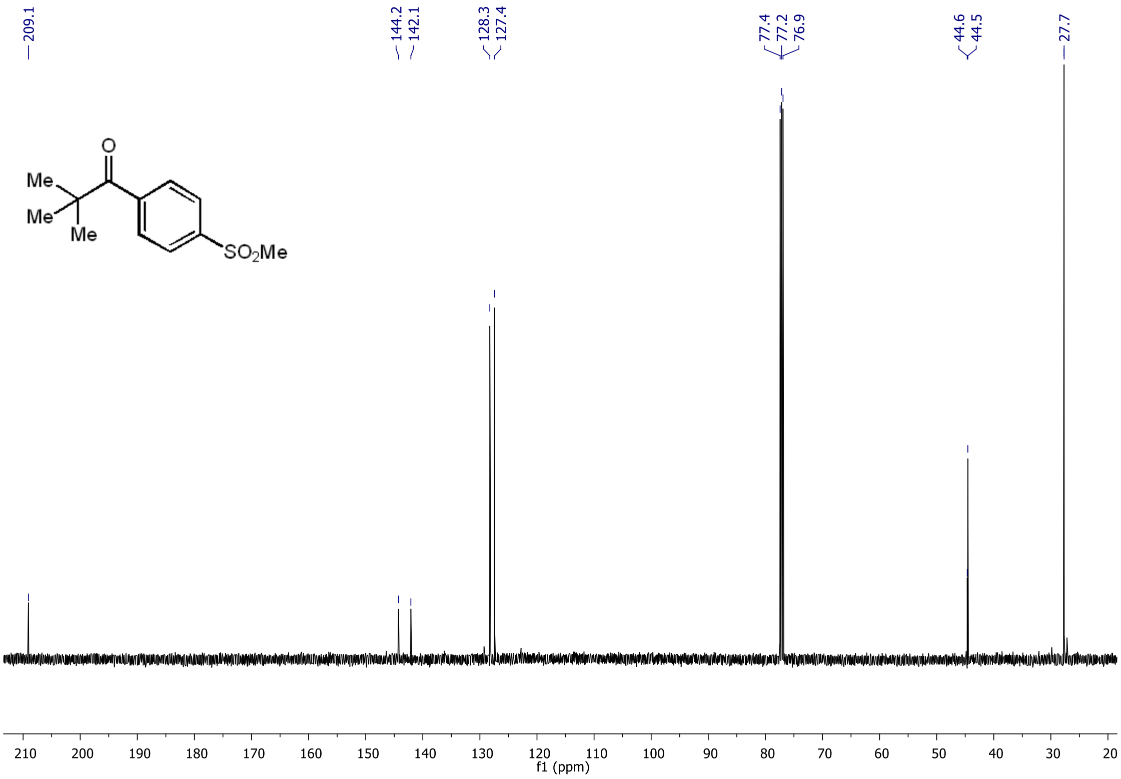

^{13}C NMR of 18 ($CDCl_3$, 126MHz)

7) Mechanistic experiments

Mechanistic proposal

Two nickel cycle pathways, Ni(0)/Ni(I)/Ni(III) and Ni(0)/Ni(II)/Ni(III), are commonly proposed on literature for cross-coupling of aryl bromides with C-centered radicals.[8,11] Thereby, we envisioned two catalytic cycles for our aldehyde arylation involving DABCO as HAT abstractor (Scheme S1b and c). Despite, recent mechanism studies performed with α-nitrogen[12] and *tert*-butyl radical[13] cross coupling with aryl bromides indicate a $Ni^{0}/Ni^{I}/Ni^{III}/Ni^{I}$ mechanism may be favored, rather than the $Ni^{0}/Ni^{II}/Ni^{III}/Ni^{I}$ cycle and may be operative on our arylation of aldehydes, we cannot rule out the another cycle pathway.

A proposal for nickel pre catalyst formation is shown below in Scheme S1a. The dibromide Ni(II) complex is reduced by Ir(II) complex promoting the release of bromide ion (Br^{-}) and formation of Ni(I) complex. At the next step, the Ni(I) complex is reduced by the Ir(II) complex with binding of aryl bromide to nickel, furnishing Ni(0) complex.[12]

Furthermore, based on previous reports[14][15] we proposed a mechanism involving a bromine radical generated through photolysis of a Nickel-Aryl bromide complex as HAT abstractor for reaction performed in absence of DABCO (Scheme S1d).

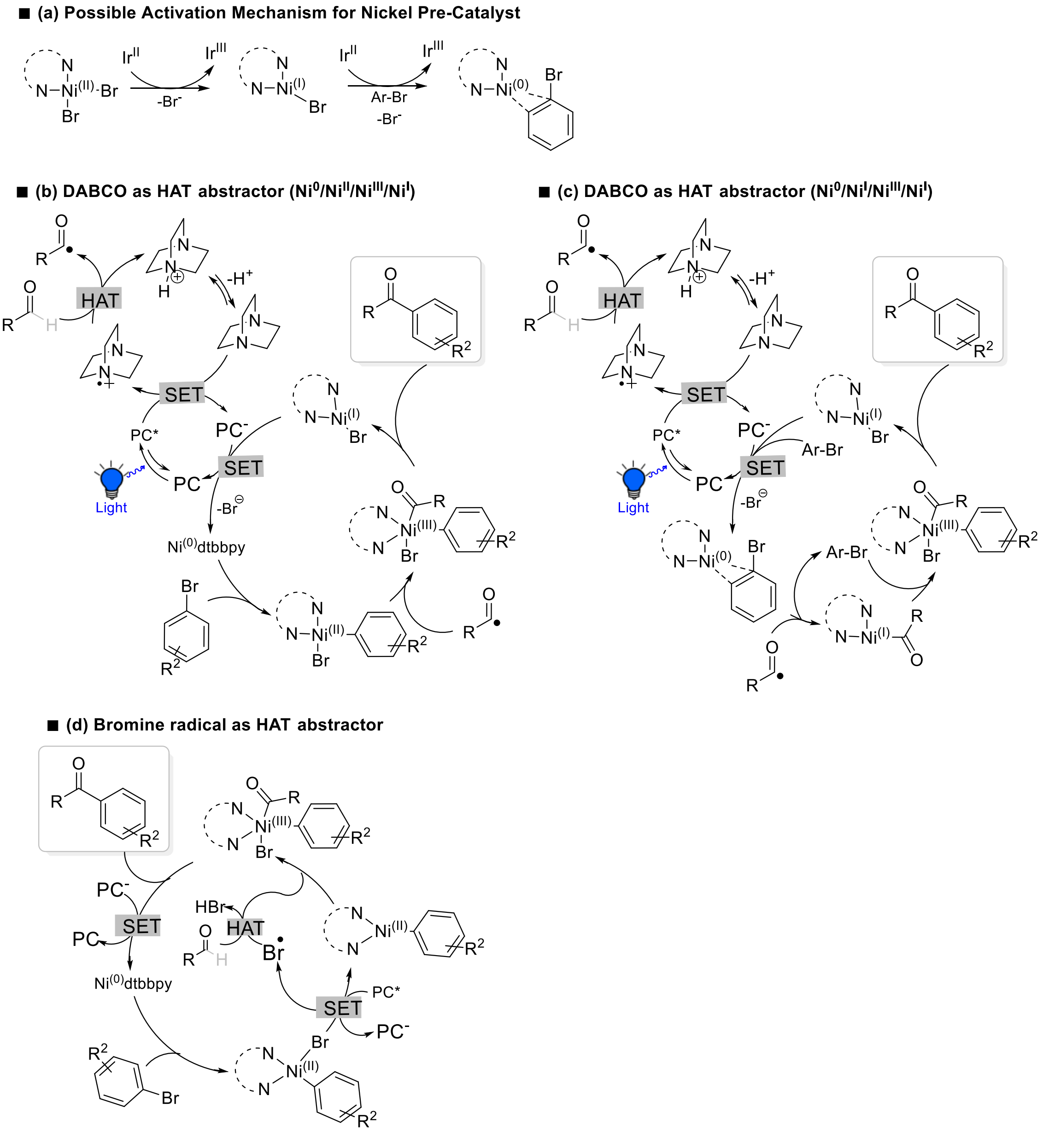

Scheme S1: (a) Activation mechanism proposed for nickel pre-catalyst. (b) Mechanism for aldehyde arylation involving DABCO as HAT abstractor based on nickel cycle traditionally proposed on literature (c) Mechanism for aldehyde arylation involving DABCO as HAT abstractor based on recent mechanistic studies (d) Mechanism involving a bromine radical generated through photolysis of a nickel-aryl bromide complex as HAT abstractor. PC = photocatalyst Ir[dF(CF_3)ppy]$_2$(dtbbpy)PF_6. SET = single-electron transfer event.

Radical trapping experiments

Table S4: Radical trapping with TEMPO. The reactions were carried out according to the general procedure outlined in section 3 with addition of 4.0 equivalents of TEMPO and analyzed through GC-MS.

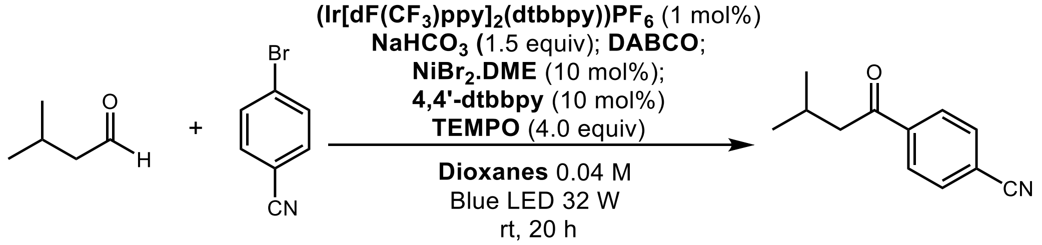

Entry	DABCO	Yield (%)[a]
1	-	0%
2	0.5 eq	0%

[a]Yield determined by GC-MS analysis of the reaction

The addition of 4.0 equivalents of TEMPO completely shuts down the product formation, leading to an observable GC–MS adduct, suggesting a radical path for the reaction both in the presence and in the absence of the HAT catalyst (DABCO). Moreover, as the same adduct was formed in both cases, the above-mentioned reactions proceed through a common radical. Isolation and characterization of this adduct showed the isovaleraldehyde moiety, leading to a strong evidence of the acyl radical formation.

As the amount of adduct formed was much lower in the presence of DABCO, we hypothesize this can indicate a lower acyl radical concentration in this reaction media. This could be explained by an interaction of TEMPO with a previous radical species that is responsible of acyl radical formation from isovaleraldehyde. This result is in line with the proposed mechanism, where a DABCO radical cation species acts as HAT abstractor.

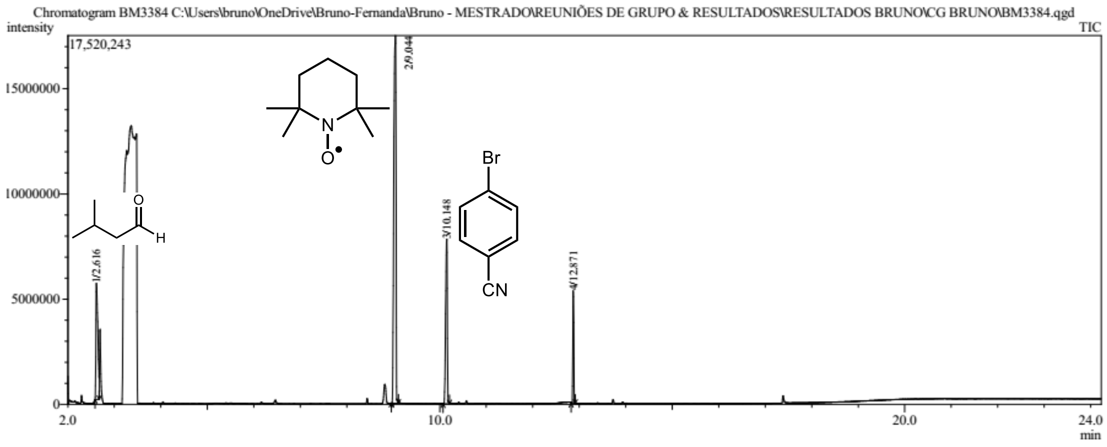

Peak Report TIC

Peak#	R.Time	I.Time	F.Time	Area	Area%	Height	Height%	A/H	Mark	Name
1	2.616	2.585	2.675	16126068	16.25	5520547	15.27	2.92	MI	Butanal, 3-methyl-
2	9.044	8.955	9.110	56280958	56.73	17457954	48.28	3.22	MI	1-Piperidinyloxy, 2,
3	10.148	10.070	10.210	19072553	19.22	7825660	21.64	2.44	MI	Benzonitrile, 4-bror
4	12.871	12.825	12.915	7728695	7.79	5352223	14.80	1.44	MI	Azacyclohexan-3-ol
				99208274	100.00	36156384	100.00			

Figure S2: GC of the reaction depicted in Table 4, entry 1. The first peak (t_r = 2.6 min) shows the presence of isovaleraldehyde, the second peak (t_r = 9.0 min) is the excess TEMPO, the third peak (t_r = 10.1 min) shows the presence of the aryl bromide. The fourth peak (t_r = 12.9 min) was formed during the reaction, and was assumed to be the TEMPO adduct. The non-integrated peak between the first and second refers to 1,4-dioxanes, the reaction solvent.

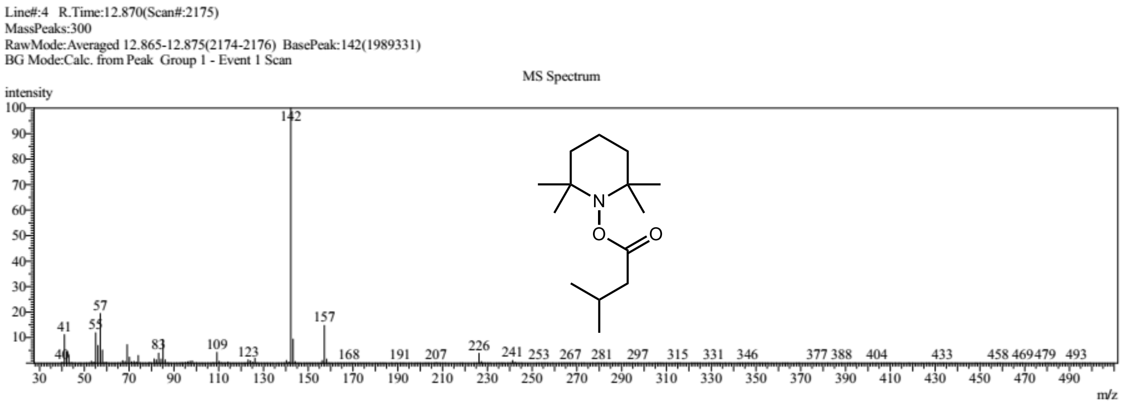

Figure S3: MS spectrum of the fourth peak (t_r = 12.9 min), showing a m/z 241 molecular ion, compatible with the acyl radical-TEMPO adduct.

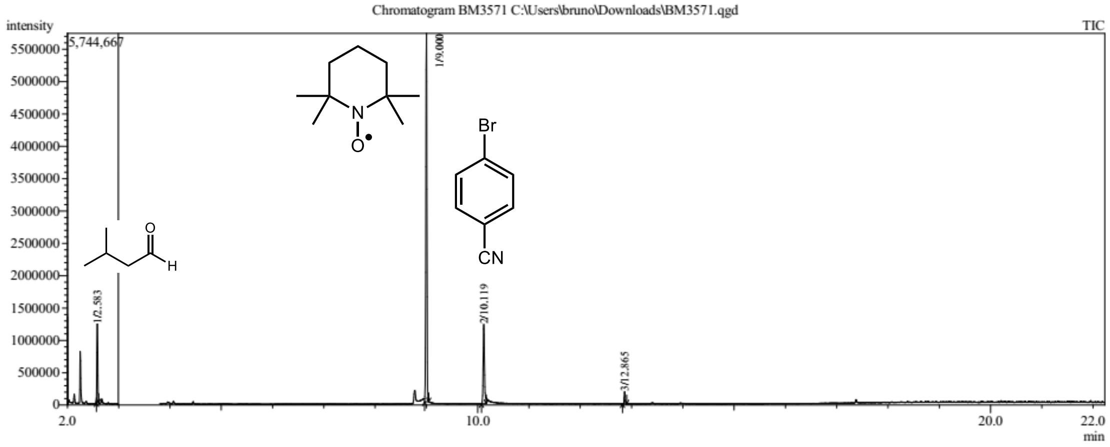

Peak Report TIC

Peak#	R.Time	I.Time	F.Time	Area	Area%	Height	Height%	A/H	Mark	Name
1	2.583	2.565	2.610	1488773	11.95	1217289	14.62	1.22	MI	Butanal, 3-methyl-
2	9.000	8.965	9.045	8513193	68.34	5692760	68.37	1.50	MI	1-Piperidinyloxy, 2,
3	10.119	10.075	10.165	2197289	17.64	1224632	14.71	1.79	MI	Benzonitrile, 4-bror
4	12.865	12.825	12.905	258575	2.08	191274	2.30	1.35	MI	Azacyclohexan-3-ol
				12457830	100.00	8325955	100.00			

Figure S4: GC of the reaction depicted in Table 4, entry 2. The first peak (t_r = 2.6 min) shows the presence of isovaleraldehyde, the second peak (t_r = 9.0 min) is the excess TEMPO, the third peak (t_r = 10.1 min) shows the presence of the aryl bromide. The fourth peak (t_r = 12.9 min) was formed during the reaction, and was assumed to be the TEMPO adduct. A solvent cut was performed from 3.00 to 3.80 min.

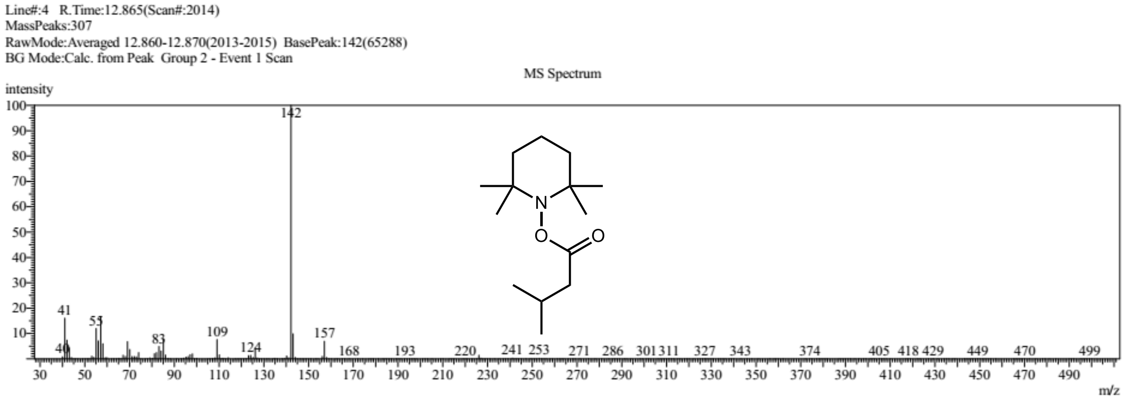

Figure S5: MS spectrum of the fourth peak (t_r = 12.9 min), showing a *m*/*z* 241 molecular ion, compatible with the acyl radical-TEMPO adduct.

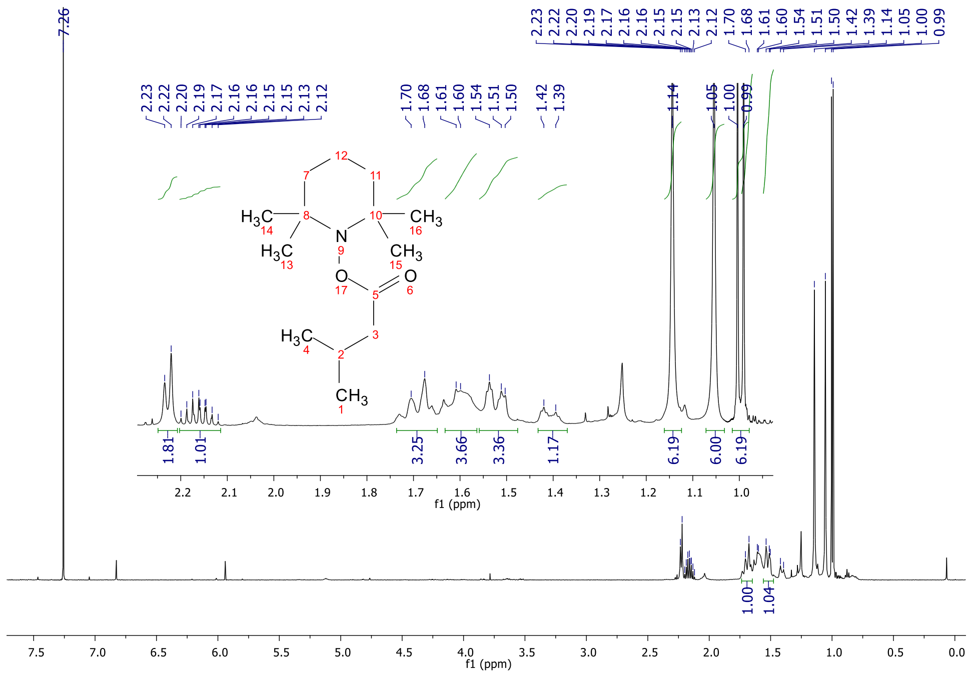

Figure S6: ^{1}H NMR (500MHz, $CDCl_3$) of the isolated adduct (not purified). It is possible to observe the isovaleraldehyde moiety through the isopropyl hydrogens in δ 1.00 ppm (d, J = 6.6 Hz, 6H) and δ 2.20-2.12 ppm (m, 1H), as well as the alpha-carbonyl protons in δ 2.23 ppm (d, J = 6.9 Hz, 2H). The TEMPO moiety could also be detected through its methyl hydrogens in δ 1.14 ppm (s, 6H) and δ 1.05 ppm (s, 6H). The methylene hydrogens are under broad signals at δ 1.69-1.41 ppm, along with some impurities.

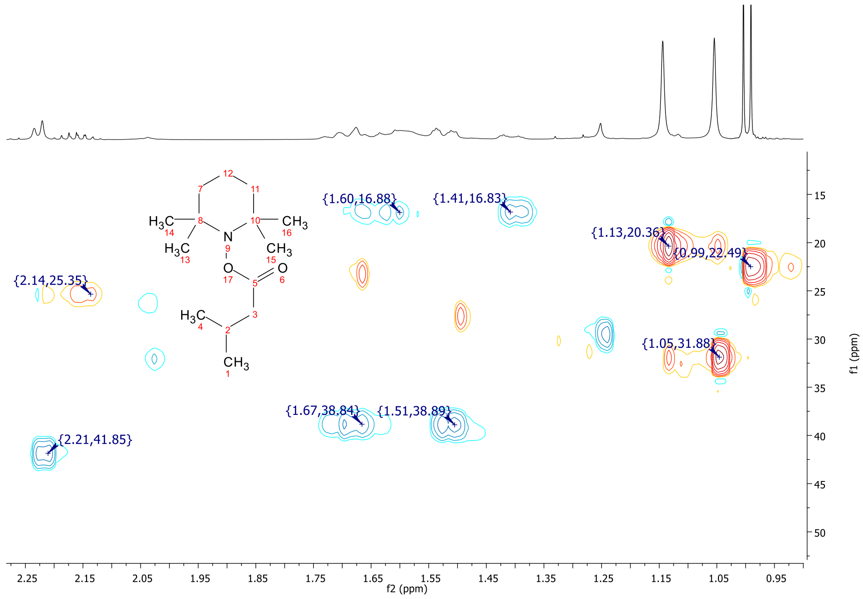

Figure S7: HSQC (500MHz, $CDCl_3$) analysis of the isolated adduct (not purified). δ 22 ppm (C1, C4), 25 ppm (C2), 42 ppm (C3), 20 ppm, 32 ppm (C13-C16), δ 17 ppm (C12), 39 ppm (C7, C11).

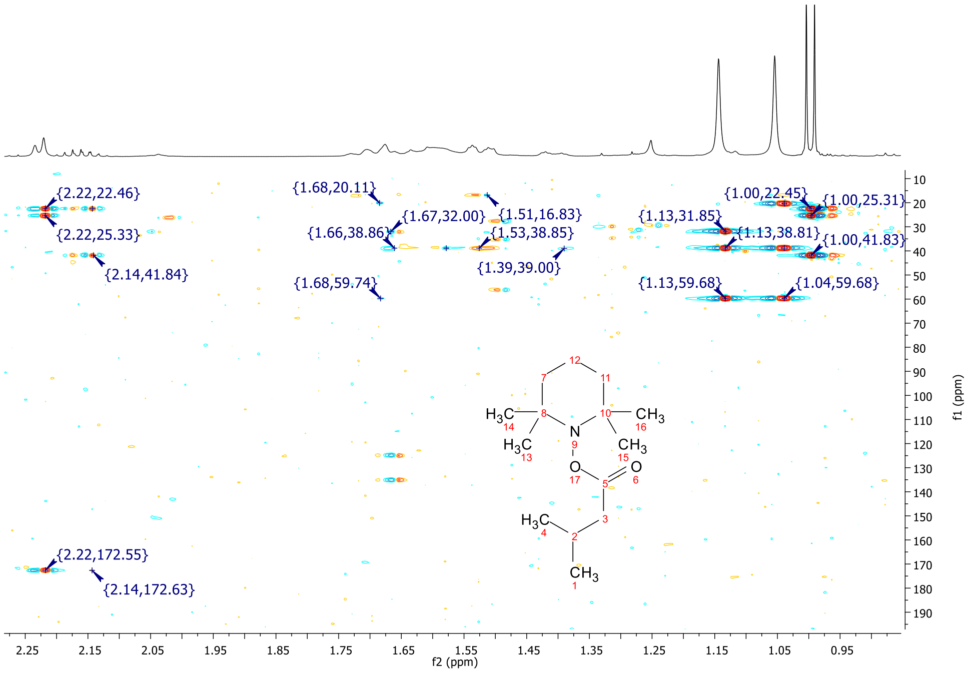

Figure S8: HMBC (500MHz, $CDCl_3$) analysis of the isolated adduct (not purified). It is possible to see correlations that corroborate the proposed structure. From this analysis it is possible to observe the quaternary carbons, δ 173 ppm (C5) and 60 ppm (C8, C10).

Procedure for radical trapping experiment with methyl acrylate. To a 5 mL glass vial equipped with a magnetic stir bar were added Ir[dF(CF_3)ppy]$_2$(dtbbpy)PF_6 (1.2 mg, 0.0011 mmol, 1 mol %), 1,4-diazabicyclo[2.2.2]octane (DABCO, 6.2 mg, 0.055 mmol, 0.5 equiv), $NaHCO_3$ (13.9 mg, 0.16 mmol, 1.5 equiv), and acetonitrile (3.0 mL). The vial was closed and sparged with argon for 15 minutes. Methyl acrylate (10 µL, 0.11 mmol, 1 equiv) and isovaleraldehyde (60 µL, 0.55 mmol, 5 equiv) were syringed into the reaction vial before sealing the vial with parafilm. The reaction was magnetically stirred and irradiated for 20 h with a blue LED lamp 6 cm away from the vial with a cooling fan to keep the reaction at room temperature. After that time, the reaction was quenched by exposure to air and filtered through a pad of silica with ethyl acetate and dichloromethane. After concentration, the residue was purified by flash chromatography (5% ethyl acetate on hexanes) on silica gel to afford the desired product as a colourless oil.

Table S5: Radical trapping with methyl acrylate.

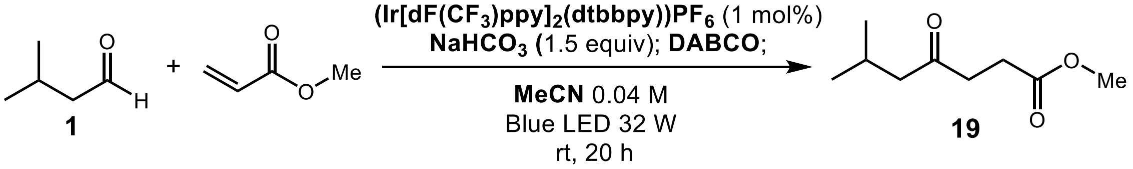

Entry	DABCO	Yield (%)[a]
1	-	traces
2	0.5 eq	20%

[a]Isolated yield

The radical trapping experiment with methyl acrylate afforded Michael adduct product in 20% yield when using the HAT catalyst (DABCO), but only trace amounts could be detected in its absence, thus proving the ability of DABCO to perform the HAT activation of the isovaleraldehyde, generating the corresponding acyl radical.

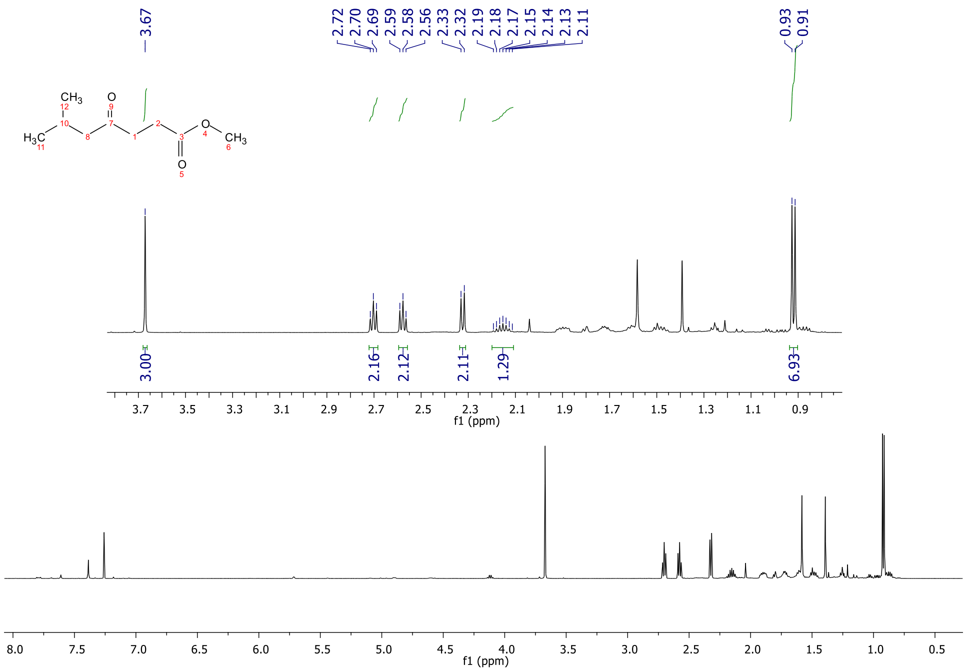

Figure S9: ^{1}H NMR (500 MHz, CDCl$_3$) of 19 δ 3.67 (s, 3H), 2.70 (t, *J* = 6.5 Hz, 2H), 2.58 (t, *J* = 6.5 Hz, 2H), 2.32 (d, *J* = 7.0 Hz, 2H), 2.25 – 1.98 (m, 1H), 0.92 (d, *J* = 6.7 Hz, 6H).

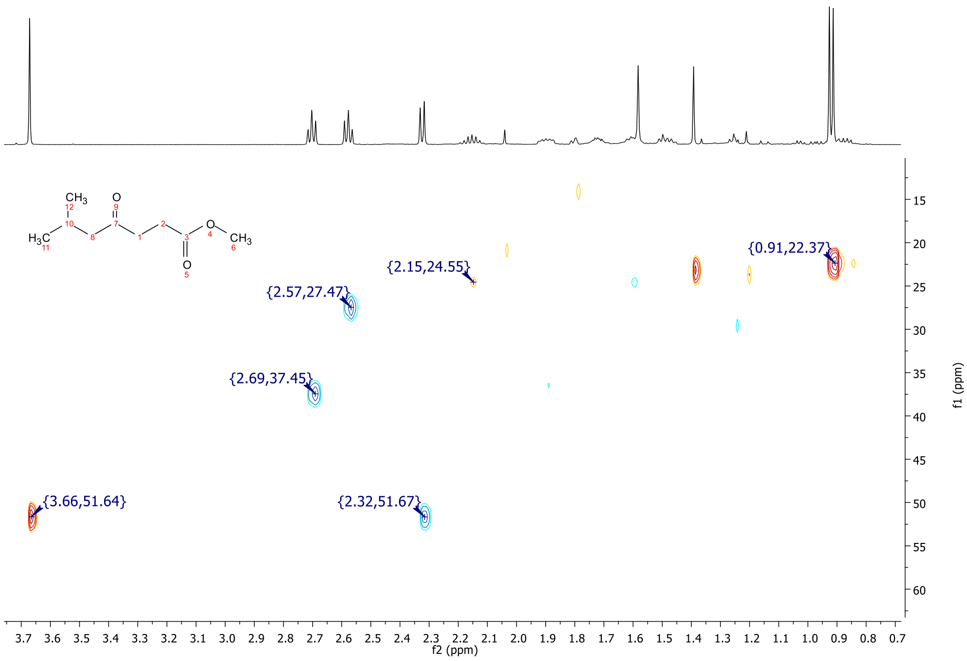

Figure S10: HSQC (500 MHz, CDCl3) of 19 δ 52 ppm (C6), 52 ppm (C8), 37 ppm (C1), 27 ppm (C2), 24 ppm (C10), 22 ppm (C11, C12).

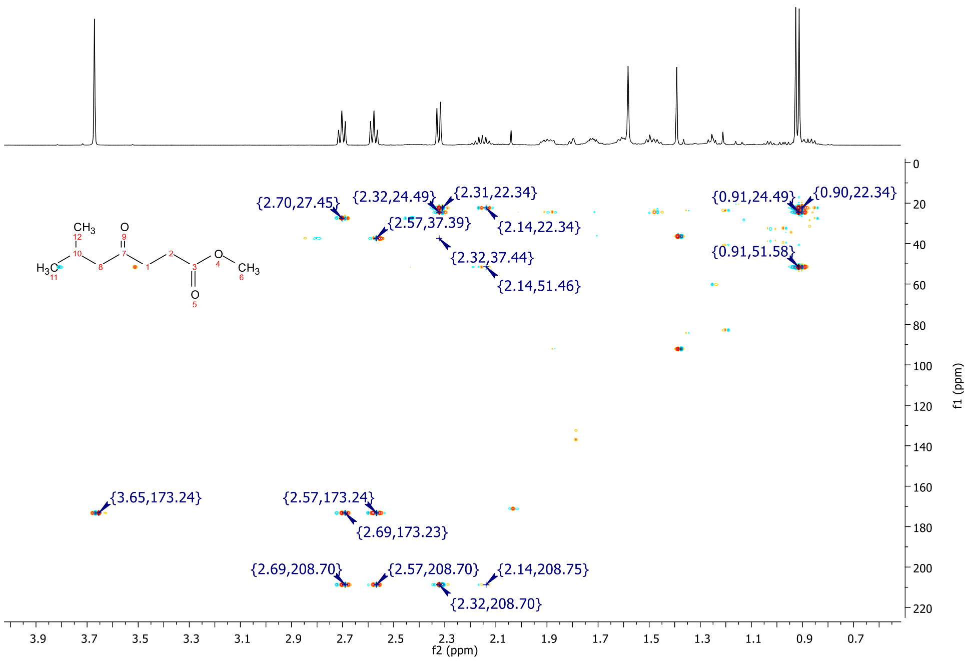

Figure S11: HMBC (500 MHz, CDCl3) of 19 Showing correlations corroborating the proposed structure. The quaternary carbons are now detectable δ 209 ppm (C7), 173 ppm (C3).

Reactions performed in absence of DABCO

The reactions were carried out according to the general procedure outlined in section 3 without DABCO.

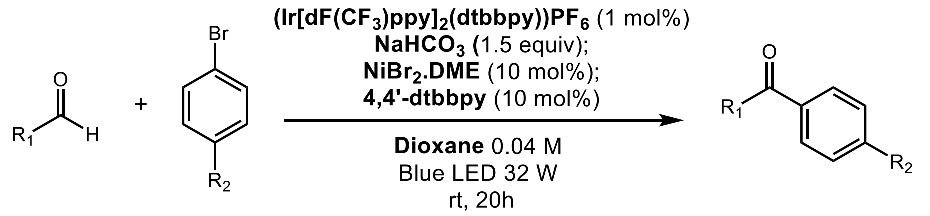

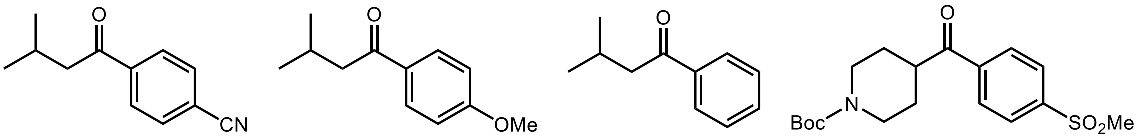

3 63%[a] **4** 14%[a] **6** 27%[a] **15** 44%[a]

[a]Yield determined by 1H NMR analysis with 1,3-benzodioxole as internal standard

Scheme S2: Reactions performed in absence of DABCO

Table S6: Reactions with bromide additive. The reactions were carried out according to the general procedure outlined in section 3 with addition of 1.0 equivalent of bromide salts and in absence of DABCO.

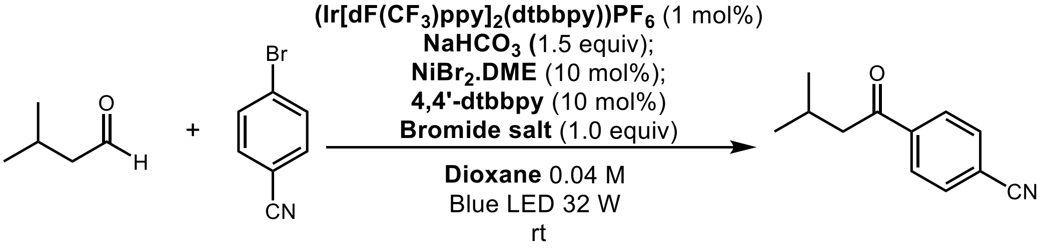

Entry	Bromide Salt	Time (h)	Yield (%)[a]
1	-	20	64%
2	-	10	62%
3	-	5	52%
4	NaBr	5	33%
5	TBAB	5	19%

[a]Yield determined by 1H NMR analysis with 1,3-benzodioxole as internal standard

According to the literature, the addition of halide salts could increase efficiency of reactions when a hydrogen atom transfer by bromine radical is operating. [15] Preliminary experiments with addition of 1.0 equivalent of sodium bromide and tetrabutylammonium bromide (entries 4 and 5) resulted in a yield decrease when compared with reaction in absence of bromide salt (entry 3), suggesting a different radical path for the reaction.

DFT calculations also point against this bromine radical HAT hypothesis. The HAT step between bromine radical and isovaleraldehyde is an endergonic, barrierless reaction, with Gibbs free energy of 18.8 kcal.mol^{-1} in the gas phase and 20.1 kcal.mol^{-1} in 1,4-dioxane simulated with PCM, both at 298.15 K and 1 atm. See the section below for the computational details.

8) Computational details

General information

The geometry optimizations of the isolated molecules, the TS searches and the optimizations of the reactant and product complexes presented in Figure 3, Table S7, and elsewhere in this document were performed using the M06-2X functional and the cc-pVTZ basis set on all atoms in the Gaussian 09 suit.[16] For the optimization of the reactant and product complexes structures only, we set a looser than default pruned grid with 35 radial shells and 110 angular points per shell (35,110), corresponding to *CoarseGrid*. The 1,4-dioxane solvent effect was included through the polarizable continuum model (PCM)[17–19] implicit solvent model. The PCM calculations consisted of single point from the gas phase optimized geometries, except for the isolated reactants and products used on the computation of the enthalpy and free energy of reaction, which were optimized with PCM. Frequency calculations were performed to confirm the minima and TS structures status. All obtained TS structures were confirmed by an intrinsic reaction coordinate (IRC) analysis and showed to connect the correct minima. Figure S12 shows the IRC curve for the reaction between DABCO radical cation and isovaleraldehyde together with the reactant and product minima structures.

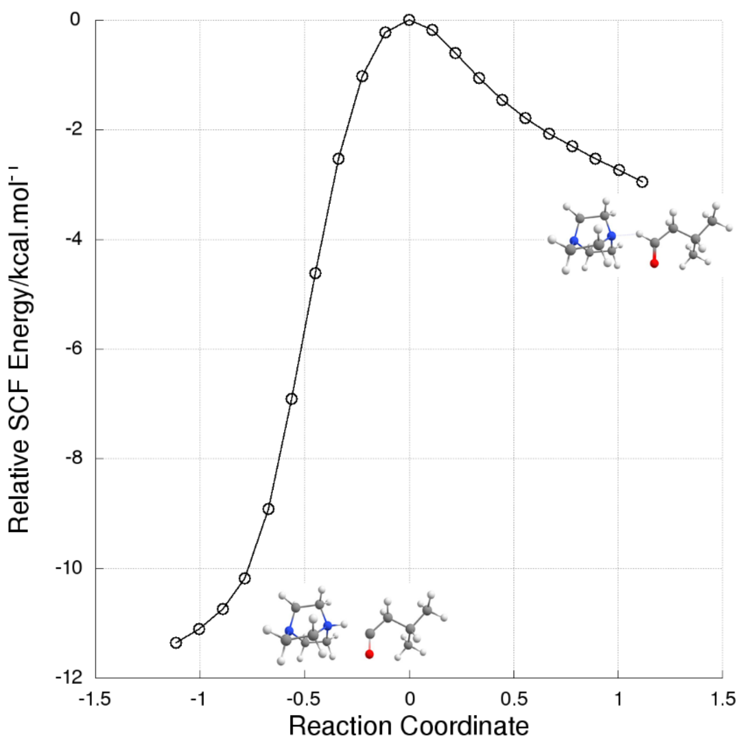

Figure S12: Intrinsic Reaction Coordinate (IRC) analysis for the TS structure of the reaction between DABCO cation radical and isovaleraldehyde at the DFT/M06-2X/cc-pVTZ level.

Potential energy surface assessments

It was not possible to find a TS structure for the reaction between quinuclidine radical cation and isovaleraldehyde. To determine if it's in fact a barrierless reaction, we investigated the reaction potential energy surface via relaxed and rigid scans and via the Nudged Elastic Band with TS optimization (NEB-TS)[20–22] approach. For the relaxed scan of the reaction involving quinuclidine radical cation, an initial structure containing a negative frequency with a normal mode compatible with the mode of reaction was first generated. (Its xyz geometry coordinate is identified as Quin_scan_ini below.) A series of structures were generated by regular displacements along this normal mode vector in both directions, and each structure was submitted to a restricted geometry optimization with the C(=O)—H and N—H distances fixed. The calculations were performed with the M06-2X functional and the 6-31G** basis set on all atoms using the Gaussian 09 suit. The resulting PES (Figure S13) shows no local maximum, in accordance with a barrierless rection.

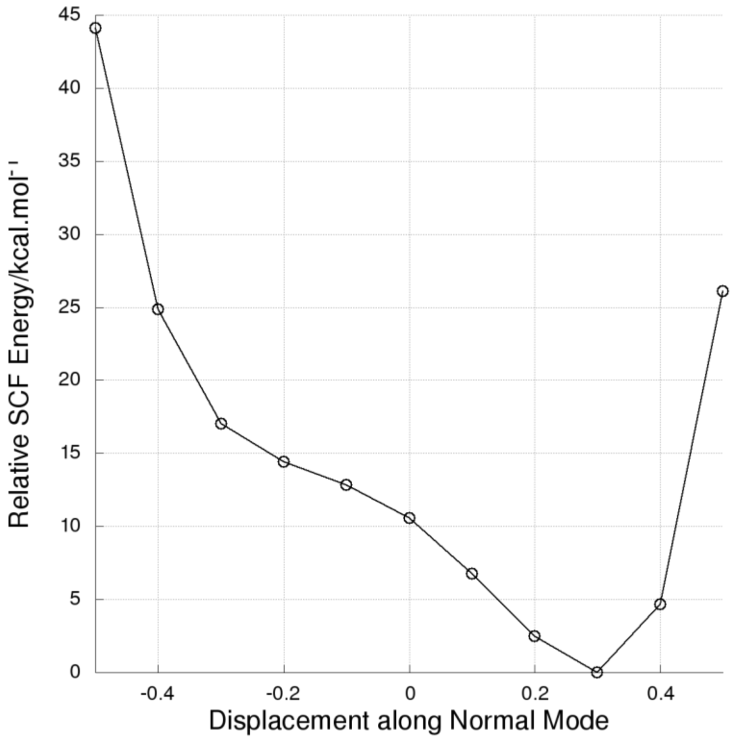

Figure S13: Relaxed PES scan for the reaction between isovaleraldehyde and quinuclidine radical cation calculated at the DFT/M06-2X/6-31G** level.

A rigid scan was also performed. For this, the geometries corresponding to the point -0.3 (reactant structure) and 0.3 (product structure) in the relaxed scan (Figure S13) were used to generate intermediate structures between them. (Their xyz geometry coordinates are identified as Quin_rgdscan_reac and Quin_rgdscan_prod, respectively, below.) For each structure, a single point calculation using the M06-2X functional and the 6-31G** basis set on all atoms were performed using the Gaussian 09 suit. The result is shown in Figure S14 and also corresponds to an essentially barrierless PES.

The Nudged Elastic Band with TS optimization (NEB-TS) approach offers a means by which an approximate TS structure can be obtained from a reactant and a product structure. A minimum energy path (MEP) connecting the two structures is obtained by the algorithm. The approximate TS is then used in a subsequent TS search to give the correct geometry. The reactant and product structures were the same used in the rigid PES scan above. The calculations were carried out using the M06-2X functional and the def2-SVP basis set on all atoms using the ORCA 4.2.1 package.[23,24] Six intermediate images were constructed. The RIJCOSX acceleration with def2/J auxiliary basis set with ThightSCF convergence, corresponding to an energy change of 1.0×10^{-8} au, were used.

Figure S15 presents the final iteration result for the reaction's MEP where, again, a downward curve with no evident barrier can be seen.

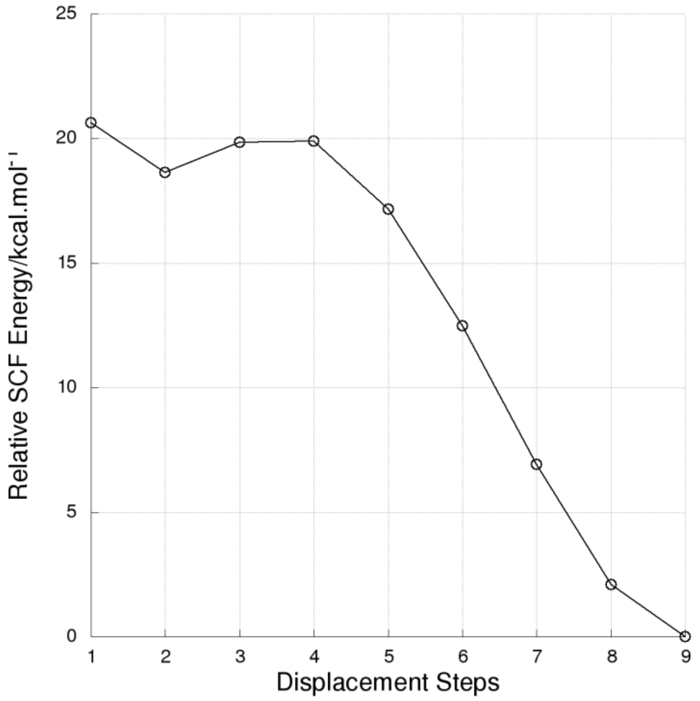

Figure S14: Rigid PES scan for the reaction between isovaleraldehyde and quinuclidine radical cation calculated at the DFT/M06-2X/6-31G** level.

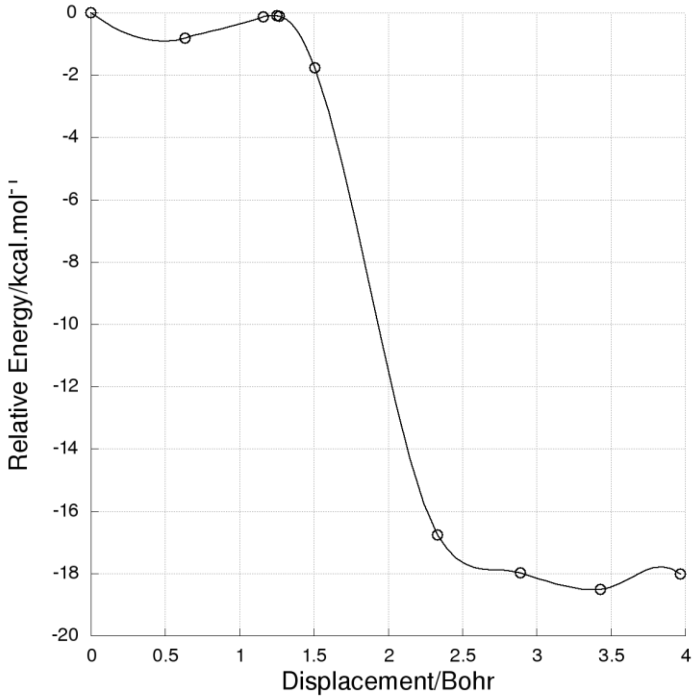

Figure S15: NEB-TS last iteration minimum energy path for the HAT step reaction between isovaleraldehyde and quinuclidine radical cation at the DFT/M06-2X/def2-SVP level.

HAT with bromine radical

To evaluate the hypothesis of a HAT step with bromine radical as abstractor, as described in the previous section, we used isovaleraldehyde again as the model aldehyde. The reactant and product structures (xyz geometry coordinates identified as RC_Br and PC_Br, respectively, below) were first obtained by optimizations as described at the beginning of this section, both in the gas phase and in 1,4-dioxane simulated with PCM. From the frequencies we obtained the thermodynamic parameters.

A regular TS search was unsuccessful. The same NEB-TS protocol described above was used to corroborate the barrierless character of this HAT step. The reactant and product structures necessary for this calculation were those obtained by optimization in the gas phase. Figure S16 shows the final iteration result for the reaction's MEP, indicating the barrierless aspect.

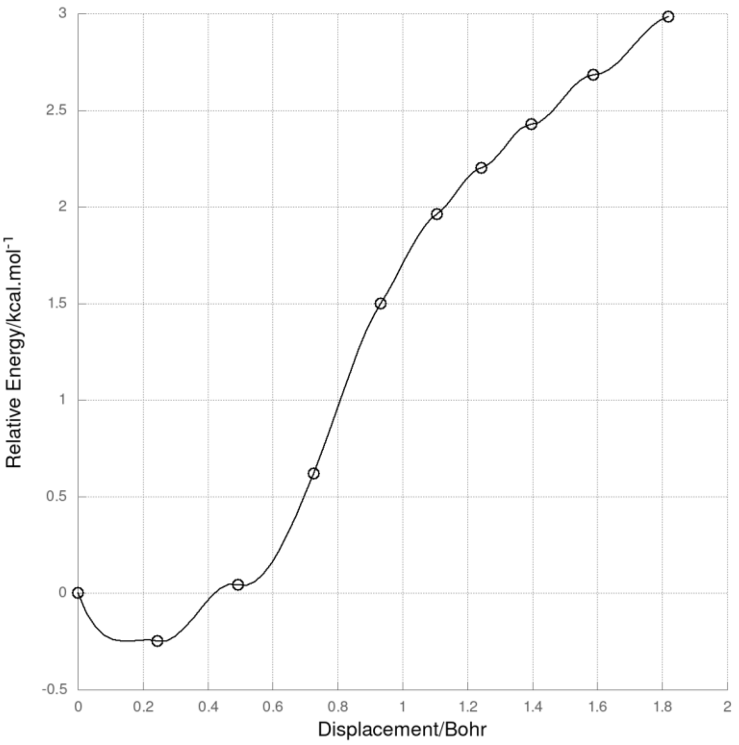

Figure S16 - NEB-TS last iteration minimum energy path for the HAT step reaction between isovaleraldehyde and bromine radical at the DFT/M06-2X/def2-SVP level.

Further calculations with DABCO

In the main text we pointed out that less detailed calculations between DABCO radical cation and the aldehydes **13** (caproic), **14** (hydrocinnamic) and **17** (pivalic) were performed. Table S7 presents the bond dissociation free energies (BDFE), the reaction

free energies (ΔG_r) and the activation free energies relative to the isolated reactant molecules ($\Delta G^{\ddagger}$) all in kcal·mol^{-1}. We also present the distension of the C(=O)—H aldehydes bonds (ΔR_{TS} C(=O)—H) at the TS, in angstrom, calculated as the difference between this bond distance in the isolated aldehyde optimized geometry and that in the respective TS structure geometry. The results show that all the reactions are mildly endergonic and present low activation energies, being feasible in the studied reaction conditions. As expected by the Evans–Polanyi principle, in general, greater reaction free energies (and greater aldehyde BDFE) corresponds to greater activation free energies.

The XYZ geometry coordinates for the aldehydes optimized geometries, their radicals and for all the TS structures are presented below.

Table S7 – Bond dissociation free energies (BDFE), reaction free energies (ΔG_r), activation free energies relative to the isolated reactant molecules ($\Delta G^{\ddagger}$) and distension of the C(=O)—H aldehydes bonds (ΔR_{TS} C(=O)—H) at the TS for the HAT reactions between DABCO radical cation and some of the aldehydes used in the experimental part of this work. The energies are in kcal·mol^{-1} and the distances are in angstrom.

**ALDEHYDE**	**BDFE**	ΔG_r	$\Delta G^{\ddagger}$	ΔR_{TS} **C(=O)—H**
Pivalic	78.96	5.69	13.90	0.13
Caproic	80.01	6.74	14.96	0.15
Isovaleric	80.06	6.79	16.73	0.14
Hydrocinnamic	80.48	7.21	14.46	0.15
$DABCO^+$–H	73.27			
$Quinuclidine^+$–H	88.03			

XYZ geometry coordinates

GAS PHASE OPTIMIZED GEOMETRIES

ALDEHYDES

Isovaleric

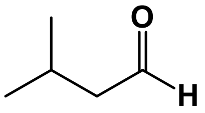

C	-2.237792	0.665986	0.267600
C	-0.992069	1.539317	0.371572
H	-3.106282	1.176258	0.684577
H	-2.111041	-0.277595	0.800000
H	-2.458266	0.435313	-0.777529
C	-1.208854	2.877324	-0.328951
H	-1.417338	2.718629	-1.390249
H	-0.333804	3.518337	-0.238091
H	-2.061546	3.403331	0.101012
C	0.213011	0.809809	-0.208883
H	-0.781133	1.735263	1.425358
C	1.545491	1.430174	0.107752
H	0.133713	0.723264	-1.299223
H	0.266344	-0.223169	0.156356
O	1.708528	2.366020	0.841605
H	2.416207	0.946739	-0.376987

Rad_Isovaleric

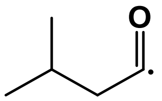

C	-2.249603	0.665080	0.256038
C	-1.005738	1.538673	0.377804
H	-3.118223	1.169110	0.680440
H	-2.123779	-0.287030	0.772594
H	-2.467560	0.454206	-0.793530
C	-1.212516	2.880557	-0.315998
H	-1.409628	2.729747	-1.380157
H	-0.338327	3.521601	-0.214032
H	-2.068222	3.404743	0.109855
C	0.200985	0.798966	-0.203637
H	-0.800748	1.721563	1.435340
C	1.537987	1.451282	0.080370
H	0.118271	0.686818	-1.287887
H	0.285173	-0.215179	0.196556
O	1.760617	2.381343	0.763913

Pivalic

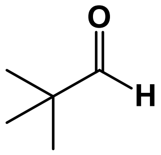

C	-3.976103	1.397419	0.083077
C	-2.465466	1.367511	-0.001054
O	-4.667620	2.341932	-0.174713
C	-1.942270	1.011911	1.394592
C	-1.933539	2.715531	-0.462042
C	-2.090003	0.256084	-0.986909
H	-4.431916	0.440533	0.415869
H	-0.856930	0.907852	1.364949
H	-2.360667	0.066823	1.745922
H	-2.192076	1.789070	2.117671
H	-2.447548	0.484828	-1.991430
H	-2.510710	-0.703812	-0.681434
H	-1.005175	0.149832	-1.025498
H	-2.331813	2.972679	-1.442918
H	-0.844680	2.689225	-0.521212
H	-2.227873	3.503893	0.229919

Rad_Pivalic

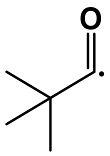

C	-3.966995	1.563277	-0.009221
C	-2.439134	1.396108	-0.003714
O	-4.695163	1.595532	0.909786

C	-1.957457	1.133879	1.420221
C	-1.859291	2.698368	-0.557076
C	-2.103561	0.230941	-0.932209
H	-0.872293	1.019649	1.425822
H	-2.403932	0.223931	1.820148
H	-2.225049	1.959454	2.078603
H	-2.488874	0.411502	-1.935455
H	-2.528053	-0.702526	-0.561923
H	-1.020439	0.114608	-0.990121
H	-2.242334	2.900511	-1.557152
H	-0.773202	2.611565	-0.611459
H	-2.104503	3.544043	0.085670

Caproic

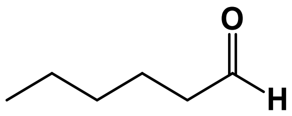

C	-2.969775	1.761904	0.378463
C	-1.575263	1.256393	0.140496
O	-3.240507	2.696273	1.081131
H	-3.772216	1.208462	-0.147430
C	-0.502502	2.038359	0.876587
H	-1.407890	1.265822	-0.942761
H	-1.563091	0.196410	0.419485
C	0.897639	1.500777	0.614076
H	-0.716689	2.017522	1.947335
H	-0.561244	3.088652	0.582909
C	1.980581	2.279865	1.349074
H	1.103298	1.524380	-0.461256
H	0.947042	0.447675	0.910264
C	3.377513	1.735818	1.081118
H	1.773736	2.255167	2.422131
H	1.929808	3.330640	1.052189
H	3.455678	0.694120	1.396286
H	4.138956	2.304260	1.614125
H	3.612876	1.777343	0.016479

Rad_Caproic

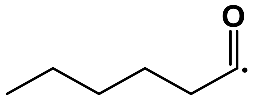

C	-2.971339	1.758969	0.375989
C	-1.564968	1.249606	0.136345
O	-3.299449	2.664225	1.049262
C	-0.494548	2.040571	0.879232
H	-1.410027	1.265937	-0.945097
H	-1.565530	0.194417	0.419834
C	0.904790	1.501186	0.615212
H	-0.709972	2.014838	1.949527
H	-0.554036	3.089371	0.580764
C	1.987833	2.280740	1.350584
H	1.108987	1.525521	-0.460103
H	0.952679	0.448439	0.911897
C	3.384192	1.735665	1.081754
H	1.781213	2.255625	2.423611
H	1.937322	3.331359	1.053333
H	3.462054	0.694086	1.397083
H	4.145544	2.304100	1.614742
H	3.619264	1.777404	0.017151

Hydrocinnamic

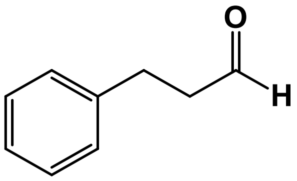

C	-2.163412	1.400650	0.536251
C	-0.684819	1.383042	0.265422
O	-2.651261	1.431420	1.631913
H	-2.809494	1.383098	-0.362204
H	4.464453	1.418842	3.222733
C	3.846700	1.401207	2.334844
C	2.468390	1.414400	2.455194
H	2.014014	1.442340	3.438624
C	1.644000	1.392356	1.329635
C	2.245852	1.356609	0.077182
H	1.640528	1.338688	-0.819101
C	3.630914	1.343213	-0.049209
H	4.076835	1.315297	-1.034446
C	4.436024	1.365397	1.076456
H	5.512778	1.354978	0.977922
C	0.147645	1.408475	1.532553

H	-0.464624	2.236461	-0.384836
H	-0.477459	0.495138	-0.341442
H	-0.139090	0.559412	2.156562
H	-0.126272	2.291716	2.113387

Rad_Hydrocinnamic

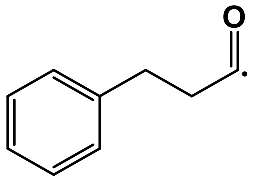

C	-2.153540	1.555490	0.434383
C	-0.659068	1.510664	0.191549
O	-2.712965	1.561673	1.467196
H	4.424382	1.313470	3.255340
C	3.823548	1.342930	2.356285
C	2.443928	1.385950	2.451880
H	1.971741	1.389917	3.427255
C	1.642618	1.424589	1.310410
C	2.266207	1.418823	0.068351
H	1.678681	1.447969	-0.839425
C	3.652650	1.375639	-0.032352
H	4.116759	1.371946	-1.009441
C	4.435656	1.337588	1.108388
H	5.513510	1.304024	1.029376
C	0.142787	1.470531	1.485620
H	-0.418327	2.383039	-0.419425
H	-0.473824	0.638746	-0.439113
H	-0.177371	0.604195	2.068223
H	-0.121862	2.342216	2.087779

CATALYSTS

CatRad_DABCO

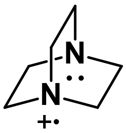

H	0.560084	-0.194666	-1.541010
C	0.449842	0.040026	-0.485990
H	1.247486	-0.419740	0.090741
C	-0.990584	-0.526368	0.042945
H	-1.508504	-1.009399	-0.780894
H	-0.821340	-1.232257	0.851238
N	-1.724501	0.605999	0.507257
C	-1.101270	1.232837	1.627622
H	-1.701333	2.064800	1.985434
H	-0.932667	0.514988	2.425412
C	0.339867	1.798247	1.099835
H	0.368746	2.876598	1.228653
H	1.137720	1.326478	1.666516
N	0.428605	1.452228	-0.281771
C	-2.013082	1.531341	-0.539987
C	-0.572132	2.098420	-1.067403
H	-2.607808	2.360549	-0.167338
H	-2.523853	1.035783	-1.360819
H	-0.538113	3.174682	-0.923639
H	-0.454503	1.851364	-2.118741

Cat_H-DABCO

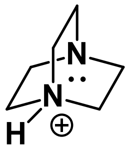

H	0.689604	-0.359774	-1.460851
C	0.416040	-0.081021	-0.445782
H	1.177158	-0.446101	0.240913
C	-1.015754	-0.524985	-0.052995
H	-1.557947	-0.887472	-0.924405
H	-0.967037	-1.334796	0.671734
N	-1.750301	0.596689	0.518819
C	-0.987733	1.183178	1.613620
H	-1.602297	1.914775	2.133626
H	-0.733529	0.396024	2.321056
C	0.293603	1.862455	1.068505
H	0.227828	2.948645	1.058243
H	1.195972	1.565623	1.598089
N	0.441489	1.422456	-0.364043
C	-1.999937	1.601173	-0.506995
C	-0.671394	2.001787	-1.197282
H	-2.466171	2.466907	-0.040150
H	-2.694862	1.203179	-1.243217
H	-0.519494	3.077618	-1.246199
H	-0.568150	1.581530	-2.195704

H	1.333052	1.758380	-0.723252

CatRad_Quin

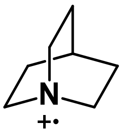

H	0.734803	-0.391284	-1.403577
C	0.488877	-0.040650	-0.403529
H	1.190013	-0.486549	0.299489
C	-0.980104	-0.527280	-0.056543
H	-1.511728	-0.860642	-0.943635
H	-0.967302	-1.313404	0.694402
N	-1.630454	0.643373	0.472855
C	-1.021368	1.181514	1.661728
H	-1.697713	1.888905	2.135346
H	-0.788523	0.367053	2.342142
C	0.291560	1.902352	1.141740
H	0.166538	2.980284	1.225722
H	1.118969	1.601472	1.780783
C	0.511600	1.484820	-0.311950
C	-1.993748	1.626775	-0.514469
C	-0.615921	2.054709	-1.172286
H	-2.480060	2.464218	-0.021765
H	-2.659824	1.181990	-1.249744
H	-0.583672	3.141274	-1.215968
H	-0.568172	1.668110	-2.188594
H	1.472349	1.861979	-0.663958

Cat_H-Quin

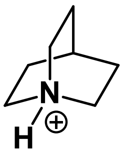

H	0.033764	-0.014304	-0.095236
C	1.119429	0.006486	-0.026861
H	1.379907	0.038336	1.030574
C	1.646322	1.256647	-0.747941
H	0.934994	1.657128	-1.467656
H	1.938768	2.050292	-0.064545
N	2.871364	0.865123	-1.533927
C	3.832332	0.111824	-0.650301
H	4.783973	0.060240	-1.173905
H	3.958525	0.712030	0.248813
C	3.231062	-1.271801	-0.359460
H	3.726953	-2.035978	-0.957033
H	3.393983	-1.519811	0.687321
C	1.734144	-1.232756	-0.686718
C	2.477683	0.012580	-2.713131
C	1.565965	-1.115116	-2.205493
H	3.404232	-0.359770	-3.146079
H	1.989432	0.662265	-3.435632
H	1.838250	-2.045263	-2.699844
H	0.526044	-0.905184	-2.453720
H	1.246214	-2.133054	-0.323295
H	3.328779	1.708847	-1.874753

TRANSITION STATES WITH DABCO

TS_Isovaleric

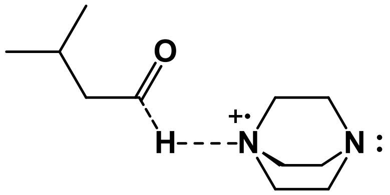

H	1.059606	0.100881	1.066929
C	0.467391	0.973496	0.799902
H	0.874656	1.838622	1.319174
C	-1.033875	0.748266	1.218911
H	-1.216696	-0.273342	1.549301
H	-1.340319	1.439872	2.001659
N	-1.853384	0.992966	0.026469
C	-1.644649	2.349328	-0.488695
H	-2.343560	2.529388	-1.302876
H	-1.839011	3.056772	0.314883
C	-0.147436	2.419834	-0.980466
H	-0.115994	2.551964	-2.060071
H	0.351083	3.267881	-0.515629
N	0.547286	1.196544	-0.631116
C	-1.578733	-0.013735	-1.003877
C	-0.041871	0.072394	-1.333948
H	-2.191382	0.214234	-1.874267
H	-1.859020	-0.991039	-0.612525

H	0.096347	0.197743	-2.405759
H	0.460584	-0.842376	-1.026836
H	-5.286259	1.655366	1.651471
H	-6.097153	0.466642	3.771203
H	-7.833146	1.607437	2.230646
C	-5.505381	0.749672	1.076542
C	-6.212858	-0.443720	3.178868
H	-3.250539	0.722085	0.447375
C	-7.909601	0.665141	1.684471
C	-4.203485	0.028661	0.868855
H	-5.287181	-1.015881	3.231992
H	-5.803587	1.093502	0.079334
C	-6.590320	-0.099673	1.742862
H	-6.996905	-1.041165	3.641281
H	-8.703037	0.078064	2.144744
H	-8.203968	0.885817	0.658549
O	-3.925384	-1.106726	1.023751
H	-6.687596	-1.023756	1.168924

TS_Pivalic

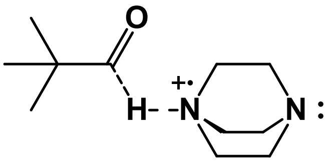

H	0.304576	-0.940282	-0.906569
C	0.217612	-0.212457	-0.102488
H	0.915422	-0.485619	0.685912
C	-1.251006	-0.204346	0.465068
H	-1.857772	-0.992464	0.023299
H	-1.267965	-0.303971	1.549239
N	-1.836038	1.094634	0.117554
C	-1.150342	2.180606	0.823849
H	-1.595982	3.123208	0.510908
H	-1.305527	2.053532	1.894527
C	0.371582	2.094323	0.429716
H	0.713532	3.064813	0.076915
H	0.969395	1.812024	1.293606
N	0.550328	1.103155	-0.614835
C	-1.814529	1.311588	-1.331071
C	-0.297160	1.418844	-1.748292
H	-2.358089	2.226029	-1.559849

H	-2.307938	0.469764	-1.813231
H	-0.074426	2.427969	-2.088735
H	-0.091501	0.727416	-2.562478
H	-3.223403	1.119693	0.697151
C	-4.115840	1.232841	1.552879
O	-3.758105	1.624607	2.603630
H	-5.091570	-1.300400	1.296761
C	-5.375501	-0.595580	0.515573
H	-6.341801	-0.902950	0.116729
H	-4.648048	-0.651208	-0.296849
H	-6.208605	0.243579	3.025405
C	-5.499663	0.829637	1.060134
C	-6.488582	0.917238	2.217105
H	-7.477845	0.634992	1.857186
C	-5.851260	1.804539	-0.068732
H	-6.539128	1.929719	2.613930
H	-6.819885	1.522114	-0.480580
H	-5.119921	1.763134	-0.877897
H	-5.921527	2.828928	0.295198

TS_Capric

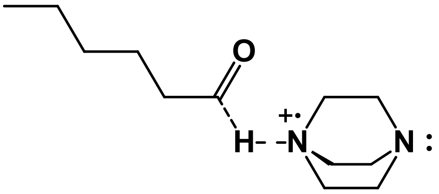

H	1.059606	0.100881	1.066929
C	0.467391	0.973496	0.799902
H	0.874656	1.838622	1.319174
C	-1.033875	0.748266	1.218911
H	-1.216696	-0.273342	1.549301
H	-1.340319	1.439872	2.001659
N	-1.853384	0.992966	0.026469
C	-1.644649	2.349328	-0.488695
H	-2.343560	2.529388	-1.302876
H	-1.839011	3.056772	0.314883
C	-0.147436	2.419834	-0.980466
H	-0.115994	2.551964	-2.060071
H	0.351083	3.267881	-0.515629
N	0.547286	1.196544	-0.631116
C	-1.578733	-0.013735	-1.003877

C	-0.041871	0.072394	-1.333948
H	-2.191382	0.214234	-1.874267
H	-1.859020	-0.991039	-0.612525
H	0.096347	0.197743	-2.405759
H	0.460584	-0.842376	-1.026836
H	-5.286259	1.655366	1.651471
H	-6.097153	0.466642	3.771203
H	-7.833146	1.607437	2.230646
C	-5.505381	0.749672	1.076542
C	-6.212858	-0.443720	3.178868
H	-3.250539	0.722085	0.447375
C	-7.909601	0.665141	1.684471
C	-4.203485	0.028661	0.868855
H	-5.287181	-1.015881	3.231992
H	-5.803587	1.093502	0.079334
C	-6.590320	-0.099673	1.742862
H	-6.996905	-1.041165	3.641281
H	-8.703037	0.078064	2.144744
H	-8.203968	0.885817	0.658549
O	-3.925384	-1.106726	1.023751
H	-6.687596	-1.023756	1.168924

TS_hydrocinnamic

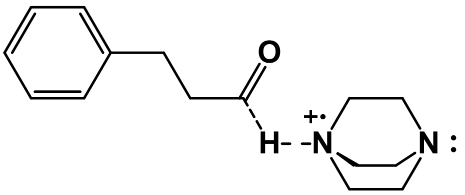

H	0.449350	0.960129	1.910476
C	-0.264401	1.535466	1.325165
H	-0.329512	2.535705	1.748498
C	-1.673633	0.836315	1.366213
H	-1.622708	-0.156570	1.810668
H	-2.405119	1.434429	1.906723
N	-2.119004	0.688816	-0.024330
C	-2.172217	1.988402	-0.700266
H	-2.619366	1.858545	-1.683511
H	-2.792644	2.658611	-0.108581
C	-0.685463	2.503856	-0.801288
H	-0.365617	2.518854	-1.841157
H	-0.621692	3.515621	-0.406859
N	0.193548	1.630294	-0.048192
C	-1.254805	-0.248988	-0.749021
C	0.213115	0.309341	-0.648606
H	-1.598485	-0.298363	-1.780623
H	-1.351321	-1.233545	-0.292846
H	0.651690	0.364361	-1.642541
H	0.826713	-0.349415	-0.037920
H	-3.428309	-0.005003	0.038092
C	-4.226999	-0.947977	0.237824
O	-3.708857	-1.991559	0.411171
H	-6.265751	-1.455974	0.494370
H	-5.598480	0.169939	2.316728
C	-5.680983	-0.560729	0.284524
C	-8.238613	0.321569	2.251706
C	-9.585278	0.654816	2.247017
C	-7.359796	0.934040	1.365146
C	-5.906638	0.537149	1.337067
C	-10.063427	1.602518	1.353841
C	-7.846508	1.883376	0.471974
C	-9.191762	2.218173	0.465991
H	-7.867775	-0.414390	2.955013
H	-10.259742	0.177281	2.943915
H	-11.111990	1.864835	1.352366
H	-7.167775	2.372647	-0.217618
H	-9.559218	2.962548	-0.226559
H	-5.286040	1.403988	1.095203
H	-5.948849	-0.181011	-0.704205

REACTANTS AND PRODUCTS COMPLEXES

RC_DABCO

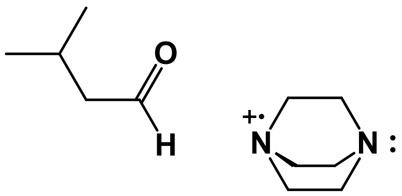

H	-2.765953	-2.206869	-0.811167
C	-3.042676	-1.173267	-1.001017
H	-3.638064	-1.107656	-1.909758
C	-1.690590	-0.278953	-1.191964
H	-0.805943	-0.894685	-1.047796

H	-1.692520	0.158053	-2.188466
N	-1.741144	0.741126	-0.199165
C	-2.852449	1.616287	-0.377929
H	-2.837184	2.424100	0.354688
H	-2.860955	2.023280	-1.386690
C	-4.201435	0.705632	-0.160333
H	-4.771016	1.094971	0.679102
H	-4.789909	0.732795	-1.074450
N	-3.753376	-0.619586	0.102209
C	-1.671618	0.201898	1.120049
C	-3.014850	-0.720595	1.316055
H	-1.666534	0.999871	1.863672
H	-0.785208	-0.417799	1.224948
H	-3.593430	-0.347673	2.156872
H	-2.714826	-1.750442	1.491963
H	3.648817	1.356227	-1.215161
H	4.568868	-0.914536	-1.665353
H	6.008198	0.783833	-0.305651
C	3.312624	1.050385	-0.216482
C	4.145533	-1.251605	-0.715963
H	1.263621	1.680217	-0.723600
C	5.559541	0.358144	0.593328
C	1.837118	0.832037	-0.300285
H	3.133780	-1.608544	-0.901490
H	3.438755	1.946296	0.404076
C	4.143262	-0.116118	0.301188
H	4.740989	-2.093314	-0.369379
H	6.189928	-0.466978	0.919518
H	5.583117	1.128537	1.366457
O	1.249078	-0.157915	0.064484
H	3.684436	-0.477126	1.225056

RP_DABCO

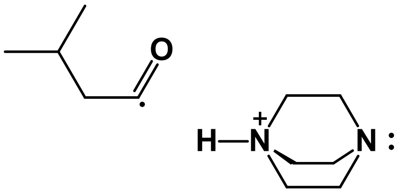

H	-3.555527	-2.099185	-0.971733
C	-3.322140	-1.038376	-1.030161
H	-3.774780	-0.635634	-1.935456
C	-1.788294	-0.830104	-1.062673
H	-1.237159	-1.661519	-0.630472
H	-1.404359	-0.622467	-2.059950
N	-1.496491	0.367480	-0.205062
C	-2.415065	1.494542	-0.584439
H	-2.045655	2.403181	-0.114933
H	-2.363173	1.599596	-1.666265
C	-3.834774	1.094705	-0.110691
H	-4.110751	1.610972	0.811009
H	-4.559406	1.380400	-0.870555
N	-3.890294	-0.340973	0.121747
C	-1.724232	0.003054	1.236458
C	-3.118458	-0.670498	1.308383
H	-1.685306	0.929252	1.806740
H	-0.911333	-0.649909	1.544658
H	-3.645927	-0.337274	2.199503
H	-3.021779	-1.755590	1.366136
H	3.476530	1.421090	-1.262202
H	4.605456	-0.807697	-1.686278
H	5.947755	1.061071	-0.481924
C	3.226186	1.097622	-0.248014
C	4.261362	-1.139657	-0.704537
H	-0.517711	0.639592	-0.326810
C	5.562290	0.678885	0.465693
C	1.791348	0.668620	-0.268972
H	3.282302	-1.602437	-0.827444
H	3.243884	2.004174	0.363330
C	4.198067	0.028084	0.268134
H	4.954818	-1.901883	-0.354340
H	6.282469	-0.041940	0.848019
H	5.516125	1.514594	1.166371
O	1.284826	-0.351350	0.053647
H	3.825179	-0.322392	1.234652

RC_Quin

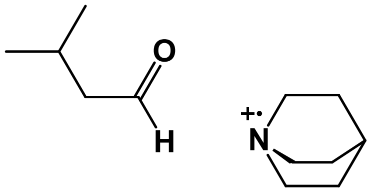

H	3.269059	-0.215111	-2.273469

C	2.537160	-0.523491	-1.530056
H	1.933433	-1.322394	-1.956712
C	1.611246	0.710702	-1.192704
H	2.035426	1.644429	-1.551605
H	0.601797	0.584165	-1.572617
N	1.565739	0.740531	0.246621
C	1.002511	-0.440992	0.849083
H	0.759800	-0.236670	1.889107
H	0.101110	-0.708204	0.305915
C	2.139704	-1.528117	0.716166
H	2.562681	-1.736449	1.697346
H	1.695929	-2.445223	0.334877
C	3.209289	-0.981409	-0.233082
C	2.762526	1.231352	0.880024
C	3.890335	0.220652	0.425230
H	2.622908	1.221300	1.957402
H	2.970221	2.241604	0.536947
H	4.464380	-0.071553	1.301847
H	4.561129	0.718430	-0.272641
H	-4.070020	1.718851	0.764674
H	-3.766427	-0.507306	1.981145
H	-5.501236	-0.426217	0.105667
C	-3.428604	1.376658	-0.055794
C	-2.985371	-0.721797	1.248945
H	-2.152680	3.160179	0.234306
C	-4.702470	-0.711810	-0.581544
C	-2.110317	2.068254	0.080805
H	-2.052418	-0.297913	1.621650
H	-3.888842	1.794700	-0.960172
C	-3.366702	-0.144886	-0.112547
H	-2.875794	-1.804994	1.195135
H	-4.670651	-1.800198	-0.618696
H	-4.965896	-0.346414	-1.574052
O	-1.034829	1.518805	0.012083
H	-2.595640	-0.418400	-0.838286
H	3.947220	-1.753631	-0.447823

RP_Quin

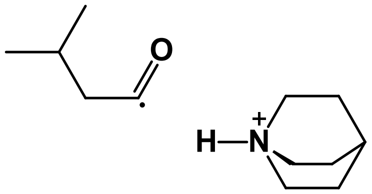

H	-3.558211	-0.730559	2.149659
C	-3.069832	-0.879442	1.190147
H	-2.923915	-1.951553	1.056711
C	-1.716981	-0.152159	1.190508
H	-1.702897	0.685753	1.887388
H	-0.877927	-0.809883	1.407754
N	-1.491897	0.423114	-0.179380
C	-1.782534	-0.630004	-1.207410
H	-1.354329	-0.297636	-2.150117
H	-1.258875	-1.526919	-0.887134
C	-3.306892	-0.814413	-1.277922
H	-3.710710	-0.250208	-2.118801
H	-3.533578	-1.864630	-1.442756
C	-3.908783	-0.315402	0.040916
C	-2.397330	1.601400	-0.388238
C	-3.808974	1.210169	0.074396
H	-2.364696	1.840125	-1.450055
H	-1.989822	2.440962	0.173120
H	-4.530858	1.671744	-0.596547
H	-4.018261	1.592517	1.074171
H	3.461415	1.680953	-0.887097
H	4.602555	-0.361793	-1.860676
H	5.940932	1.146156	-0.221491
C	3.215799	1.110020	0.012587
C	4.263749	-0.933097	-0.993923
H	-0.512482	0.708685	-0.258386
C	5.556161	0.536217	0.598565
C	1.784270	0.686656	-0.116073
H	3.288386	-1.359170	-1.229130
H	3.227997	1.832636	0.833288
C	4.194552	-0.048746	0.241967
H	4.965678	-1.751725	-0.847680
H	6.278717	-0.255248	0.787632
H	5.506995	1.168046	1.487379
O	1.287429	-0.385236	-0.053286
H	3.824071	-0.633754	1.088380

H	-4.944212	-0.634716	0.134241

RC_Br

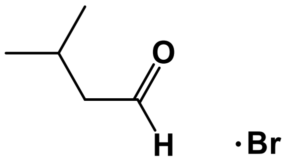

Br	-2.399616	2.120109	-0.161152
H	-5.187310	1.460951	1.706404
H	-6.090862	0.232626	3.754172
H	-7.775374	1.475606	2.248333
C	-5.456628	0.602208	1.081753
C	-6.218740	-0.650976	3.124641
H	-3.333811	0.591613	0.403564
C	-7.874747	0.554172	1.670456
C	-4.182999	-0.146072	0.817883
H	-5.301007	-1.236082	3.160771
H	-5.739179	1.029767	0.113311
C	-6.570084	-0.236269	1.699630
H	-7.018373	-1.253268	3.554953
H	-8.684325	-0.028766	2.108986
H	-8.160677	0.822245	0.653130
O	-3.944869	-1.293921	0.944809
H	-6.687089	-1.136601	1.091972

PC_Br

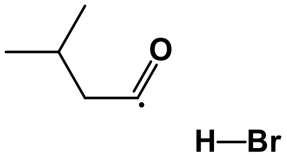

Br	-2.488744	-0.478904	0.628437
H	0.672914	-0.732894	0.570268
H	2.682903	-0.004987	1.897593
H	2.895131	-2.126135	0.501206
C	1.081954	-0.272794	-0.332476
C	2.989345	0.591949	1.038243
H	-1.605356	0.450998	-0.002587
C	3.252393	-1.497432	-0.315370
C	0.343321	1.029860	-0.544422
H	2.518566	1.570390	1.112118
H	0.765537	-0.913709	-1.160012
C	2.600157	-0.120421	-0.252757
H	4.068418	0.728614	1.095644
H	4.334390	-1.410964	-0.215625
H	3.040561	-2.011679	-1.252893
O	0.762639	2.093207	-0.804888
H	2.926100	0.477612	-1.104791

PCM OPTIMIZED GEOMETRIES

Isovaleric

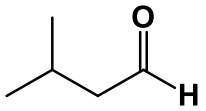

C	-2.236723	0.666000	0.267690
C	-0.992232	1.541003	0.371787
H	-3.106070	1.176252	0.682986
H	-2.108318	-0.276270	0.801843
H	-2.454591	0.433547	-0.777499
C	-1.211827	2.878003	-0.330029
H	-1.423979	2.716543	-1.390035
H	-0.336391	3.519704	-0.245397
H	-2.062552	3.404949	0.102721
C	0.213288	0.812607	-0.208594
H	-0.783708	1.736643	1.426075
C	1.546131	1.426589	0.108723
H	0.136322	0.727538	-1.299006
H	0.267002	-0.221054	0.154505
O	1.715184	2.358243	0.850271
H	2.413633	0.944702	-0.380120

Rad_Isovaleric

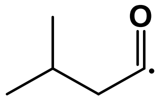

C	-2.248874	0.664919	0.255547
C	-1.006067	1.539944	0.378094
H	-3.117729	1.169572	0.678691
H	-2.121854	-0.286134	0.773537
H	-2.464626	0.453108	-0.794213

C	-1.214363	2.881226	-0.316404
H	-1.414055	2.728803	-1.379713
H	-0.339947	3.522932	-0.218447
H	-2.068687	3.405569	0.111962
C	0.200541	0.800015	-0.203904
H	-0.802419	1.721821	1.436011
C	1.535388	1.450004	0.080014
H	0.118286	0.687463	-1.288071
H	0.285609	-0.214021	0.196572
O	1.767486	2.376260	0.767992

CatRad_DABCO

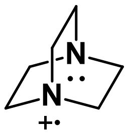

H	0.631806	-0.223304	-1.493028
C	0.464571	0.040108	-0.453158
H	1.229641	-0.396998	0.181287
C	-0.999634	-0.521993	0.004679
H	-1.508914	-0.943991	-0.857331
H	-0.862758	-1.274169	0.776352
N	-1.720407	0.600734	0.510334
C	-1.075726	1.192294	1.637635
H	-1.684950	1.984382	2.062927
H	-0.850549	0.437274	2.384923
C	0.319089	1.837530	1.086466
H	0.273928	2.918770	1.175745
H	1.144839	1.438072	1.667837
N	0.425718	1.456836	-0.284829
C	-2.016018	1.555854	-0.508034
C	-0.579176	2.070875	-1.090294
H	-2.559230	2.399536	-0.092702
H	-2.576496	1.097908	-1.316976
H	-0.532573	3.152746	-1.007559
H	-0.480500	1.759446	-2.126214

Cat_H-DABCO

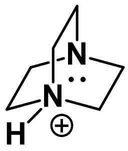

H	0.711180	-0.365029	-1.449105
C	0.417286	-0.073039	-0.443800
H	1.164401	-0.425279	0.263777
C	-1.017565	-0.516904	-0.072311
H	-1.556694	-0.850808	-0.956289
H	-0.976169	-1.344915	0.630701
N	-1.752272	0.594318	0.523052
C	-0.970671	1.174445	1.610260
H	-1.583113	1.887550	2.156668
H	-0.686899	0.377990	2.296125
C	0.284559	1.882769	1.048091
H	0.183923	2.964871	1.025246
H	1.198001	1.619035	1.575056
N	0.432435	1.429959	-0.379858
C	-2.006793	1.609981	-0.493583
C	-0.689626	1.988502	-1.211317
H	-2.444674	2.481430	-0.009932
H	-2.724415	1.229416	-1.217539
H	-0.538618	3.061458	-1.290756
H	-0.601411	1.539729	-2.198803
H	1.303573	1.764069	-0.740293

CatRad_Quin

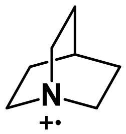

H	0.732016	-0.388691	-1.404840
C	0.488182	-0.040767	-0.403692
H	1.187877	-0.488013	0.299654
C	-0.979444	-0.527003	-0.058218
H	-1.510555	-0.858522	-0.945919
H	-0.964797	-1.311993	0.693308
N	-1.629929	0.642263	0.472611
C	-1.021537	1.179551	1.661568
H	-1.698660	1.886776	2.132944
H	-0.785798	0.365564	2.340929

C	0.288483	1.903282	1.141428
H	0.154830	2.980311	1.221967
H	1.116767	1.606808	1.780970
C	0.511655	1.484187	-0.311309
C	-1.990858	1.628698	-0.512222
C	-0.614920	2.052300	-1.172358
H	-2.468797	2.467798	-0.015431
H	-2.659863	1.188133	-1.246621
H	-0.582556	3.138528	-1.218562
H	-0.568331	1.658266	-2.185535
H	1.472356	1.861546	-0.662482

Cat_H-Quin

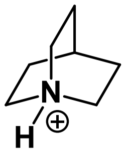

H	0.033515	-0.014600	-0.103038
C	1.118549	0.006878	-0.029770
H	1.378682	0.038360	1.026886
C	1.648559	1.257215	-0.747226
H	0.938093	1.660764	-1.465178
H	1.941684	2.047337	-0.061087
N	2.870459	0.864132	-1.532476
C	3.831126	0.111638	-0.652644
H	4.779095	0.057964	-1.181440
H	3.961625	0.711372	0.246016
C	3.229899	-1.271150	-0.358703
H	3.722785	-2.034601	-0.959076
H	3.391170	-1.517063	0.688305
C	1.733543	-1.232470	-0.685733
C	2.476978	0.013922	-2.710514
C	1.568570	-1.117029	-2.204554
H	3.403912	-0.355121	-3.144591
H	1.985568	0.664455	-3.429440
H	1.846662	-2.047218	-2.695295
H	0.528600	-0.910514	-2.453630
H	1.244965	-2.131923	-0.321469
H	3.328078	1.706411	-1.874163

RC_Br

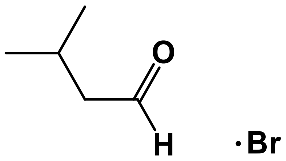

Br	-2.349932	2.041679	-0.257753
H	-5.200925	1.471595	1.729139
H	-6.116321	0.213485	3.766315
H	-7.785284	1.466492	2.261555
C	-5.461525	0.615822	1.097033
C	-6.229138	-0.663524	3.125162
H	-3.331979	0.642691	0.401735
C	-7.879118	0.551947	1.672199
C	-4.182313	-0.122307	0.838618
H	-5.305598	-1.239537	3.163583
H	-5.738597	1.044337	0.127896
C	-6.572987	-0.236296	1.702418
H	-7.026685	-1.279593	3.539364
H	-8.689011	-0.039393	2.098738
H	-8.158163	0.829406	0.655655
O	-3.914048	-1.260334	0.969090
H	-6.684066	-1.129129	1.082869

PC_Br

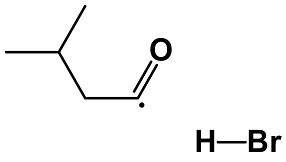

Br	-2.474240	-0.468354	0.626375
H	0.675589	-0.739523	0.570981
H	2.679517	-0.006096	1.896877
H	2.890468	-2.125132	0.499776
C	1.080545	-0.275842	-0.331615
C	2.987176	0.592046	1.038896
H	-1.577242	0.454678	-0.003794
C	3.250640	-1.496395	-0.315480
C	0.338202	1.023173	-0.539691
H	2.516667	1.570647	1.114576
H	0.765116	-0.914362	-1.161491
C	2.598559	-0.119056	-0.253002
H	4.066386	0.727490	1.096140
H	4.332257	-1.409540	-0.212446

H	3.040325	-2.008735	-1.254278
O	0.746072	2.089627	-0.808513
H	2.924193	0.478086	-1.105623

OTHERS

Quin_rlxdscan_ini

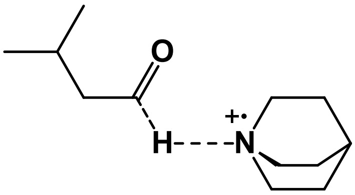

H	-3.81706000	-0.44334800	1.96005500
C	-3.26155200	-0.75029200	1.07096300
H	-3.43828900	-1.81985300	0.92715900
C	-1.75291700	-0.47488700	1.28255200
H	-1.57324800	0.23405900	2.09504700
H	-1.18196100	-1.38519900	1.48584600
N	-1.20495000	0.13738300	0.04288500
C	-1.54310000	-0.69682600	-1.14095100
H	-0.94410800	-0.34456800	-1.98441100
H	-1.23945100	-1.72047700	-0.90561400
C	-3.06339600	-0.58030100	-1.40917900
H	-3.24879700	0.04731700	-2.28547300
H	-3.47523200	-1.56967100	-1.62105400
C	-3.72485300	0.03416200	-0.16655600
C	-1.71131000	1.52404800	-0.12609900
C	-3.25777500	1.49225700	-0.03062700
H	-1.36454100	1.87839500	-1.09998600
H	-1.25975300	2.14708300	0.64945100
H	-3.68594800	2.11379000	-0.82045000
H	-3.58854300	1.90491000	0.92659900
H	2.34517000	1.08500700	-0.88165500
H	4.08735700	-0.54088200	-1.88304200
H	4.89037100	1.70067700	-0.87248900
C	2.54569400	0.65553500	0.10866100
C	4.18451700	-0.99187900	-0.88952400
H	0.15661500	0.09576800	0.17650200
C	4.97277200	1.19473800	0.09520500
C	1.44909800	-0.34096700	0.39369500
H	3.48116000	-1.82530200	-0.81270400
H	2.39108300	1.46733100	0.83289100
C	3.95790800	0.05484600	0.19924200
H	5.19245900	-1.40471900	-0.80971800
H	5.98739500	0.79841500	0.17808000
H	4.83504000	1.93747400	0.88558700
O	1.48422400	-1.45147500	0.78865300
H	4.05275000	-0.42131500	1.18217300
H	-4.81211500	-0.00736900	-0.25650500

Quin_rgdscan_reac

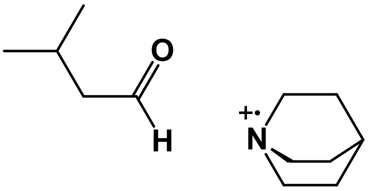

H	-3.671287	-0.447192	2.036626
C	-3.111130	-0.758131	1.151898
H	-3.138237	-1.849617	1.104824
C	-1.630477	-0.263703	1.291501
H	-1.526214	0.494970	2.070139
H	-0.938745	-1.088452	1.485955
N	-1.265631	0.348134	0.014869
C	-1.427767	-0.574440	-1.110671
H	-0.937883	-0.146762	-1.988462
H	-0.936978	-1.513128	-0.843421
C	-2.968377	-0.749639	-1.335911
H	-3.266830	-0.243390	-2.257600
H	-3.194913	-1.812119	-1.447770
C	-3.696916	-0.147645	-0.127751
C	-1.921311	1.637367	-0.207461
C	-3.465074	1.369487	-0.112287
H	-1.629699	2.003167	-1.193968
H	-1.584739	2.344211	0.553733
H	-3.961832	1.854510	-0.955633
H	-3.859861	1.809287	0.807162
H	2.520935	1.326479	-0.807769
H	4.054617	-0.376660	-1.958853
H	5.139916	1.550548	-0.607033

C	2.607955	0.760785	0.130817
C	4.028665	-0.968243	-1.036808
H	0.412055	0.461190	0.143439
C	5.091960	0.903646	0.275540
C	1.354730	-0.061822	0.280220
H	3.202965	-1.681462	-1.100881
H	2.563790	1.512328	0.933056
C	3.904178	-0.054696	0.181960
H	4.957001	-1.541957	-0.986591
H	6.027364	0.341400	0.326096
H	5.031984	1.539504	1.163194
O	1.272487	-1.227210	0.543271
H	3.871129	-0.673260	1.086652
H	-4.767116	-0.361620	-0.189872

Quin_rgdscan_prod

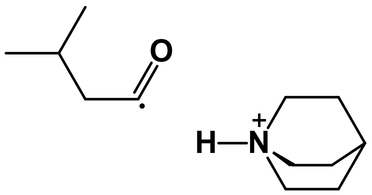

H	-3.793493	-0.661011	2.004833
C	-3.143167	-0.840719	1.146033
H	-2.967259	-1.918334	1.088611
C	-1.813281	-0.088795	1.346797
H	-1.894656	0.721582	2.075057
H	-0.985533	-0.739575	1.631941
N	-1.430925	0.552978	0.037766
C	-1.478315	-0.472361	-1.066094
H	-0.967932	-0.041269	-1.929903
H	-0.897328	-1.325278	-0.710922
C	-2.953968	-0.816622	-1.343170
H	-3.295933	-0.333314	-2.262672
H	-3.052910	-1.894967	-1.484779
C	-3.799132	-0.341788	-0.150745
C	-2.359281	1.695841	-0.273472
C	-3.809977	1.194253	-0.136212
H	-2.121641	2.020538	-1.288968
H	-2.121649	2.507501	0.416390
H	-4.408484	1.588624	-0.960228
H	-4.254221	1.559325	0.794058
H	3.077069	1.581085	-0.735211
H	4.206281	-0.392725	-1.969136
H	5.667749	1.222772	-0.578216
C	3.030515	0.960196	0.167815
C	4.057593	-0.993709	-1.065200
H	-0.460018	0.897992	0.109740
C	5.492380	0.575576	0.287829
C	1.631972	0.403631	0.263455
H	3.097610	-1.510819	-1.144631
H	3.121944	1.654075	1.014169
C	4.131666	-0.112772	0.180751
H	4.841920	-1.753782	-1.039986
H	6.291794	-0.168921	0.313566
H	5.569362	1.185699	1.191935
O	1.268956	-0.702416	0.495299
H	3.974204	-0.737393	1.068276
H	-4.817199	-0.726235	-0.232070

9) References

[1] B. Chen, X. F. Wu, *Org. Lett.* **2019**, *21*, 7624-7629.

[2] P. Colbon, J. Ruan, M. Purdie, J. Xiao, *Org. Lett.* **2010**, *12*, 3670-3673.

[3] S. W. Lee, K. Lee, D. Seomoon, S. Kim, H. Kim, H. Kim, E. Shim, M. Lee, S. Lee, M. Kim, P. H. Lee, *J. Org. Chem.* **2004**, *69*, 4852-4855.

[4] C. F. Malosh, J. M. Ready, *J. Am. Chem. Soc.* **2004**, *126*, 10240-10241.

[5] Y. Sumida, Y. Takada, S. Hayashi, K. Hirano, H. Yorimitsu, K. Oshima, *Chem. - An Asian J.* **2008**, *3*, 119–125.

[6] P. Fan, C. Zhang, L. Zhang, C. Wang, *Org. Lett.* **2020**, *22*, 3875-3878.

[7] F. Caturla, J. M. Jiménez, N. Godessart, M. Amat, A. Cárdenas, L. Soca, J. Beleta, H. Ryder, M. I. Crespo, *J. Med. Chem.* **2004**, *47*, 3874-3886.

[8] X. Zhang, D. W. C. MacMillan, *J. Am. Chem. Soc.* **2017**, *139*, 11353-11356.

[9] M. Jereb, *Green Chem.* **2012**, *14*, 3047-3052

[10] Britton, J. E.; Fang, J.; Heyer, D.; Miller, A. B.; Navas, F. III; Smalley, T. L. Jr.; Zuercher, W. J.; Kalamreddy, S. R. *A preparation of derivatives of cycloalkylidene and (thio)pyranylidene, useful as estrogen receptor modulators*, Patent No. WO2005012220, Publication Date 2005-02-10.

[11] L. Buzzetti, A. Prieto, S. R. Roy, P. Melchiorre, *Angew. Chemie - Int. Ed.* **2017**, *56*, 15039-15043.

[12] L. Cavallo, M. Rueping, B. Maity, C. Zhu, H. Yue, L. Huang, M. Harb, Y. Minenkov, *J. Am. Chem. Soc.* **2020**, *142*, 16942-16952.

[13] M. Yuan, Z. Song, S. O. Badir, G. A. Molander, O. Gutierrez, *J. Am. Chem. Soc.* **2020**, *142*, 7225-7234.

[14] B. J. Shields, A. G. Doyle, *J. Am. Chem. Soc.* **2016**, *138*, 12719-12722.

[15] M. S. Santos, A. G. Corrêa, M. W. Paixão, B. König, *Adv. Synth. Catal.* **2020**, *362*, 2367-2372.

[16] M. J. Frisch, G. W. Trucks, H. B. Schlegel, G. E. Scuseria, M. A. Robb, J. R.

Cheeseman, G. Scalmani, V. Barone, B. Mennucci, G. A. Petersson, H. Nakatsuji, M. Caricato, X. Li, H. P. Hratchian, A. F. Izmaylov, J. Bloino, G. Zheng, J. L. Sonnenberg, M. Hada, M. Ehara, K. Toyota, R. Fukuda, J. Hasegawa, M. Ishida, T. Nakajima, Y. Honda, O. Kitao, H. Nakai, T. Vreven, J. A. Montgomery Jr., J. E. Peralta, F. Ogliaro, M. Bearpark, J. J. Heyd, E. Brothers, K. N. Kudin, V. N. Staroverov, R. Kobayashi, J. Normand, K. Raghavachari, A. Rendell, J. C. Burant, S. S. Iyengar, J. Tomasi, M. Cossi, N. Rega, J. M. Millam, M. Klene, J. E. Knox, J. B. Cross, V. Bakken, C. Adamo, J. Jaramillo, R. Gomperts, R. E. Stratmann, O. Yazyev, A. J. Austin, R. Cammi, C. Pomelli, J. W. Ochterski, R. L. Martin, K. Morokuma, V. G. Zakrzewski, G. A. Voth, P. Salvador, J. J. Dannenberg, S. Dapprich, A. D. Daniels, Ö. Farkas, J. B. Foresman, J. V Ortiz, J. Cioslowski, D. J. Fox, Gaussian 09 Revision C.01. Gaussian Inc. Wallingford CT **2009**.

[17] S. Miertuš, E. Scrocco, J. Tomasi, *Chem. Phys.* **1981**, *55*, 117-129.

[18] S. Miertus̃, J. Tomasi, *Chem. Phys.* **1982**, *65*, 239-245.

[19] J. L. Pascual-ahuir, E. Silla, I. Tuñon, *J. Comput. Chem.* **1994**, *15*, 1127–1138.

[20] H. Jónsson, G. Mills, K. W. Jacobsen, in *Class. Quantum Dyn. Condens. Phase Simulations* (Eds.: B.J. Berne, G. Ciccotti, D.F. Coker), World Scientific, **1998**, pp. 385-404.

[21] G. Henkelman, B. P. Uberuaga, H. Jónsson, *J. Chem. Phys.* **2000**, *113*, 9901-9904.

[22] G. Henkelman, H. Jónsson, *J. Chem. Phys.* **2000**, *113*, 9978-9985.

[23] F. Neese, *Wiley Interdiscip. Rev. Comput. Mol. Sci.* **2012**, *2*, 73-78.

[24] F. Neese, *Wiley Interdiscip. Rev. Comput. Mol. Sci.* **2018**, *8*, 1-6.
